# Receptor Polymorphism and Genomic Structure Interact to Shape Bitter Taste Perception

**DOI:** 10.1371/journal.pgen.1005530

**Published:** 2015-09-25

**Authors:** Natacha Roudnitzky, Maik Behrens, Anika Engel, Susann Kohl, Sophie Thalmann, Sandra Hübner, Kristina Lossow, Stephen P. Wooding, Wolfgang Meyerhof

**Affiliations:** 1 German Institute of Human Nutrition Potsdam-Rehbruecke, Department of Molecular Genetics, Nuthetal, Germany; 2 Health Sciences Research Institute, University of California, Merced, California, United States of America; National Institute of Genetics, JAPAN

## Abstract

The ability to taste bitterness evolved to safeguard most animals, including humans, against potentially toxic substances, thereby leading to food rejection. Nonetheless, bitter perception is subject to individual variations due to the presence of genetic functional polymorphisms in bitter taste receptor (*TAS2R*) genes, such as the long-known association between genetic polymorphisms in *TAS2R38* and bitter taste perception of phenylthiocarbamide. Yet, due to overlaps in specificities across receptors, such associations with a single *TAS2R* locus are uncommon. Therefore, to investigate more complex associations, we examined taste responses to six structurally diverse compounds (absinthin, amarogentin, cascarillin, grosheimin, quassin, and quinine) in a sample of the Caucasian population. By sequencing all bitter receptor loci, inferring long-range haplotypes, mapping their effects on phenotype variation, and characterizing functionally causal allelic variants, we deciphered at the molecular level how a subjects’ genotype for the whole-family of *TAS2R* genes shapes variation in bitter taste perception. Within each haplotype block implicated in phenotypic variation, we provided evidence for at least one locus harboring functional polymorphic alleles, e.g. one locus for sensitivity to amarogentin, one of the most bitter natural compounds known, and two loci for sensitivity to grosheimin, one of the bitter compounds of artichoke. Our analyses revealed also, besides simple associations, complex associations of bitterness sensitivity across TAS2R loci. Indeed, even if several putative loci harbored both high- and low-sensitivity alleles, phenotypic variation depended on linkage between these alleles. When sensitive alleles for bitter compounds were maintained in the same linkage phase, genetically driven perceptual differences were obvious, e.g. for grosheimin. On the contrary, when sensitive alleles were in opposite phase, only weak genotype-phenotype associations were seen, e.g. for absinthin, the bitter principle of the beverage absinth. These findings illustrate the extent to which genetic influences on taste are complex, yet arise from both receptor activation patterns and linkage structure among receptor genes.

## Author Summary

Human bitter taste is believed to protect us from the ingestion of poisonous substances, thereby shaping food rejections. Bitter perception differs, however, across individuals, due to genetic variations in the ~25 bitter taste receptor (*TAS2R*) genes. A famous example known since the 1930s is the inherited bitter taste sensitivity to phenylthiocarbamide, which is associated with genetic polymorphisms in a single *TAS2R* gene. Yet, such simple receptor-substance associations do not reflect the full complexity of bitter perception, since individual bitter substances frequently activate several TAS2Rs. Here, we provide an in-depth analysis of the genetic variability influencing human bitter taste. While each study subject carried a different set of genetic polymorphisms, we found that most variations reside in just six blocks, each harboring only one to five haplotypes. Thus, besides simple associations between taste and *TAS2R* gene polymorphisms, we revealed complex associations dependent on linkage between several high- and low-sensitivity alleles. Indeed, subjects carried either sensitive or insensitive alleles for receptors sensitive to grosheimin, a bitter compound in artichoke, or at least one sensitive allele for receptors specific for absinthin, the bitter principle of absinth. In short, simple associations and complex genomic linkage determine sensitivity to selected dietary bitter compounds.

## Introduction

Bitter taste perception plays a fundamental role in dietary preferences and behaviours, by shaping aversions to foods and drinks. Indeed, averse responses to bitterness are instinctive and drive rejection and avoidance behaviours widely observed in animal models, but also in human infants [1–5]. They are hypothesized to originate with bitter perception’s role as a warning sensor against potentially harmful substances contained in the diet, such as toxins released by plants to deter herbivores, and they belong to diverse chemical classes including acetogenins, alkaloids, flavonoids, phenylpropanes, terpenoids, and thiol compounds [6–9]. These rejection behaviours mediated by bitter perception show evolutionary trends, with responses depending on the occurrence of bitter substances in animal typical diets [10]. Occasionally, bitter substances known to possess desirable pharmacological activities are also deliberately ingested (e.g., [11, 12]), nevertheless acceptation of bitter phytonutrient in food remains challenging (for review see [13]).

Despite the importance of bitter taste in shaping nutritional behaviours and guarding against toxin ingestion, bitter responses in humans vary profoundly. The classic example of phenotypic diversity in humans is threshold sensitivity to phenylthiocarbamide (PTC), which differs by up to 10,000-fold among individuals. Such variation is due to a constellation of interacting factors including environmental effects, age, gender, experience and genetics (for reviews see [13–15]). Particularly strong effects have been found to arise from polymorphism in *TAS2R* genes, which encode a series of ~25 G protein-coupled receptors expressed in taste buds [16–20]. In the case of PTC perception, polymorphism in *TAS2R38* accounts for more than 55% of observed phenotypic variance [17, 21]. Moreover, genetic polymorphisms occurring at *TAS2R* loci are common, with numerous high-frequency alleles [22], suggesting the presence of functionally important receptor variants. Major changes in receptor activity due to such variants have been observed in a few other cases, i.e., TAS2R9, TAS2R16, TAS2R43, and TAS2R31 [23–27]. In addition, gene association studies suggested functional polymorphic alleles at other TAS2R loci, e.g. TAS2R4 or TAS2R13 [28–30].

An essential aspect of interactions between TAS2Rs and bitter compounds relates to the overlapping agonist profiles, with most TAS2Rs responding to multiple agonists and many agonists stimulating multiple receptors [31]. This combinatorial activation pattern, together with the distribution of *TAS2R* genes among only four cytogenic locations, which can lead to false-positive genotype-phenotype associations arising from sites in linkage with the causal variants, have so far prevented the full elucidation of bitter perception’s molecular underpinnings [26, 32–34]. To establish a genetic basis for the observed perceptual differences in the population, we used in this study an integrative approach, sequencing all known *TAS2R* loci in humans, inferring long-rang haplotypes, mapping their effects on perception of several chemically diverse compounds, and functionally characterizing all allelic variants associated with shifts in perception.

## Results

### SNP and haplotype diversity

**Fig 1:**
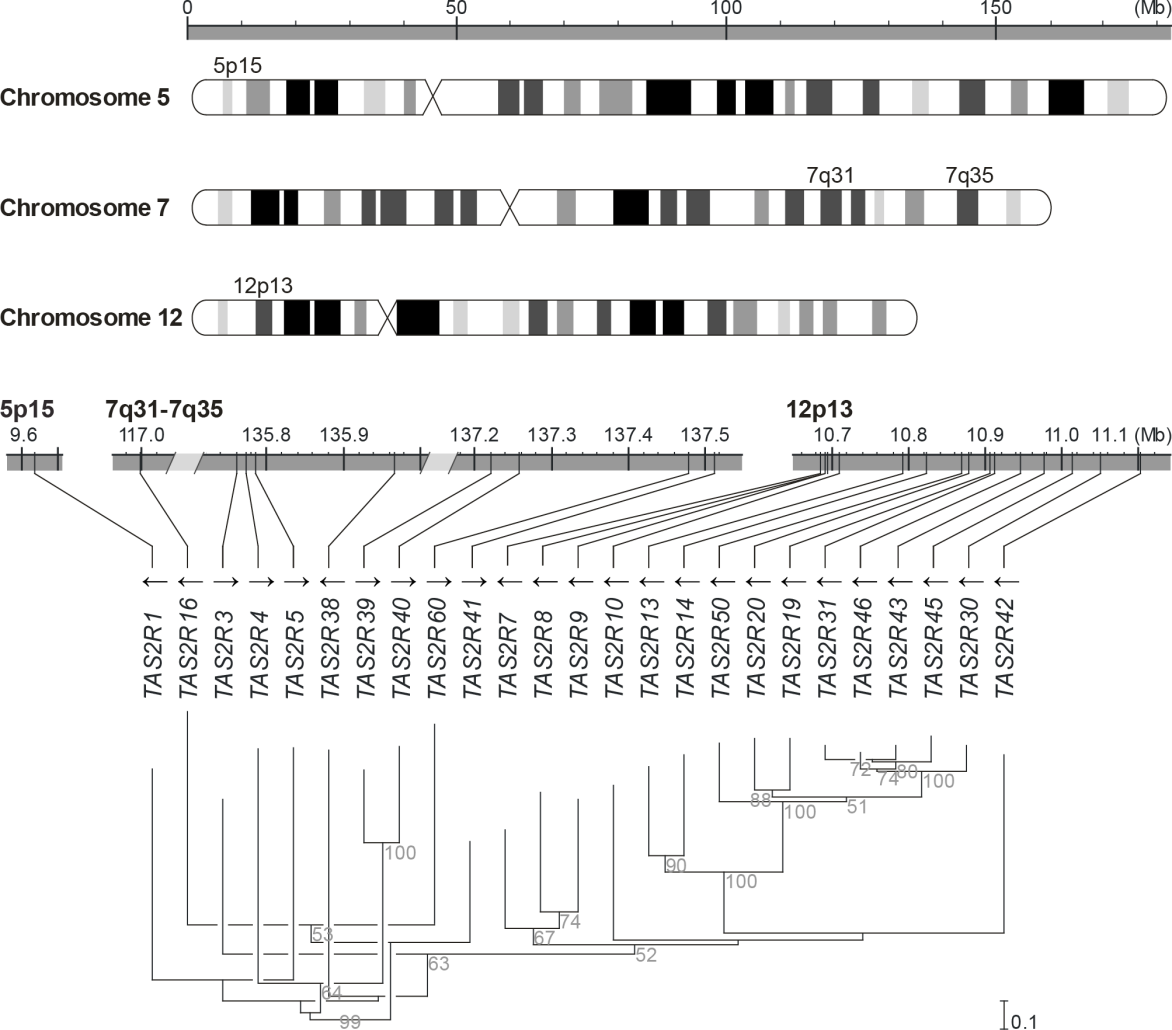
Genomic organisation and phylogenetic tree of the *TAS2R* gene family. *TAS2R* genes are distributed among the cytogenetic locations 5p15, 7q31-7q35 and 12p13 on chromosome 5, 7 and 12, as shown on the ideogram of the G-banding pattern at the 850 band resolution [35]. One gene, *TAS2R1*, is located at 5p15. Nine are located on chromosome 7 in a ~20.5 Mb region spanning the cytogenetic location 7q31-7q35, which contains *TAS2R16* and, separated by a ~18.8 Mb distance on the same chromosome, eight other *TAS2R* genes: *TAS2R3*, *-4*, *-5*, *-38*, *-39*, *-40*, *-60*, and *-41*. Fifteen *TAS2R* genes are located on chromosome 12 in a ~400 kb region at 12p13: *TAS2R42*, *-30*, *-45*, *-43*, *-46*, *-31*, *-19*, *-20*, *-50*, *-14*, *-13*, *-10*, *-9*, *-8*, and *-7*. Evolutionary analyses indicated that genomic location is associated with phylogenetic affiliation, as previously published [20].

**Fig 2:**
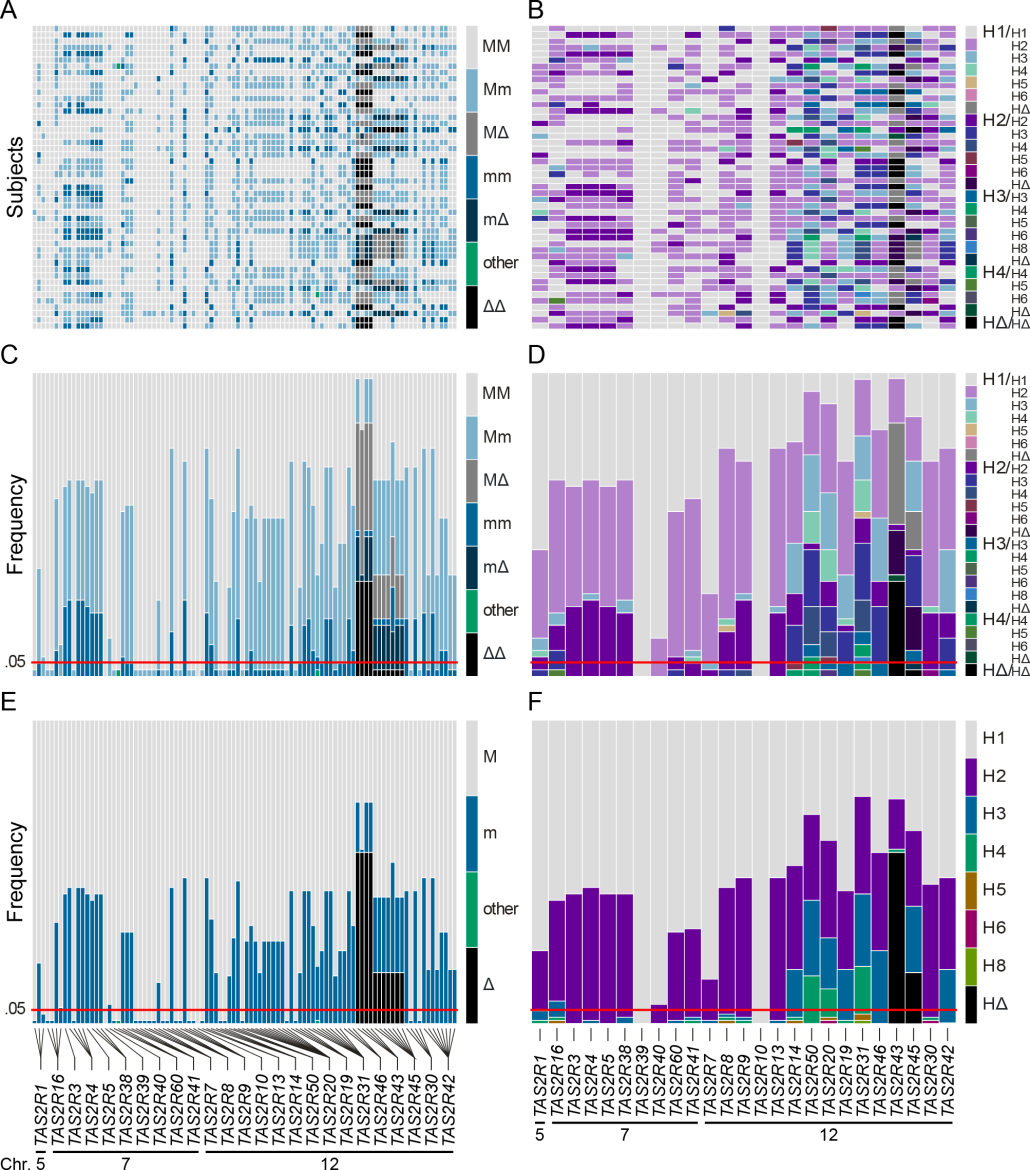
Coding region SNPs, genotypes and haplotypes of the 25 *TAS2R* genes. Light grey boxes indicate homozygous major genotypes or haplotypes, i.e., most common; black boxes, homozygous deleted genotypes or haplotypes, i.e., whole-gene deletion; each other colour box specifies a different genotype or haplotype, homozygous or heterozygous, as detailed in the colour bar. Haplotypes were named according to previous nomenclature or according to their respective allele frequencies in our subjects; the most common haplotypes receiving the smallest number [26]. Chromosomal assignments are specified for cSNPs and *TAS2R* loci. (A) cSNP genotypes by subject; (B) *TAS2R* genotypes by subject; (C) genotype frequency by cSNP site; (D) genotype frequency by *TAS2R* locus; (E) allele frequency by cSNP site; (F) haplotype frequency by *TAS2R* locus.

Genetic diversity was assessed by determining whole-gene genotypes of the 25 members of the *TAS2R* gene family and corresponding copy number variations (Figs 1 and 2, S1 Table). Across the 48 Caucasian subjects, a total of 93 coding SNPs (cSNPs), including 65 missense SNPs and 2 nonsense SNPs, were identified. Three indels, including 1 rare three-nucleotide in-frame deletion and 2 major deletions spanning *TAS2R43* or *TAS2R45* locus, were also detected. Genes harboured a mean of 4 cSNPs with a mean of ~1 synonymous and ~3 non-synonymous SNPs per gene. The mean number of haplotypes within genes was 3.5 with a range of 1 to 6, which recombined to form a mean number of 5.6 genotypes within genes with a range of 1 to 13.

When only common cSNPs were considered (i.e., with frequency ≥ 0.05), a total of 67 SNPs, including 45 missense SNPs and 2 nonsense SNPs, and 2 indels were identified, all of which have been reported previously [22, 26, 36–40]. Sequenced genes harboured a mean of ~1 synonymous and ~2 non-synonymous common SNPs per gene. While some genes harboured no SNPs (e.g., *TAS2R10* and *TAS2R39*), others harboured many. Genes located in the proximal region of the cluster at 12p13 harboured also highest number of SNPs, with 8 at *TAS2R31* and *-42*, and 9 at *TAS2R20*.

**Fig 3:**
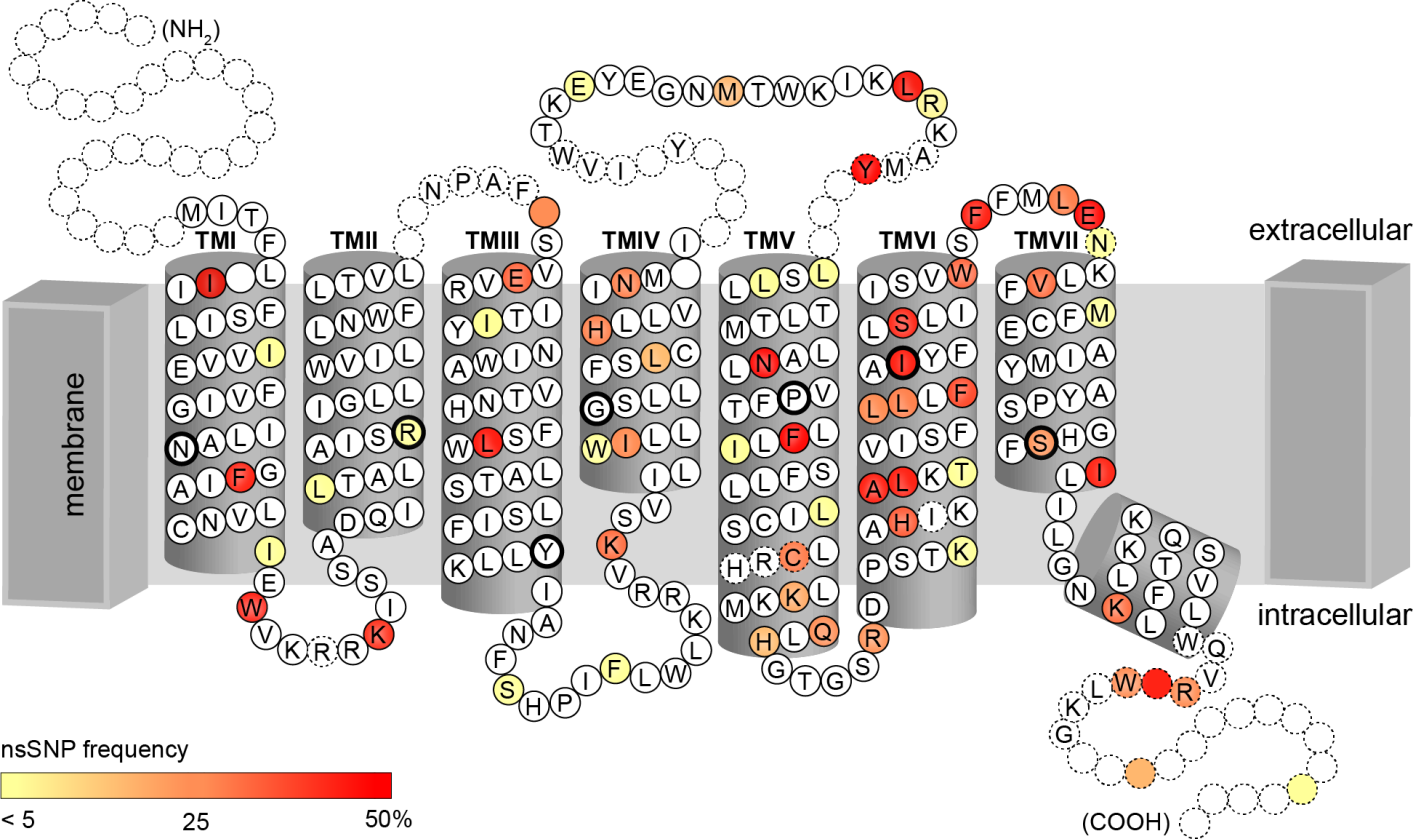
Identified amino acid positions corresponding to cSNPs represented on the snake plot of the consensus sequence. Highly conserved amino acids among TAS2Rs, indicated by black circles, occur primarily in inner transmembrane domains [41, 42]. Colour scale corresponds to allele frequency in our sample.

Of these 45 common missense cSNPs, 27 corresponded to amino acid positions localised in TAS2R transmembrane (TM) domains, with 9 SNPs affecting amino acids in TM VI and 6 in TM V (Fig 3). The remaining SNPs were distributed in sequence areas coding for extracellular domains, intracellular domains, and COOH terminal region, with 8, 5, and 5 SNPs, respectively. In addition, 2 nonsense cSNPs result in a premature stop codon in the reading frame and therefore to a putatively non-functional, truncated receptor variant.

These common cSNPs recombined across genes to form a total of 59 coding haplotypes, with a mean of 2.2 per gene and a range of 1 to 4. Again, genes localised to the proximal region of 12p13 were most diverse, with 4 haplotypes at *TAS2R20*, *-31*, and *-50*. As with common SNPs, most within-gene haplotypes have been reported previously [22, 26, 36–40]. Two common haplotypes were newly identified: one at *TAS2R14* (frequency = 0.34), and one at *TAS2R42* (frequency = 0.30).

### Long-range haplotypes

High levels of genetic diversity were observed although our population sample, only Caucasian subjects, was relatively homogeneous with respect to ethnic and geographic origin. Thus, chromosomal spatial relations, linkage and block structures among genes might be important contributors to *TAS2R*-mediated phenotypes. Indeed, the 25 members of the *TAS2R* gene family are restricted to just three cytogenetic locations 5p15, 7q31-7q35, and 12p13 (Fig 1). Position 5p15 contains a single gene, *TAS2R1*, 7q31-35 contains 9 genes distributed across a ~20.5 Mb region, and 12p13 contains 15 genes distributed across a ~400 kb region. Further, most of the *TAS2R*s at 7q31-7q35 (*TAS2R3*, *-4*, *-5*, *-38*, *-39*, *-40*, *-60*, and *-41)* reside in a 1.8 Mb sub-region.

**Fig 4:**
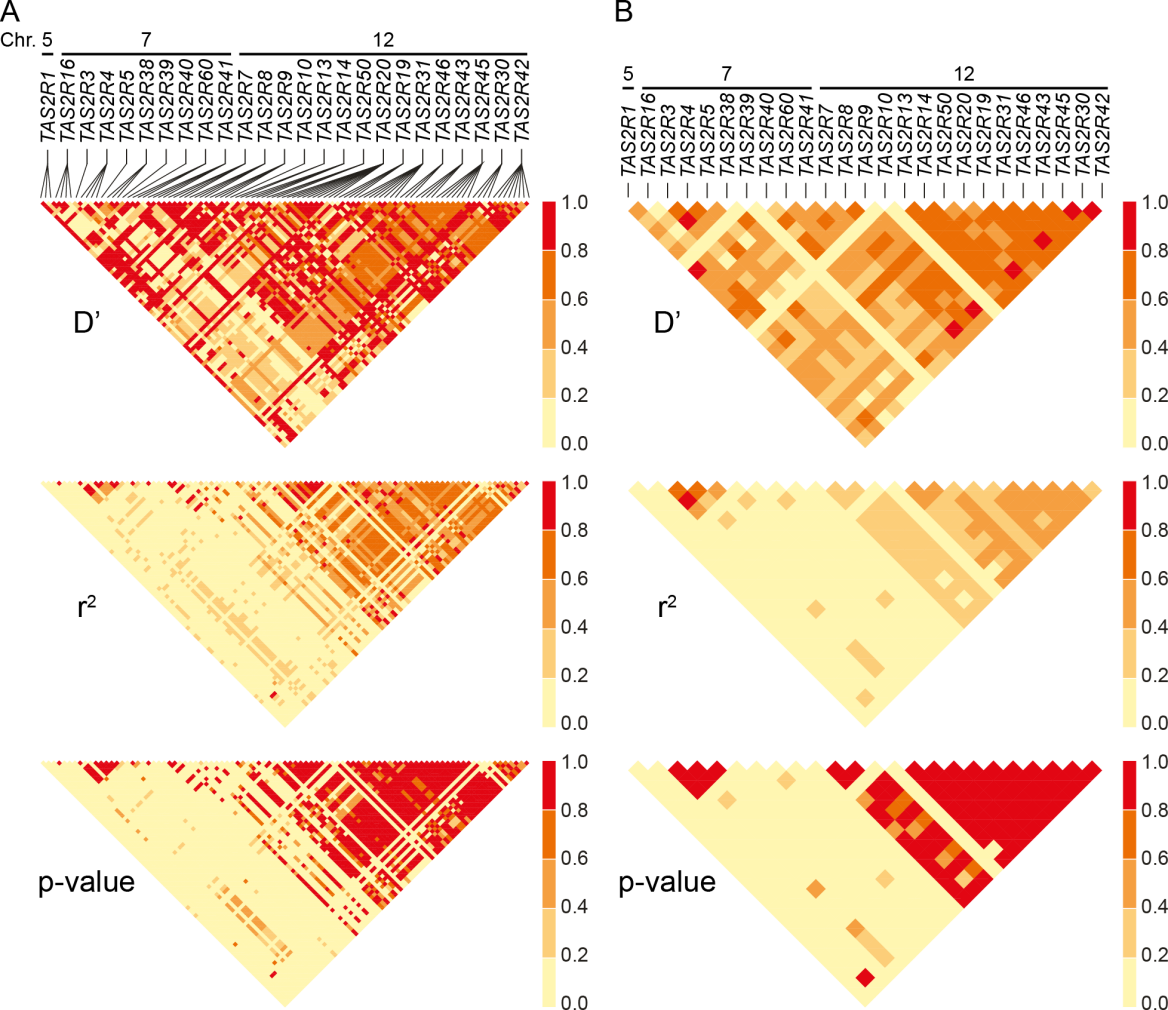
Linkage disequilibrium across SNPs and genes. (A) Pairwise D’, r^2^ and corresponding p-values between cSNPs, and (B) between *TAS2R*s. Chromosomal assignments are specified. Long-range haplotypes identified in block analyses are located in the conspicuous high LD regions of *TAS2R7*-*TAS2R42*, and *TAS2R3*-*TAS2R38*.

Estimates of pairwise D′ and r^2^ revealed extensive linkage in both the 7q31-7q35 and 12p13 regions. Mean values of D′ and r^2^ between common cSNPs were 0.66 and 0.52 across 12p13, and associated with low p-values (Fig 4). Nearly identical trends were observed between *TAS2R* loci, with mean D′ and r^2^ values of 0.66 and 0.37. Similarly, across the 1.8 Mb *TAS2R3-38* cluster, mean values were 0.76 and 0.74 for common cSNPs and 0.60 and 0.63 between *TAS2R* loci. These trends indicate that *TAS2R*s are tightly connected in long-range haplotypes spanning multiple genes.

**Fig 5:**
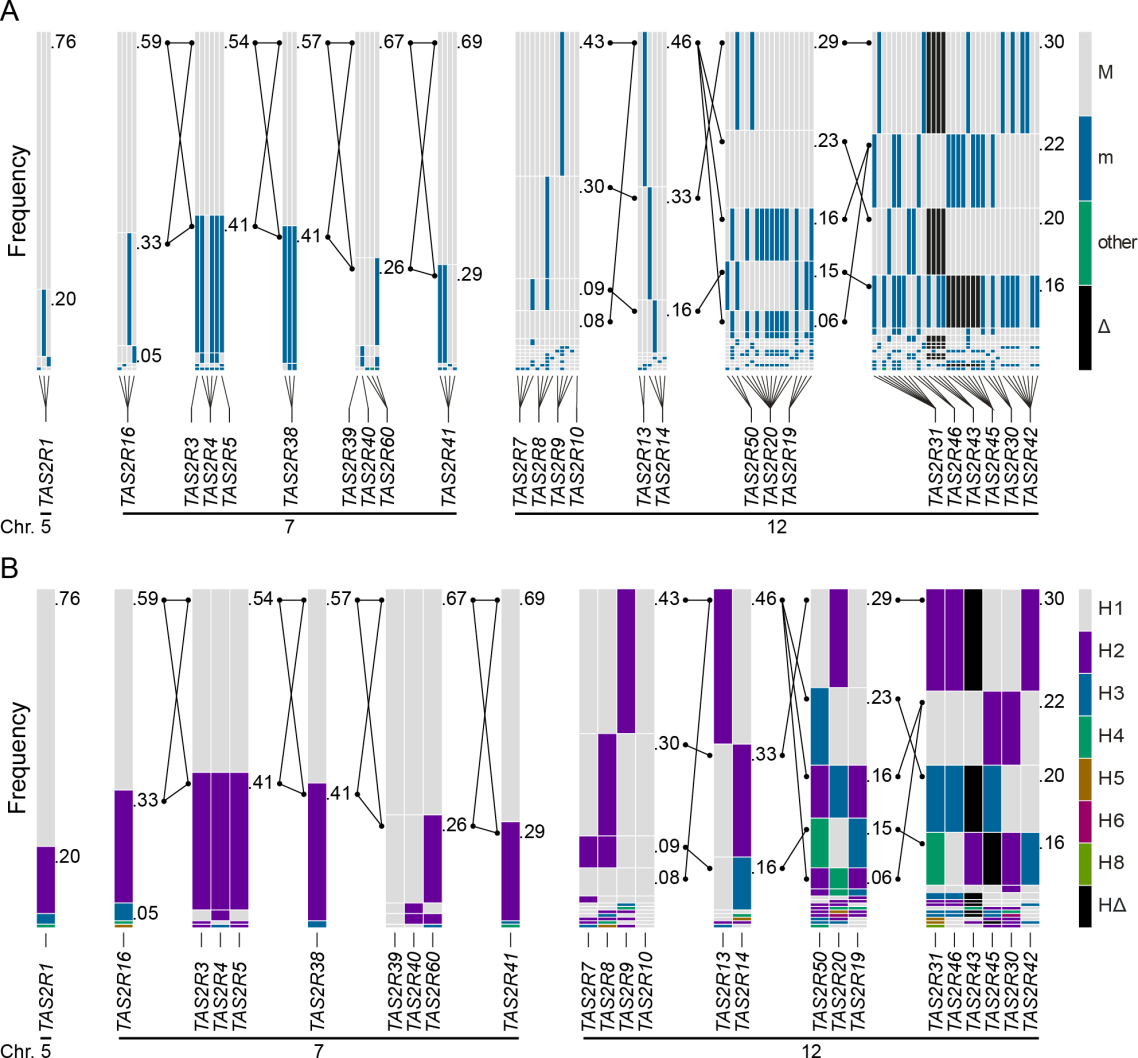
Haplotype block structure, common haplotype and long-range haplotype (LRH) across the *TAS2R* genes regions on chromosome 5, 7, and 12 parsed using (A) cSNPs and (B) *TAS2R* genes. Long-range multilocus haplotypes were phased for the *TAS2R3-5* and the *TAS2R39-60* regions on chromosome 7; and the *TAS2R7-10*, the *TAS2R13-14*, the *TAS2R50-19*, and the *TAS2R31-42* regions on chromosome 12. They are displayed with their corresponding frequency and connected *TAS2R* haplotypes with lines if the minor frequency is greater than 0.05. For the *TAS2R3-5* and the *TAS2R39-60* blocks, two common long-rang haplotypes with a frequency of more than or equal to 0.05 were inferred. The *TAS2R7-10*, the *TAS2R13-14*, the *TAS2R50-19*, and the *TAS2R31-42* harbour 4, 3, 5, and 4 long-range haplotypes, respectively.

Haplotype block partitions and corresponding long-range haplotypes were then determined for the 25 *TAS2R* genes (Fig 5). Analyses inferring haplotype blocks identified six blocks distributed across the *TAS2R* clusters on chromosomes 7 and 12. These corresponded to blocks found in the region by previous studies [34, 43, 44]. Two were found on chromosome 7, encompassing the *TAS2R3-5* and *TAS2R39-60* regions, each of which harboured two long-range haplotypes with frequencies at or above 0.05. Four blocks were found on chromosome 12, encompassing the *TAS2R7-10*, *TAS2R13-14*, *TAS2R50-19*, and *TAS2R31-42* regions, which harboured 1, 3, and 5 long-range haplotypes respectively. In addition, whereas blocks on chromosome 7 were flanked by recombination hot spots, we identified no hot spots in the *TAS2R13-42* region on chromosome 12, which seems to indicate that these latter haplotype blocks are determined by linkage disequilibrium decay [45].

### Genotype-phenotype associations

Genotype-phenotype association analyses were conducted to elucidate genetic liability for taste phenotypes; phenotype data consisting of individual detection and recognition thresholds for the bitter tastants, as well as concentrations for perceived weak, moderate, strong, and very strong intensities inferred from intensity ratings. Subsequent functional assays provided further insights into the identification of causal TAS2Rs.

#### Amarogentin

**Fig 6:**
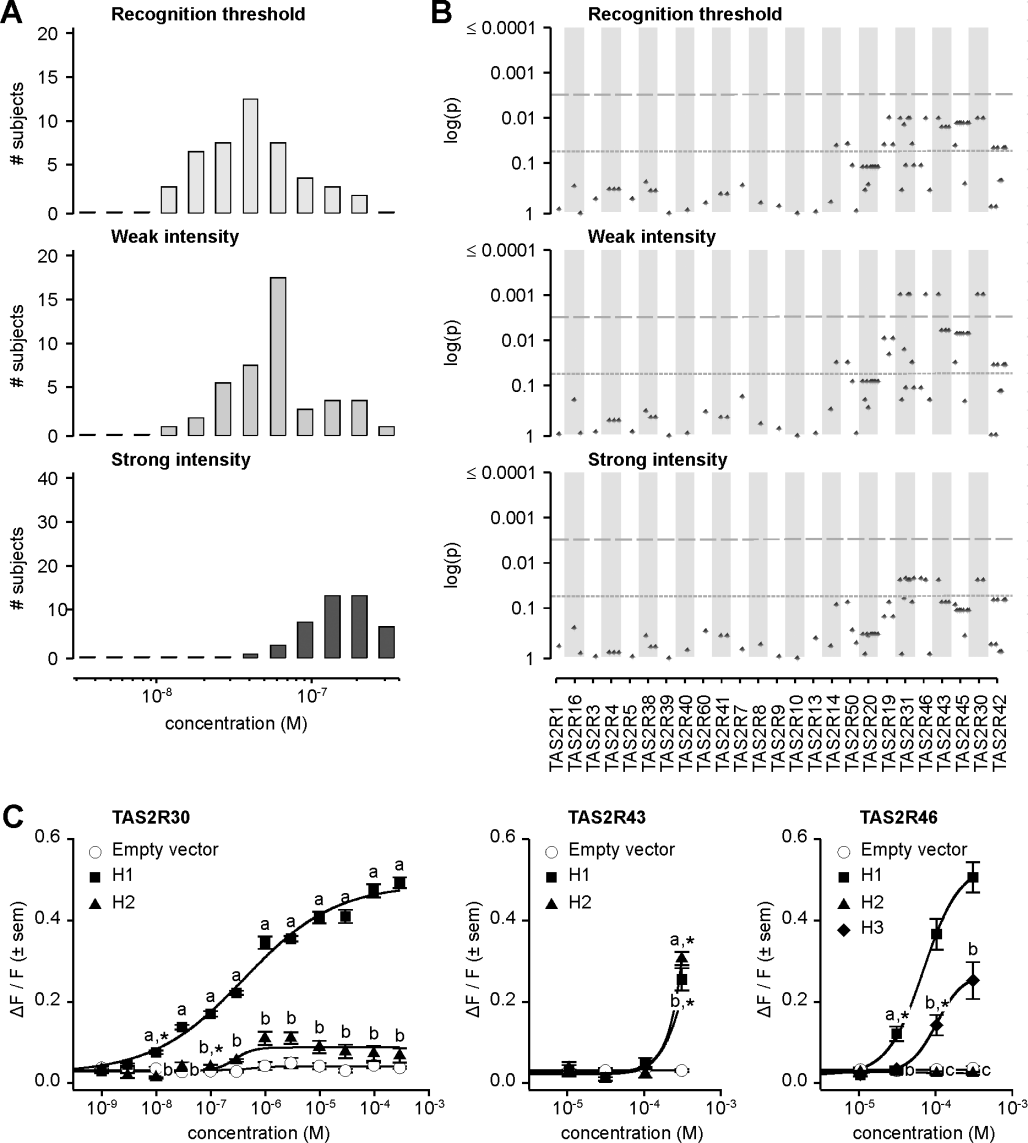
Amarogentin phenotypes, genotype-phenotype associations, and functional assays. (A) Distribution of bitter taste recognition thresholds, and concentrations perceived as weak and strong. (B) Genotype-phenotype associations for common SNPs (frequency > 0.05). Dashed line indicates significance threshold following corrections for multiple testing. Solid line indicates uncorrected significance threshold. (C) Allele-specific dose response curves for loci harboring SNPs showing significant phenotypic associations.

Bitter taste responses to amarogentin exhibited high variance across subjects on both threshold and suprathreshold measures (Fig 6A). Recognition thresholds ranged 11-fold across subjects, with the most sensitive individuals reporting bitterness at a concentration of 1.2x10^-8^ M, the least sensitive reporting it at 2.0x10^-7^ M, and a modal number at 4.0x10^-8^ M. Concentrations rated as weakly bitter by subjects ranged 17-fold, from 1.2x10^-8^ to 3.0x10^-7^ M with a mode at 5.0x10^-8^ M. In contrast to recognition thresholds and concentrations perceived as weak, the distribution of concentrations perceived as strongly bitter was narrow, ranging less than 4-fold, from 6.0x10^-8^ to 3.0x10^-7^ M and a mode between 1.4x10^-7^ and 2.0x10^-7^ M. Distributions of concentrations perceived as moderately and very strongly bitter exhibited analogous properties (S2 Fig).

Association analyses targeting perceived weak intensity of amarogentin revealed significant signals localized to the region spanning *TAS2R31* to *TAS2R42*, with the lowest *p*-values occurring at *TAS2R30*, *-31*, *-43*, and *-46* (Fig 6B; S2 Table). Associations with recognition threshold and perceived strong intensity showed similar patterns. However, while significant at the 0.05 level, these did not reach significance at the experiment-wide significance threshold, i.e., when corrected for multiple testing. Association analyses targeting detection thresholds, perceived moderate intensity, and very strong intensity yielded similar results (S2 Fig; S2 Table). Numerous non-synonymous variants in the *TAS2R31*-*42* block were thus observed among the variants significantly associated with amarogentin perception, indicating that phenotypes are likely driven by polymorphism in the corresponding *TAS2R*s. However, within-block LD across the implicated sites was high, raising the possibility that in addition to true associations arising from functional effects, spurious associations were present as the result of linkage between contributory and irrelevant sites. Numerous SNPs in *TAS2R*s at 12p13 were also significant at 0.05, but not when corrected. These patterns might be also explained by high within-block LD, accompanied by between-block linkage decay which reduces spurious association signals with distance. Hence, the mechanistic properties of these variants could not be discerned from genotype-phenotype association data alone. To resolve these ambiguities we examined substitution effects using an *in vitro* molecular approach.

In a previous study, we utilized heterologous expression assays to ascertain the response of every known TAS2R in humans to a chemical library of synthetic and natural compounds [31]. The resulting inventory identified six TAS2Rs showing baseline responses to amarogentin: TAS2R1, -4, -39, -43, -46, -30, and -50. Thus, three TAS2Rs (TAS2R43, -46, and -30) previously shown to exhibit baseline responses to amarogentin also harboured SNPs associated with amarogentin sensitivity in our subjects, specifically implicating these sites as sources of variation in both receptor function and downstream measures of amarogentin sensitivity.

*In vitro* assays revealed extensive variation in receptor response arising from substitutions associated with amarogentin perception phenotypes (Fig 6C). TAS2R30 harboured two common receptor variants (i.e., with frequency ≥ 0.05) exhibiting divergent responses, TAS2R30-H1 and-H2. TAS2R30-H1 was highly sensitive and responsive to amarogentin (threshold = 1.0x10^-8^ M; EC_50_ = 4.1x10^-7^ M; maximum amplitude = 0.50 ΔF/F) while TAS2R30-H2 was less sensitive, showing a slightly reduced threshold and severely reduced maximal signal amplitude (threshold = 3.0x10^-7^ M; EC_50_ = 3.9x10^-7^ M; maximum amplitude = 0.12 ΔF/F). In contrast to TAS2R30, TAS2R43 variants exhibited essentially no response to amarogentin, with both TAS2R43-H1 and-H2 only being activated at the highest artefact-free concentration of 3.0x10^-4^ M. Receptor variants of TAS2R46 exhibited major differences in amarogentin sensitivity, nonetheless only from the relatively high concentration of 3.0x10^-5^ M. TAS2R46-H1 was highly sensitive and responsive (threshold = 3.0x10^-5^ M; EC_50_ = 6.7x10^-5^ M; maximum amplitude = 0.57 ΔF/F), whereas TAS2R46-H3 was moderately activated (threshold = 1.0x10^-4^ M). TAS2R46-H2, which is characterized by a premature stop codon resulting in a severe truncation of the receptor, was not activated at any concentration.

Patterns of activation across TAS2R30, -43, and -46 variants suggest that bitter taste sensitivity to amarogentin is likely solely driven by functional genetic polymorphisms in *TAS2R30*. Of all tested receptor variants, only TAS2R30-H1 was highly responsive to amarogentin at concentrations matching thresholds in subjects. Further, the second receptor variant of TAS2R30,-H2, exhibited weak activation regardless the concentration range, explaining the presence of low-sensitivity subjects and overall patterns of association. In contrast to TAS2R30, activation of TAS2R43 or TAS2R46 occurred at concentrations 3000-fold or 300-fold higher than TAS2R30, respectively, and far exceeding observed threshold phenotypes. TAS2R43 and TAS2R46 failed thus to explain observed patterns of phenotypic variation.

#### Quassin

Like amarogentin, genotype-phenotype associations and functional analyses indicate that functional genetic polymorphisms in *TAS2R30* are also responsible for the bitter taste sensitivity of quassin, as expected by the high value of the Pearson’s correlation coefficient between these two tastants (0.87; p < 0.001). As with amarogentin, distributions of threshold recognition and concentrations perceived as weak were broad, ranging 38-fold (from 2.6x10^-8^ M to 1.0x10^-6^ M) and 26-fold (from 3.9x10^-8^ M to 1.0x10^-6^ M), respectively, while distribution of concentrations perceived as strong was narrow, ranging 8-fold (from 1.3x10^-7^ M to 1.0x10^-6^ M) (S3 Fig). *In vitro*, activation pattern of TAS2Rs differ slightly between the two chemically distinct tastants amarogentin and quassin, with quassin activating only TAS2R30 and TAS2R46. TAS2R30-H1 and-H2 exhibited divergent responses to quassin, with-H1 responding in ranges matching phenotypic recognition threshold. TAS2R46 variants also exhibited divergent activation patterns, with TAS2R46-H1 being most sensitive,-H3 being intermediate, and-H2 being least sensitive, but these responded at concentrations exceeding phenotypic recognition thresholds, suggesting that they are unable to explain observed phenotypes.

#### Grosheimin

**Fig 7:**
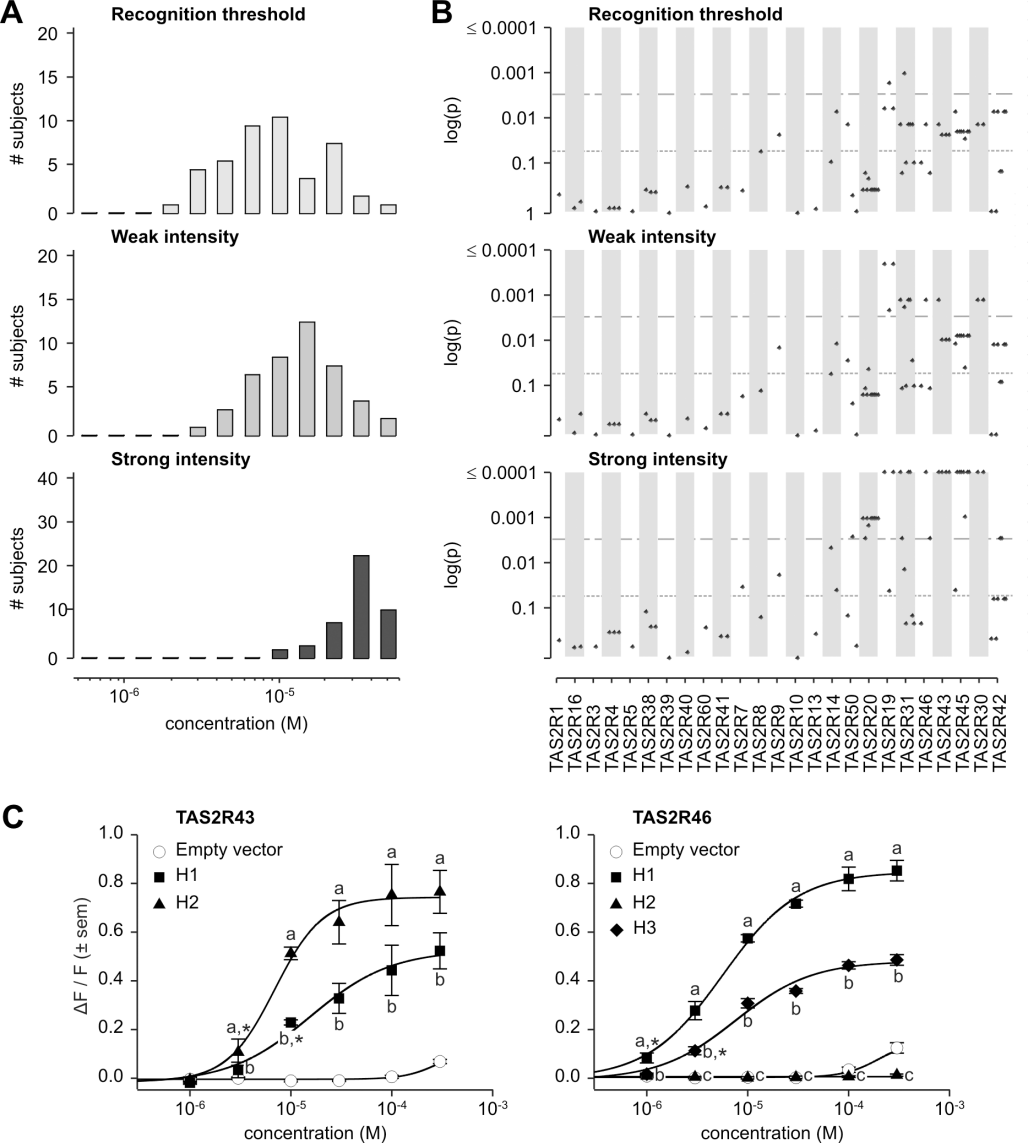
Grosheimin phenotypes, genotype-phenotype associations, and functional assays. (A) Distribution of bitter taste recognition thresholds, and concentrations perceived as weak and strong. (B) Genotype-phenotype associations for common SNPs (frequency > 0.05). Dashed line indicates significance threshold following corrections for multiple testing. Solid line indicates uncorrected significance threshold. (C) Allele-specific dose response curves for loci harboring SNPs showing significant phenotypic associations.

Taste responses to grosheimin exhibited broad phenotypic distributions (Fig 7A). Recognition thresholds ranged 25-fold, from 2.0x10^-6^ M to 5.0x10^-5^ M, with distinct modes at 9.9x10^-6^ M and 2.2x10^-5^ M. Bimodality is a classic feature of threshold responses to bitter substances including phenylthiocarbamide (PTC), propylthiouracil (PROP), goitrin, saccharin, acesulfame potassium, and aloin, suggesting that genetic effects on grosheimin response are likely strong [25, 26, 46]. The distribution of concentrations corresponding to perceived weak intensity was also broad, ranging from 2.9x10^-6^ M to 5.0x10^-5^ M (17-fold) with a single mode at 1.5x10^-5^ M. In contrast, the distribution of concentrations corresponding to perceived strong intensity was narrow, ranging only 5-fold (from 9.9x10^-6^ M to 5.0x10^-5^ M) with a single mode at 3.3x10^-5^ M. Distribution of detection thresholds was similar to that of recognition threshold; distributions of concentrations perceived as weak, moderate, strong, and very strong changed gradually from a broad to a relatively narrow phenotypic distribution (S2 Fig).

SNPs significantly associated with grosheimin response at the experiment-wide significance threshold were found at multiple loci, depending on the specific phenotype (Fig 7B; S2 Table). Two loci, *TAS2R19* and -*31*, harboured polymorphisms associated with recognition threshold. In addition to being found at *TAS2R19* and -*31*, SNPs significantly associated with weak intensity occurred at *TAS2R30* and -*46*; the whole-gene deletion at *TAS2R43* was also significant. Besides these associations, numerous SNPs spanning the *TAS2R50*-*19* and *TAS2R31*-*42* haplotype blocks were associated with strong intensity response. Additional SNPs in *TAS2R* genes located at 12p13 were also significant at the 0.05 level for recognition threshold, perceived weak and strong intensities, i.e., uncorrected for multiple testing. Tests for association with detection threshold, and perceived moderate and very strong intensities were consistent with these observations (S2 Fig; S2 Table). As previously mentioned for amarogentin and quassin, LD was high between SNPs associated with grosheimin response, leaving the specific causative sites unclear. However, in our published inventory of receptor-agonist relationships across the complete TAS2R family, only TAS2R43 and -46 were activated by grosheimin, and both harboured variants associated with grosheimin perception in our subjects [31].

*In vitro* assays targeting TAS2R43 and -46 confirmed the presence of functional variation at these loci (Fig 7C). TAS2R43 harboured two receptor variants, TAS2R43-H1 and-H2, both of which were responsive to grosheimin. However, TAS2R43-H2 was responsive at lower concentrations than did-H1 (threshold = 3.0x10^-6^ M; EC_50_ = 6.9x10^-6^ M vs. threshold = 1.0x10^-5^ M; EC_50_ = 1.6x10^-5^ M), and exhibited stronger responses (maximum amplitude = 0.81 ΔF/F vs. 0.60 ΔF/F). Both receptor variants were activated in the concentration range perceived as bitter by human subjects. A third allele of *TAS2R43* characterized by complete deletion of the gene (*TAS2R43*-Δ) was also found at high frequencies, suggesting that it could be an important driver of phenotypic associations [25, 26]. Among the three observed TAS2R46 variants, TAS2R46-H1 was highly responsive to grosheimin (threshold = 1.0x10^-6^ M; EC50 = 5.6x10^-6^ M; maximum amplitude = 0.87 ΔF/F), and-H3 was moderately responsive (threshold = 3.0x10^-6^ M; EC50 = 7.6x10^-6^ M; maximum amplitude = 0.50 ΔF/F). TAS2R46-H2, a truncated, six-transmembrane variant of the receptor, was not activated at any concentration. Moreover, responsive TAS2R43 and -46 variants were activated at concentrations perceived as bitter by human subjects. Thus, both receptors contributed to the variance of grosheimin perception.

A key aspect of variation in *TAS2R43* and *-46* was that linkage disequilibrium spanned gene loci, such that their functional and phenotypic contributions were non-independent. Inspection of the long-range haplotypes in the *TAS2R31-42* block (Fig 5) revealed that the most common haplotype (frequency = 0.30) harboured both the deleted allele at *TAS2R43* and the truncated non-functional allele at *TAS2R46*. In contrast, all other haplotypes harboured sensitive and non-sensitive alleles in varying combinations. The second most common (frequency = 0.22) harboured a moderately sensitive (*TAS2R43*-H1) and a sensitive allele (*TAS2R46*-H1), and the third most common (frequency = 0.20) a deleted (*TAS2R43*-Δ) and a moderately sensitive allele (*TAS2R46*-H3). A final haplotype (frequency = 0.16) harboured two sensitive alleles (*TAS2R43*-H2 and *TAS2R46*-H1). Thus, while *TAS2R43* and *-46* make functionally independent contributions to phenotype they co-segregate, resulting in positively correlated associations.

#### Quinine

Quinine elicited also phenotypic responses driven by variation in multiple *TAS2Rs*. Threshold recognition exhibited a broad distribution (25-fold, from 1.6x10^-6^ M to 4.0x10^-5^ M), followed by perceived weak intensity (17-fold, from 2.3x10^-6^ M to 4.0x10^-5^ M). The distribution of perceived strong intensity was narrower (5-fold, from 7.9x10^-6^ M to 4.0x10^-5^ M) (S3 Fig). However, our genotype-phenotype associations failed, in line with previously reported studies, to identify SNPs explaining phenotypic variation, due to the high linkage disequilibrium spanning functionally distinct *TAS2R* loci [32, 33]. *In vitro*, quinine elicited numerous TAS2R-mediated responses, with TAS2R30, -31, -43, and -46 responding only at the second or the last artefact-free concentration (1.0x10^-5^ M or 3.0x10^-5^ M). Receptor variants of TAS2R30, -43, and -46 are activated similar to tastants already presented. Additionally, TAS2R31 harboured one sensitive (TAS2R31-H2), two intermediates (-H1 and-H4), and one insensitive variants (-H3). Hence, several TAS2Rs contribute to phenotypic variation.

#### Absinthin

**Fig 8:**
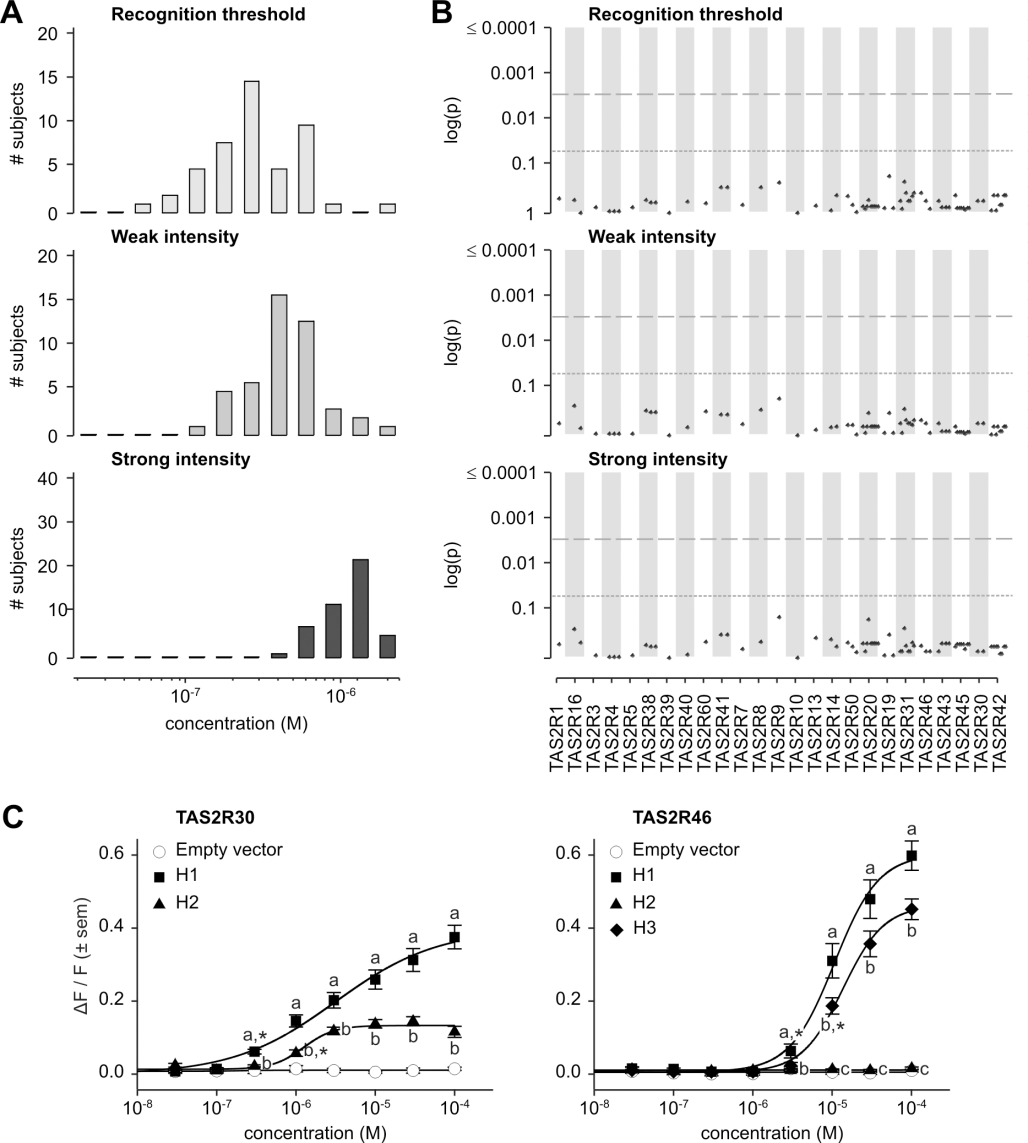
Absinthin phenotypes, genotype-phenotype associations, and functional assays. (A) Distribution of bitter taste recognition thresholds, and concentrations perceived as weak and strong. (B) Genotype-phenotype associations for common SNPs (frequency > 0.05). Dashed line indicates significance threshold following corrections for multiple testing. Solid line indicates uncorrected significance threshold. (C) Allele-specific dose response curves for loci harboring SNPs showing significant phenotypic associations.

The distribution of recognition thresholds for absinthin was broad, ranging 40-fold, from 5.2x10^-8^ M to 2.0x10^-6^ M (Fig 8A). Two modes were present, at 2.6x10^-7^ M and one at 5.9x10^-7^ M. The distribution of concentrations eliciting perceived weak intensity was narrower, ranging 15-fold, from 1.2x10^-7^ M to 2.0x10^-6^ M with a single mode at 3.9x10^-7^ M. The distribution of concentrations eliciting perceived strong intensity was yet narrower, ranging 5-fold, from 4.0x10^-7^ M to 2.0x10^-6^ M with a single mode at 1.3x10^-6^ M. Distributions of detection threshold and concentrations perceived as moderately and very strongly bitter exhibited largely analogous properties, with a detection threshold lower than recognition threshold, and means for perceived moderate, strong, and very strong intensities shifted gradually upward relative to perceived weak bitterness (S2 Fig).

While our previous inventory of receptor-agonist interactions identified TAS2R30 and -46 as responsive to absinthin, and both *TAS2R30* and -*46* harboured coding variations in our sample, association analyses found, however, no SNPs significantly associated with perception phenotypes (Figs 8B and S2; S2 Table).

Functional assays targeting TAS2R30 and -46 in our sample confirmed that both receptors are capable of responding to absinthin (Fig 8C). As noted earlier, TAS2R30 existed in two variants, TAS2R30-H1 and-H2. As with amarogentin and quassin, TAS2R30-H1 was strongly activated by absinthin (threshold = 3.0x10^-7^ M; EC_50_ = 3.1x10^-6^ M; maximum amplitude = 0.41 ΔF/F) while H2 was less sensitive, with a severely reduced maximal signal amplitude (threshold = 1.0x10^-6^ M; EC_50_ = 1.3x10^-6^ M; maximum amplitude = 0.15 ΔF/F). Likewise, three variants of TAS2R46 exhibited responses mirroring their responses to amarogentin and quassin. TAS2R46-H1 (threshold = 3.0x10^-6^ M; EC_50_ = 1.1x10^-5^ M; maximum amplitude = 0.63 ΔF/F) was more sensitive than-H3 (threshold = 1.0x10^-5^ M; EC_50_ = 1.4x10^-5^ M; maximum amplitude = 0.48 ΔF/F), and TAS2R46-H2 showed no response at any concentration.

The lack of association between absinthin perception and SNPs at *TAS2R30* and *-46*, in spite of the presence of functional diversity among these receptors, was explained by long-range haplotypes in the *TAS2R31-42* region (Fig 5). Two common long-range haplotypes coded the sensitive receptor variant TAS2R30-H1, combined with the non-functional TAS2R46-H2 or the moderately sensitive variant TAS2R46-H3, for the first (frequency = 0.30) and the third (frequency = 0.20) haplotype, respectively. The two other common long-range haplotypes, the second (frequency = 0.22) and the fourth (frequency = 0.16), coded the sensitive receptor variant TAS2R46-H1 and the insensitive TAS2R30-H2. Thus, while *TAS2R30* and *-46* both harboured alleles with variable sensitivity, such that their individual contributions to phenotype varied, their organization in long-range haplotypes resulted in 96% of subjects carrying exactly two copies of a high-sensitivity allele of at least one of the two genes, and 4% of subjects carrying one copy. This pattern explains the lack of genotypic association. This clarifies also the low concentration perceived as bitter by human subjects when compared with *in vitro* activation.

#### Cascarillin

Cascarillin elicited patterns of phenotypic and functional responses similar to those elicited by absinthin, but differing in important respects. As with absinthin, threshold recognition exhibited a broad distribution (17-fold, from 2.3x10^-6^ M to 4.0x10^-5^ M). Distribution of perceived weak intensity was narrower (11-fold, from 3.5x10^-6^ M to 4.0x10^-5^ M), followed by perceived strong intensity (S3 Fig). *In vitro*, the minimum concentration required to elicit response to cascarillin in functional assays was slightly higher (3.0x10^-5^ M). Indeed, receptor variants of TAS2R30 and -46 are activated in similar ways by absinthin and cascarillin, with TAS2R30 harbouring sensitive and insensitive variants (TAS2R30-H1 and-H2) and TAS2R46 harboring sensitive, intermediate, and insensitive variants (TAS2R46-H1,-H3, and-H2, respectively) (S3 Fig). Again, genotype-phenotype associations between variation at *TAS2R30* and *-46* failed to identify SNPs explaining phenotypic variation, likely as the result of linkage disequilibrium between responsive and nonresponse alleles of *TAS2R30* and *-46*, since all subjects carried at least one functional allele.

## Discussion

Bitter taste perception has long been known to have major heritable components. Common dominant and recessive alleles shaping sensitivity to specific chemical compounds were identified as early as the 1930s (for review see [47]). It is now known that much of this heritability is due to coding variation in *TAS2R* genes altering receptor affinity, which shapes perception phenotypes. In the classic case, patterns of bitter sensitivity are driven by strong, single-gene effects [17, 21, 25, 26]. However, such associations with only one *TAS2R* locus appear to be rare. Relationships between *TAS2R* variation and perception are likely most often complex due to high levels of genetic diversity, linkage between loci and overlap in receptor-agonist interactions, with some agonists stimulating multiple receptors and some receptors responding to multiple agonists [26, 31]. This hinders elucidation of bitter perception’s molecular underpinnings and, consequently, their downstream effects on aversion and ingestive behaviours [34]. Therefore, until now only a limited number of *TAS2R* genes have been implicated in the modification of ingestive behavior (for a review see [48]). Our findings reveal the extent to which linkage constrains both, variation in *TAS2R* genes and patterns of overlap in receptor-agonist affinity, and the impact of these factors on bitter taste phenotypes.

Human *TAS2R*s are highly diverse, with per nucleotide heterozygosity significantly higher than genome-wide averages, elevated rates of non-synonymous substitution, and fixation indices (F_ST_ values) indicative of substantial population differentiation [22]. This suggests that combinatorial variation across loci could, in principle, be extremely high. However, patterns of linkage disequilibrium in our sample of the Caucasian population demonstrate that such diversity is limited. While a total of 93 SNPs were present, which could in principle recombine to form more than 7 quintillion (7.71 x 10^30^) different combinations, most variation resided in just six blocks, each harbouring just 1 to 4 haplotypes. This finding has two implications. First, it suggests that while humans harbour 25 functional *TAS2R* loci, each of which encodes a receptor with alleles responsive to different ranges of agonists, phenotypic responses are likely correlated across compounds regardless of whether they are mediated by the same receptor. Second, linkage disequilibrium and block structures spanning loci are likely major sources of spurious genotype-phenotype associations. Because blocks often span several loci, yet few long-range haplotypes are present, true genotype-phenotype associations will most often be accompanied by false positives arising from sites in LD with the causal variants. This problem is compounded by the prevalence of non-synonymous variants in *TAS2R*s, which stand out as potential functional receptor candidates, making them difficult to rule out as causal. These issues were evident in our association analyses, which were able to localize signals for amarogentin, grosheimin, and quassin at the block level but not with respect to specific SNPs (Figs 7, 8 and S1–S3).

Functional assays characterizing the kinetic properties of individual TAS2R variants in our sample were successful in resolving the positions of sites shaping phenotypes, revealing complex variation in response across loci and alleles (Figs 6–8; S2 Table). Within each haplotype block implicated in associations, we found at least one locus harboring functionally polymorphic alleles corresponding to receptor variants activated in the concentration range perceived as bitter by subjects, thus pinpointing the sites underlying variable perception of four tastants. However, the mechanisms underlying variation in sensitivity varied from locus to locus. At TAS2R30, the high- and low-sensitivity receptor variants, which differed drastically in response to amarogentin and quassin, were distinguished by a single amino acid position (L252F) in the third extracellular domain of the receptor, which is hypothesized to interact directly with agonists. High- and low-sensitivity TAS2R46 variants also differed at a single amino acid position (L228M); however, this site, located in the sixth transmembrane domain of the receptor, is highly conserved among TAS2Rs and thought to be essential to the basic functionality of the receptor, which likely explains the strong effects of TAS2R46-L228M on phenotype. *TAS2R46* also harbored a common allele coding a premature stop codon (W250X), resulting in the production of a severely truncated, dysfunctional receptor [22]. At TAS2R43, the high- and low-sensitivity receptor variants differed at position W35S in the first intracellular domain and H212R in the fifth transmembrane domain; by considering the high degree of sequence similarity between TAS2R43 and TAS2R31, the sole presence of amino acid substitution W35S, corresponding to the deleterious substitution TAS2R31-R35W, likely explains the severe impaired functionality of the receptor [26]. Beyond these substitutions, *TAS2R43* harboured a high-frequency whole-gene deletion allele completely lacking an open reading frame, and unable to produce protein at all [25, 26].

In addition to identifying simple phenotypic associations with alleles at single loci, our analyses revealed associations arising from linkage disequilibrium across loci, demonstrating the complexity of relationships between *TAS2R* variation and phenotypic response. In the case of amarogentin and quassin, several loci (*TAS2R30*, *-43*, and *-46*) harboured both high- and low-sensitivity alleles, suggesting that loci could individually contribute up- or downward shifts in phenotype. However, only TAS2R30 harboured receptor variants responsive across the threshold ranges of subjects, indicating that it alone accounts for most variability in perception. This relationship is similar to long known patterns of bitter sensitivity driven by strong, single-gene effects. In the case of grosheimin, two loci (*TAS2R43* and *-46*) harboured both high- and low-sensitivity alleles. However, these were maintained in the same linkage phase such that the sensitive allele of *TAS2R43* was linked with the sensitive allele of *TAS2R46* and the insensitive allele of *TAS2R43* was linked with the insensitive allele of *TAS2R46*. Thus, while both loci harboured variation able to explain observed phenotypes, their contributions were strongly correlated. In the case of absinthin and cascarillin, again two loci (*TAS2R30* and *-46*) harboured both high- and low-sensitivity alleles. However, linkage disequilibrium maintained these in opposite phase such that the sensitive allele of *TAS2R30* was linked with the insensitive allele of *TAS2R46*, and the insensitive allele of *TAS2R30* was linked with the sensitive allele of *TAS2R46*. Thus, most subjects carried at least one allele sensitive to absinthin and cascarillin, explaining both the low mean threshold response to these compounds in subjects and the weak statistical associations for individual loci. These findings, together with prior evidence of extensive overlap in sensitivity across loci and agonists, suggest that while strong associations between a single *TAS2R* locus and phenotype may occur, they are likely uncommon. Moreover, linkage between *TAS2R* loci can cause confounds resulting in both false positive and false negative results in association analyses. Thus, dissecting genetic effects on bitter taste sensitivity through association analysis alone is likely to be inaccurate in most situations.

An essential aspect of *TAS2R* diversity in our sample of the Caucasian population, which has been broadly observed in population genetic studies, was that diversity is extremely high. In total we identified 93 SNPs, of which 67 SNPs were common, with frequencies above 5% (Fig 3). Moreover, every subject harboured a different allele combination at the SNP positions. Thus, inherited variation in taste responses was not a rarity as is the case for many phenotypes, such as diseases, but the norm. Further, our European sample, though ethnically homogeneous, captured most *TAS2R* variation found to date in worldwide populations [22, 40]. For example, beyond the 93 SNPs found in our subjects, Kim et al. (2005) reported only 10 additional common SNPs (5 synonymous and 5 non-synonymous) across a panel of 55 Africans, Asians, Europeans, and Native Americans. Hence, differences at *TAS2R* loci between individuals from ethnically diverse populations are modest in comparison to differences among individuals from the same population, consistent with more general observations on large-scale data sets [49, 50]. These patterns suggest that the association trends in our data are likely not restricted to Europeans, but relevant in most populations.

Yet, even if receptor polymorphism and genomic structure dominantly shape bitter taste perception, further studies need to be performed to enable a complete comprehension of variation in bitter taste perception, taking into account that other relevant factors may also play a significant role. Indeed, besides receptor-agonist interactions, differences in taste receptor gene expression levels could also contribute to individual differences in bitter taste perception. The importance of polymorphisms in the putative promoter regions of taste genes further indicates overlapping genetic influences on receptor expression and functionality [51, 52]. In addition, differences at peripheral level, e.g. in taste signaling cascade components [53], may also influence taste perception, as well as differences in signal transmission by afferent taste nerves and signal processing at central level. Hormones may also, at the level of the individual, impact bitter taste perception, e.g. hunger-satiety hormones as well as sex hormones (for review see [54, 55]).

Nonetheless, deciphering receptor activation patterns and linkage structure among TAS2R genes is an important prerequisite to establish a solid basis to assess bitter taste variations in the population. This may pave the way to evaluate the consequences of these variations in food rejection and ultimately help to improve public health.

## Material and Methods

### Ethics statement

This work was conducted in accordance to the Declaration of Helsinki on Biomedical Research Involving Human Subjects and approved by the Ethics Committee of the University of Potsdam (Germany) through decision 11 / 27. Session / 2009. All participants gave written informed consent.

### Subjects

The subject panel was composed of 48 unrelated Caucasian subjects (39 women, 9 men; age range 21 to 59 years, mean age 30.6 years), recruited at the German Institute of Human Nutrition Potsdam-Rehbruecke (Germany). All were pre-screened to avoid inclusion of individuals with health problems and overt taste pathologies; pregnant and breast-feeding women were also excluded. Each subject participated in the entire course of the study, which included one training session and nine experimental sessions. Visits consisted of DNA collection and psychophysical tests for genotyping and phenotypic analyses, respectively. General taste abilities were also assessed for the bitter, salty, sour, and sweet taste during the first session.

### Taste compounds

Six structurally diverse bitter substances, found at low concentrations in various beverages, and known to exhibit pharmacological properties at high concentrations, were used to probe phenotypic and molecular responses (S1 Fig). These included absinthin (a dimeric sesquiterpene lactone), amarogentin (a secoiridoid glycoside), cascarillin (a diterpene lactone), grosheimin (a sesquiterpene lactone), quassin (a triterpene lactone), and quinine (a quinoline alkaloid). Absinthin, cascarillin, and grosheimin were isolated from crude vegetable material as detailed in previous studies [31, 56]. Amarogentin and quassin were purchased from Chromadex Inc. (Irvine, CA, USA), quinine hydrochloride from Sigma-Aldrich Co. (St. Louis, MO, USA). Salicin, sodium chloride, citric acid, and sucrose (Sigma-Aldrich Co.) were used as reference tastants for assessing bitter, salty, sour, and sweet perception, respectively.

### Genotype analysis

Gene locations and coding sequences of the 25 *TAS2R* genes were obtained from genomic scaffolds 1103279188109, 1103279188381, 1103279188228, and 1103279188408 of the whole genome assembly released by the Venter Institute human reference genome as well as from all subjects in the present study [57] (Fig 1). Corresponding amino acid sequences were aligned according the modified version of the Feng-Doolittle progressive alignment algorithm (Align X, Vector NTI; Life Technologies, Carlsbad, CA, USA) [58]. Alignment was then manually adjusted on the basis of previous *in silico* and *in vitro* experiments, in order to conserve structural and functional key domains, e.g., transmembrane domains and the conserved glycosylation site [41, 42, 59]. A neighbour-joining tree with bootstrap values was then constructed from aligned sequences using Clustal X [60].

Genomic DNA was obtained from saliva samples collected using Oragene DNA self-collection kits (Oragene DNA; DNA Genotek Inc., Kanata, Canada), and purified using prepIT-L2P kits (prepIT-L2P; DNA Genotek Inc.). Complete nucleotide sequences of every *TAS2R* coding region were then obtained for all subjects. Locus-specific primers localized in the flanking regions of each gene, ~100 bp upstream of the start codon and ~100 bp downstream of the stop codon, were designed using the Primer-Blast tool or obtained from previous studies [22, 26, 40, 61]. Corresponding DNA fragments of at least ~1 kbp were amplified by PCR using a high-throughput polymerase (Advantage 2 polymerase mix; Takara Bio Inc., Otsu, Japan). Amplified DNA fragments were sequenced by capillary electrophoresis of both forward and reverse strands (Eurofins MWG Operon, Ebersberg, Germany). Reads were assembled and trimmed to remove low-quality sequence (Vector NTI; Life Technologies). All single-nucleotide polymorphisms (SNPs) were then identified and individual genotypes were determined. Departures from the Hardy-Weinberg equilibrium were tested to rule out genotyping problems.

### Copy number determination

Previously reported major deletions at *TAS2R43* and *-45* loci, ~39k b and ~32 kb in length, respectively, were characterised by multiplex PCR reactions. These were performed using primer sets targeted within, outside, and spanning the deleted regions such that amplifications produced alternate products in deleted and non-deleted alleles (S4 Fig). Gel separation and sequencing of the resulting fragments (Advantage 2 polymerase; Takara Bio Inc.) revealed whether a subject carried zero, one or two copies of each gene.

### Haplotype estimation

DNA sequence and copy-number data were jointly used to call genotypes. Haplotypes were then either directly ascertained for homozygous individuals or inferred using PHASE, which utilizes Bayesian algorithms to resolve haplotypes in heterozygous individuals [62, 63]. In cases of uncertain phase, haplotypes were confirmed by comparison with previously published data or identified by cDNA cloning and sequence analysis [26]. Linkage disequilibrium measures were then obtained by calculating D’, r^2^ and corresponding *p*-values between multi-allelic loci for both SNPs and genes [64, 65]. Haplotype block partitions were generated according to the four gamete rule with a 5% cut-off score, and manually adjusted for *TAS2R19* and *TAS2R42*, which SNPs spanned several blocks [66, 67]. Corresponding long-range haplotypes were then inferred [62, 63]. Algorithms for visual representation were specifically implemented in Matlab, based on the population genetics and evolution toolbox (Matlab; The MathWorks Inc., Natick, MA, USA) [68].

### Phenotype analysis

Experiments were conducted at the sensory analysis laboratory of the German Institute of Human Nutrition Potsdam-Rehbruecke, according to good practice guidelines [69, 70]. Detection and recognition thresholds were assessed using a procedure adapted from the norm ISO 13301:2002 of the International Organization for Standardization: a four-alternative ascending forced-choice procedure, followed by yes-no questions about the bitter taste quality of the quoted samples [71]. Perceived bitter taste intensities were rated on the general Labelled Magnitude Scale (gLMS; [72–74]). Concentration series consisted of geometric sequences of twelve steps, with a 1.5 common ratio and the following concentration ranges: 2.3x10^-8^–2.0x10^-6^M, 3.5x10^-9^–3.0x10^-7^ M, 4.6x10^-7^–4.0x10^-5^ M, 5.8x10^-7^–5.0x10^-5^ M, 1.2x10^-8^–1.0x10^-6^ M, 4.6x10^-7^–4.0x10^-5^ M, for absinthin, amarogentin, cascarillin, grosheimin, quassin, and quinine, respectively. For each 4-AFC test, four coded samples containing 10 ml solution were presented simultaneously; one containing the bitter tastant diluted in mineral water (Evian; Danone, Paris, France) and three containing mineral water only. At each concentration, subjects were challenged to identify the different sample, specify whether the quoted sample tasted bitter, and rate the perceived bitter intensity. Tests were performed with nose clips and oral rinsing, with a 45 s pause between concentrations. Training sessions were first used to familiarize subjects with the experimental procedures, and secondly, to assess general taste abilities. Concentration series consisted of geometric sequences of six steps, with a 1.5 common ratio and the following concentration ranges: 3.5x10^-5^–2.0x10^-3^M, 6.9x10^-4^–4.0x10^-2^ M, 2.6x10^-4^–1.5x10^-2^ M, 6.9x10^-4^–4.0x10^-2^ M, for salicin, sodium chloride, citric acid, and sucrose used as reference tastants for bitter, salty, sour, and sweet taste, respectively. Following training, subjects tested each tastant in triplicate over nine sessions. Latin square designs extended for first-order carry-over effects were used to counterbalance presentation order of the test compounds over the test sessions, across subjects and for each test compound, as well as presentation order of the samples at each concentration across both repetitions and subjects. Sensory sessions were monitored and data automatically collected (Fizz; Biosystèmes, Couternon, France).

Detection and recognition probabilities of the bitter samples were analysed per repetition and subject. Relationships between probabilities (detection or recognition probabilities) and concentrations were fitted by a logistic regression model using the maximum-likelihood method. Threshold value and slope of the logistic curves were then obtained per repetition and subject, using a self-implemented toolbox (Matlab; The MathWorks Inc.). No repetition effect was observed. Where required, outliers were discarded according to the Peirce’s criterion [75, 76]. Perceived intensities, expressed in percentage of the scale length, were analysed similarly. Concentrations were subsequently inferred for weak, moderate, strong, and very strong intensities, corresponding respectively to 6, 17, 34.7, and 52.5% of the scale length.

### Genotype-phenotype association analyses

General linear mixed model analyses were performed at SNP, gene, and LD block level (SAS Institute Inc., Cary, NC, USA). Concentrations corresponding to detection thresholds, recognition thresholds, or perceived intensities were treated as dependent variables following a log-normal distribution. Genotype or haplotype with frequencies above 0.05 were used as independent variables and subjects as random variables. For analyses at the SNP level, probability values were assessed at an uncorrected significance level of 0.05, and at an experiment-wide significance threshold required to keep a significance level of 0.05, thus correcting for multiple comparisons [77]. For analyses at gene-specific level and LD block level, a Bonferroni correction for multiple corrections was applied.

### Functional analyses of TAS2R variants

Allelic responses to agonists were quantified using *in vitro* heterologous expression assays designed in previous studies of TAS2R receptor function, which successfully mimic responses *in vivo* with respect to both magnitude and concentration range [17, 23, 25, 26, 46]. DNA fragments containing *TAS2R* coding sequences were first amplified from genomic DNA by PCR using a proofreading polymerase (PfuUltra II Fusion HotStart DNA Polymerase; Agilent Technologies, Santa Clara, CA, USA) and cloned into a plasmid vector according to the manufacturer’s protocol (Zero Blunt TOPO PCR Cloning Kit; Life Technologies). A second PCR was performed using specific cloning primers to facilitate subcloning into the expression vector, which was then modified to add an N-terminal signal for cell surface localisation and a C-terminal epitope for immunocytochemical detection to the receptor sequence (Fast Link DNA Ligation Kit; Epicentre, Illumina Inc., Madison, WI, USA) (pcDNA5/FRT Mammalian Expression Vector; Life Technologies). Empty vector was used as a negative control [23, 31].

Functional assays were carried out in HEK 293T cells stably expressing the G protein chimera Gα16gust44 and transiently transfected with *TAS2R* alleles subcloned into the expression vector [78]. Calcium imaging was performed using an automated fluorometric imaging plate reader by exposing transfected cells to test compounds dissolved in assay buffer or to assay buffer alone (FLIPR Tetra; Molecular Devices, LLC, Sunnyvale, CA, USA). Changes in cytosolic calcium levels were monitored by measuring fluorescence intensity of a calcium-sensitive dye previously added (Fluo-4 AM; Life Technologies). Six replicates were carried out for each bitter stimulus, on separate experimental days, with each replicate consisting of concentration series of each bitter tastant.

Prior to final analysis, fluorescence data from functional assays, expressed in relative fluorescence units (RFU), underwent three corrections. First, a correction calculated from baseline values was applied to compensate for well-to-well fluctuations. A second correction calculated from negative control values was applied to correct for receptor independent artefacts and signal drift. A third correction calculated from positive control values was applied to facilitate comparison of data obtained from different experimental days. Fluorescence ratios ((F-F_0_)/F_0_), obtained by subtracting the background fluorescence from fluorescence peak height and then dividing the difference by the background fluorescence, were then used for data analysis. Variance analyses were performed, followed by Bonferroni multiple comparisons tests (SPSS 20; IBM Corporation, Armonk, NY, USA). Threshold response values were then defined as the first concentration eliciting a significant activation of the receptor, with empty vector acting as a negative control. Finally, dose-response curves were fitted to the Hill equation by nonlinear regression in order to determine half maximal effective concentrations (EC_50_) and maximal amplitude [79] (SigmaPlot; Systat Software Inc., Chicago, IL, USA).

## Supporting Information

S1 Table Coding SNPs and haplotypes of *TAS2R* genes. Haplotypes are detailed for each gene, along with each variable codon and its alternative nucleotide and encoded amino acids. Allele and carrier frequencies for each haplotype are detailed, as well as minor allele frequency, heterozygosity, and Hardy-Weinberg p-value for each SNP. Haplotypes were either (1) directly ascertained for homozygous individuals or (2) inferred using a Bayesian approach for heterozygous individuals [62, 63]. In case of uncertain haplotype phases, haplotypes were confirmed (3) by comparison with previously published data [26] or identified by cDNA cloning and sequence analysis. Haplotypes were named (6) according to previous nomenclature [26] or according to their respective allele frequencies in our sample of the European population; the most common haplotypes receiving the smallest number. Previously published protein-coding haplotypes (4) [31] or receptor variants obtained through single point mutagenesis (5) [39] were also detailed. Haplotypes were named according to previous nomenclature or according to their respective allele frequencies in our subjects; the most common haplotypes receiving the smallest number [26].
(PDF)

S2 Table Probability values of the genotype-phenotype associations determined for the bitter substances absinthin, amarogentin, cascarillin, grosheimin, quassin, and quinine. Probability values are detailed per receptor for each common SNPs. Probability values, which reached significance level of 0.05, are underlined in grey; probability values, which reached the experiment-wide significance threshold, appear in bold type.
(PDF)

S1 Fig Structural formulas of bitter tastants. Formulas are detailed for absinthin, amarogentin, cascarillin, grosheimin, quassin, and quinine.
(PDF)

S2 Fig Phenotype and genotype-phenotype associations determined for the bitter substances absinthin, amarogentin, cascarillin, grosheimin, quassin, and quinine. Distribution of the subjects is plotted for detection and recognition thresholds, as well as for concentrations corresponding to weak, moderate, strong, and very strong bitter taste intensities (left panel). Identically, genotype-phenotype associations for each common SNP are specified for detection and recognition thresholds, as well as for concentrations corresponding to weak, moderate, strong, and very strong bitter taste intensities (right panel). Significance level of 0.05 (dotted line), or at the experiment-wide significance threshold required to keep a significance level of 0.05 (dashed line) are specified.
(PDF)

S3 Fig Phenotype, genotype-phenotype associations, and functional assays determined for the bitter substances cascarillin, quassin, and quinine. (A) Distribution of the subjects is plotted for recognition thresholds, as well as for concentrations corresponding to weak and strong bitter taste intensities. (B) Identically, genotype-phenotype associations for each common SNP are specified for recognition thresholds, as well as for concentrations corresponding to weak and strong bitter taste intensities. Significance level of 0.05 (dotted line), or at the experiment-wide significance threshold required to keep a significance level of 0.05 (dashed line) are specified. (C) Variants of TAS2R candidates were functionally challenged in heterologous cell-based assays.
(PDF)

S4 Fig Copy-number variations at the *TAS2R43* and -*45* loci. Major overlapping deletions of ~39kb and ~32kb in length were identified at the *TAS2R43* locus and at the *TAS2R45* locus, respectively. Consistent with these findings, PCR with primers in the flanking regions of *TAS2R43* (PCR1) or *TAS2R45* (PCR5) failed. Multiplex PCR distinguished subjects with zero, one or two copies of each gene. The sole presence of DNA fragments spanning a deletion indicates a copy number zero (PCR4 at *TAS2R43* locus; PCR8 at *TAS2R45* locus), whereas the sole presence of DNA fragments obtained with primers located within a potentially deleted DNA region indicates a copy number of two (PCR2 and PCR3 at *TAS2R43* locus; PCR6 and PCR7 at *TAS2R45* locus). The presence of both kinds of DNA fragments indicates a copy number one.
(PDF)
