# Supplementary material for: Receptor Polymorphism and Genomic Structure Interact to Shape Bitter Taste Perception
Source: PLoS Genet. 2015 Sep 25;11(9):e1005530. doi: 10.1371/journal.pgen.1005530 (PMC4583475; doi:10.1371/journal.pgen.1005530)
Supplement: S1 Table — Haplotypes are detailed for each gene, along with each variable codon and its alternative nucleotide and encoded amino acids. Allele and carrier frequencies for each haplotype are detailed, as well as minor allele frequency, heterozygosity, and Hardy-Weinberg p-value for each SNP. Haplotypes were either (1) directly ascertained for homozygous individuals or (2) inferred using a Bayesian approach for heterozygous individuals [62, 63]. In case of uncertain haplotype phases, haplotypes were confirmed (3) by comparison with previously published data [26] or identified by cDNA cloning and sequence analysis. Haplotypes were named (6) according to previous nomenclature [26] or according to their respective allele frequencies in our sample of the European population; the most common haplotypes receiving the smallest number. Previously published protein-coding haplotypes (4) [31] or receptor variants obtained through single point mutagenesis (5) [39] were also detailed. Haplotypes were named according to previous nomenclature or according to their respective allele frequencies in our subjects; the most common haplotypes receiving the smallest number [26]. (PDF) [file pgen.1005530.s001.pdf]

TAS2R1 (Gene ID: 50834, Transcript ID: NM_019599.2, Protein ID: NP_062545.1)

	G 332 A<br>R 111 H<br>rs41469	C 616 T<br>R 206 W<br>rs2234233	T 850 C<br>L 284 L<br>rs2234235	Allele freq.<br>n=96	Homoz.	Heteroz.	Carrier freq.<br>n=48
H1 [1,2,3,4]	CGT<br>R	CGG<br>R	TTG<br>L	0.76	0.58	0.35	0.94
H2 [1,2,3]	CGT<br>R	TGG<br>W	TTG<br>L	0.20	0.04	0.31	0.35
H3 [3]	CGT<br>R	CGG<br>R	CTG<br>L	0.03	-	0.06	0.06
H4 [2,3]	CAT<br>H	CGG<br>R	TTG<br>L	0.01	-	0.02	0.02
Minor allele freq.	0.01	0.20	0.03				
Heteroz.	0.02	0.31	0.06				
Hardy-Weinberg p-value	0.94	0.91	0.82				

TAS2R16 (Gene ID: 50833, Transcript ID: NM_016945.2, Protein ID: NP_058641.1)

	C 475 G<br>H 159 D	G 663 A<br>A 221 A<br>rs150899693	G 665 A<br>R 222 H<br>rs860170	G 846 A<br>T 282 T<br>rs1204014	Allele freq.<br>n=96	Homoz.	Heteroz.	Carrier freq.<br>n=48
H1 [1,2]	CAT<br>H	GCG<br>A	CAC<br>H	ACG<br>T	0.59	0.35	0.48	0.83
H2 [1,2,3,4]	CAT<br>H	GCG<br>A	CGC<br>R	ACG<br>T	0.33	0.08	0.50	0.58
H3 [3]	CAT<br>H	GCG<br>A	CAC<br>H	ACA<br>T	0.05	-	0.10	0.10
H4 [3]	CAT<br>H	GCA<br>A	CAC<br>H	ACG<br>T	0.01	-	0.02	0.02
H5 [2,3]	GAT<br>D	GCG<br>A	CAC<br>H	ACG<br>T	0.01	-	0.02	0.02
Minor allele freq.	0.01	0.01	0.33	0.05				
Heteroz.	0.02	0.02	0.50	0.10				
Hardy-Weinberg p-value	0.94	0.94	0.39	0.70				

TAS2R3 (Gene ID: 50831, Transcript ID: NM_016943.2, Protein ID: NP_058639.1)

	C 807 T<br>G 269 G<br>rs2270009	Allele freq.<br>n=96	Homoz.	Heteroz.	Carrier freq.<br>n=48
H1 [1,2,4]	GGC<br>G	0.57	0.38	0.40	0.77
H2 [1,2]	GGT<br>G	0.43	0.23	0.40	0.63
Minor allele freq.	0.43				
Heteroz.	0.40				
Hardy-Weinberg p-value	0.19				

TAS2R4 (Gene ID: 50832, Transcript ID: NM_016944.1, Protein ID: NP_058640.1)

	T 20 C<br>F 7 S<br>rs2233998	A 180 G<br>G 60 G<br>rs144806722	G 286 C<br>V 96 L<br>rs2234001	G 512 A<br>S 171 N<br>rs2234002	Allele freq.<br>n=96	Homoz.	Heteroz.	Carrier freq.<br>n=48
H1 [1,2,3,4]	TTC<br>F	GGA<br>G	GTC<br>V	AGT<br>S	0.55	0.35	0.40	0.75
H2 [1,2]	TCC<br>S	GGA<br>G	CTC<br>L	AAT<br>N	0.44	0.25	0.38	0.63
H3 [3]	TCC<br>S	GGG<br>G	CTC<br>L	AAT<br>N	0.01	-	0.02	0.02
Minor allele freq.	0.45	0.01	0.45	0.45				
Heteroz.	0.40	0.02	0.40	0.40				
Hardy-Weinberg p-value	0.17	0.94	0.17	0.17				

TAS2R5 (Gene ID: 54429, Transcript ID: NM_018980.2, Protein ID: NP_061853.1)

	G 77 T<br>S 26 I<br>rs2227264	Allele freq.<br>n=96	Homoz.	Heteroz.	Carrier freq.<br>n=48
H1 [1,2,4]	AGC<br>S	0.57	0.38	0.40	0.77
H2 [1,2]	ATC<br>I	0.43	0.23	0.40	0.63
Minor allele freq.	0.43				
Heteroz.	0.40				
Hardy-Weinberg p-value	0.19				

TAS2R38 (Gene ID: 5726, Transcript ID: NM_176817.4, Protein ID: NP_789787.4)

	G 145 C<br>A 49 P<br>rs713598	T 785 C<br>V 262 A<br>rs1726866	A 886 G<br>I 296 V<br>rs10246939	Allele freq.<br>n=96	Homoz.	Heteroz.	Carrier freq.<br>n=48
H1 [1,2]	GCA<br>A	GTT<br>V	ATC<br>I	0.57	0.35	0.44	0.79
H2 [1,2,4]	CCA<br>P	GCT<br>A	GTC<br>V	0.41	0.21	0.40	0.60
H3 [2]	GCA<br>A	GCT<br>A	GTC<br>V	0.02	-	0.04	0.04
Minor allele freq.	0.41	0.43	0.43				
Heteroz.	0.40	0.44	0.44				
Hardy-Weinberg p-value	0.21	0.46	0.46				

TAS2R39 (Gene ID: 259285, Transcript ID: NM_176881.2, Protein ID: NP_795362.2)

	-<br>-	Allele freq.<br>n=96	Homoz.	Heteroz.	Carrier freq.<br>n=48
H1 [1,2,4]	-<br>-	1.00	1.00	-	1.00
Minor allele freq.	-				
Heteroz.	-				
Hardy-Weinberg p-value	-				

TAS2R40 (Gene ID: 259286, Transcript ID: NM_176882.1, Protein ID: NP_795363.1)

	C 560 A<br>S 187 Y<br>rs10260248	Allele freq.<br>n=96	Homoz.	Heteroz.	Carrier freq.<br>n=48
H1 [1,2,4]	TCT<br>S	0.94	0.88	0.13	1.00
H2 [2]	TAT<br>Y	0.06	-	0.13	0.13
Minor allele freq.	0.06				
Heteroz.	0.13				
Hardy-Weinberg p-value	0.64				

TAS2R60 (Gene ID: 338398, Transcript ID: NM_0177437.1 , Protein ID: NP_803186.1)

		G 130 A<br>V 44 M<br>rs61750008	TCT 386--388 δ<br>VF 130--131 V<br>rs78798389	T 930 C<br>R 310 R<br>rs4595035	Allele freq.<br>n=96	Homoz.	Heteroz.	Carrier freq.<br>n=48
H1 [1,2,3,4]	RV1 [5]	GTG<br>V	TCT<br>VF	CGC<br>R	0.70	0.46	0.48	0.94
H2 [1,2,3]	RV1 [5]	GTG<br>V	TCT<br>VF	CGT<br>R	0.29	0.04	0.50	0.54
H3 [2,3]		ATG<br>M	δ<br>V	CGT<br>R	0.01	-	0.02	0.02
Minor allele freq.		0.01	0.01	0.30				
Heteroz.		0.02	0.02	0.48				
Hardy-Weinberg p-value		0.94	0.94	0.34				

TAS2R41 (Gene ID: 259287, Transcript ID: NM_176883.2, Protein ID: NP_795364.2)

		G 189 A<br>T 63 T<br>rs1404635	C 380 T<br>P 127 L<br>rs10278721	C 643 A<br>Q 215 K	G 885 A<br>S 295 S<br>rs145885431	Allele freq.<br>n=96	Homoz.	Heteroz.	Carrier freq.<br>n=48
H1 [1,2,3]	RV1 [5]	ACG<br>T	CCA<br>P	CAG<br>Q	TCG<br>S	0.69	0.42	0.54	0.96
H2 [1,2,4]	RV2 [5]	ACA<br>T	CTA<br>L	CAG<br>Q	TCG<br>S	0.29	0.04	0.50	0.54
H3 [2,3]		ACG<br>T	CCA<br>P	AAG<br>K	TCG<br>S	0.01	-	0.02	0.02
H4 [2,3]		ACA<br>T	CTA<br>L	CAG<br>Q	TCA<br>S	0.01	-	0.02	0.02
Minor allele freq.		0.30	0.30	0.01	0.01				
Heteroz.		0.52	0.52	0.02	0.02				
Hardy-Weinberg p-value		0.10	0.10	0.94	0.94				

TAS2R7 (Gene ID: 50837 , Transcript ID: NM_023919.2, Protein ID: NP_76408.1)

	T 254 C<br>I 85 T<br>rs77050900	G 538 T<br>A 180 S<br>rs76112700	A 787 T<br>T 263 S<br>rs11838055	G 912 A<br>M 304 I<br>rs619381	Allele freq.<br>n=96	Homoz.	Heteroz.	Carrier freq.<br>n=48
H1 [1,2,3,4]	ATT<br>I	GCT<br>A	ACG<br>T	ATG<br>M	0.85	0.73	0.25	0.98
H2 [1,2]	ATT<br>I	GCT<br>A	ACG<br>T	ATA<br>I	0.14	0.02	0.23	0.25
H3 [3]	ACT<br>T	TCT<br>S	TCG<br>S	ATG<br>M	0.01	-	0.02	0.02
Minor allele freq.	0.01	0.01	0.01	0.14				
Heteroz.	0.02	0.02	0.02	0.23				
Hardy-Weinberg p-value	0.94	0.94	0.94	0.88				

TAS2R8 (Gene ID: 50836, Transcript ID: NM_023918.1, Protein ID: NP_076407.1)

	C 142 T<br>L 48 F<br>rs74611066	G 164 T<br>R 55 I<br>rs142540719	G 549 A<br>L 183 L<br>rs1548803	A 684 C<br>E 228 D<br>rs143804727	Allele freq.<br>n=96	Homoz.	Heteroz.	Carrier freq.<br>n=48
H1 [1,2,3,4]	CTT<br>L	AGA<br>R	TTA<br>L	GAA<br>E	0.55	0.25	0.60	0.85
H2 [1,2]	CTT<br>L	AGA<br>R	TTG<br>L	GAA<br>E	0.42	0.13	0.58	0.71
H3 [2]	CTT<br>L	AGA<br>R	TTG<br>L	GAC<br>D	0.01	-	0.02	0.02
H4 [3]	CTT<br>L	ATA<br>I	TTG<br>L	GAA<br>E	0.01	-	0.02	0.02
H5 [3]	TTT<br>F	AGA<br>R	TTG<br>L	GAA<br>E	0.01	-	0.02	0.02
Minor allele freq.	0.01	0.01	0.45	0.01				
Heteroz.	0.02	0.02	0.60	0.02				
Hardy-Weinberg p-value	0.94	0.94	0.12	0.94				

TAS2R9 (Gene ID: 50835 , Transcript ID: NM_023917.2, Protein ID: NP_076406.1)

	A 535 G<br>K 179 E<br>rs149488505	T 560 C<br>V 187 A<br>rs3741845	G 630 A<br>K 210 K	G 697 A<br>A 233 T<br>rs77609577	Allele freq.<br>n=96	Homoz.	Heteroz.	Carrier freq.<br>n=48
H1 [1,2,3]	AAA<br>K	GTG<br>V	AAG<br>K	GCA<br>A	0.52	0.29	0.46	0.75
H2 [1,2,3,4]	AAA<br>K	GCG<br>A	AAG<br>K	GCA<br>A	0.46	0.23	0.46	0.69
H3 [3]	AAA<br>K	GCG<br>A	AAA<br>K	GCA<br>A	0.01	-	0.02	0.02
H4 [3]	GAA<br>E	GCG<br>A	AAG<br>K	ACA<br>T	0.01	-	0.02	0.02
Minor allele freq.	0.01	0.48	0.01	0.01				
Heteroz.	0.02	0.46	0.02	0.02				
Hardy-Weinberg p-value	0.94	0.57	0.94	0.94				

TAS2R10 (Gene ID: 50839, Transcript ID: NM_023921.1, Protein ID: NP_076410.1)

	-<br>-	Allele freq.<br>n=96	Homoz.	Heteroz.	Carrier freq.<br>n=48
H1 [1,2]	-<br>-	1.00	1.00	-	1.00
Minor allele freq.	-				
Heteroz.	-				
Hardy-Weinberg p-value	-				

TAS2R13 (Gene ID: 50838, Transcript ID: NM_023920.2, Protein ID: NP_76409.1)

	A 586 G<br>I 196 V	A 776 G<br>N 259 S<br>rs1015443	Allele freq.<br>n=96	Homoz.	Heteroz.	Carrier freq.<br>n=48
H1 [1,2,3]	ATC	AAC	0.52	0.25	0.54	0.79
	I	N				
H2 [1,2,4]	ATC	AGC	0.47	0.21	0.52	0.73
	I	S				
H3 [3]	GTC	AGC	0.01	-	0.02	0.02
	V	S				
Minor allele freq.	0.01	0.48				
Heteroz.	0.02	0.54				
Hardy-Weinberg p-value	0.94	0.55				

TAS2R14 (Gene ID: 50840 , Transcript ID: NM_023922.1, Protein ID: NP_076411.1)

	A 114 T<br>G 38 G<br>rs7138535	G 375 A<br>R 125 R<br>rs3741843	C 601 T<br>L 201 F<br>rs35804287	C 609 T<br>F 203 F<br>rs117092928	Allele freq.<br>n=96	Homoz.	Heteroz.	Carrier freq.<br>n=48
H1 [1,2,4]	GGA	AGA	CTC	TTC	0.48	0.23	0.50	0.73
	G	R	L	F				
H2 [1,2,3]	GGT	AGA	CTC	TTC	0.34	0.10	0.48	0.58
	G	R	L	F				
H3 [2,3]	GGA	AGG	CTC	TTC	0.16	-	0.31	0.31
	G	R	L	F				
H4 [2,3]	GGA	AGG	CTC	TTT	0.01	-	0.02	0.02
	G	R	L	F				
H5 [3]	GGA	AGA	TTC	TTC	0.01	-	0.02	0.02
	G	R	F	F				
Minor allele freq.	0.35	0.17	0.01	0.01				
Heteroz.	0.50	0.29	0.02	0.02				
Hardy-Weinberg p-value	0.52	0.73	0.94	0.94				

TAS2R50 (Gene ID: 259296, Transcript ID: NM_176890.2, Protein ID: NP_795371.2)

	A 525 G<br>S 175 S<br>rs66679979	G 608 A<br>C 203 Y<br>rs1376251	G 777 A<br>P 259 P<br>rs10772397	Allele freq.<br>n=96	Homoz.	Heteroz.	Carrier freq.<br>n=48
H1 [1,2]	TCA<br>S	TGT<br>C	CCG<br>P	0.31	0.06	0.50	0.56
H2 [1,2,4]	TCA<br>S	TAT<br>Y	CCA<br>P	0.28	0.02	0.52	0.54
H3 [1,2]	TCA<br>S	TGT<br>C	CCA<br>P	0.25	0.04	0.42	0.46
H4 [1,2]	TCG<br>S	TGT<br>C	CCG<br>P	0.16	0.02	0.27	0.29
Minor allele freq.	0.16	0.28	0.47				
Heteroz.	0.27	0.52	0.56				
Hardy-Weinberg p-value	0.85	0.05	0.37				

TAS2R20 (Gene ID: 259295, Transcript ID: NM_176889.2, Protein ID: NP_795370.2)

	T 38 C<br>V 13 A<br>rs141300962	A 156 G<br>A 52 A<br>rs11054143	A 235 G<br>K 79 E<br>rs7135018	C 261 T<br>A 87 A<br>rs11054142	G 421 A<br>V 141 I<br>rs79420812	C 429 A<br>H 143 Q<br>rs12226920	C 442 A<br>H 148 N<br>rs12226919	A 706 G<br>I 236 V<br>rs10845281	T 755 C<br>F 252 S<br>rs10845280	G 764 T<br>R 255 L<br>rs10845279	G 786 A<br>M 262 I<br>rs141300962	Allele freq.<br>n=96	Homoz.	Heteroz.	Carrier freq.<br>n=48
H1 [1,2]	GTA<br>V	GCA<br>A	AAA<br>K	GCC<br>A	GTT<br>V	CAC<br>H	CAC<br>H	ATA<br>I	TTT<br>F	CGA<br>R	ATG<br>M	0.40	0.10	0.58	0.69
H2 [1,2,3,4]	GTA<br>V	GCA<br>A	GAA<br>E	GCC<br>A	GTT<br>V	CAC<br>H	CAC<br>H	ATA<br>I	TTT<br>F	CGA<br>R	ATG<br>M	0.32	0.08	0.48	0.56
H3 [1,2,3]	GTA<br>V	GCG<br>A	AAA<br>K	GCT<br>A	ATT<br>I	CAA<br>Q	AAC<br>N	GTA<br>V	TCT<br>S	CTA<br>L	ATG<br>M	0.17	0.02	0.29	0.31
H4 [2]	GTA<br>V	GCG<br>A	AAA<br>K	GCT<br>A	GTT<br>V	CAA<br>Q	AAC<br>N	GTA<br>V	TCT<br>S	CTA<br>L	ATG<br>M	0.09	-	0.19	0.19
H5 [3]	GCA<br>A	GCG<br>A	AAA<br>K	GCT<br>A	GTT<br>V	CAA<br>Q	AAC<br>N	GTA<br>V	TCT<br>S	CTA<br>L	ATG<br>M	0.01	-	0.02	0.02
H6 [3]	GTA<br>V	GCA<br>A	AAA<br>K	GCC<br>A	GTT<br>V	CAC<br>H	CAC<br>H	ATA<br>I	TTT<br>F	CGA<br>R	ATA<br>I	0.01	-	0.02	0.02
Minor allele freq.	0.01	0.27	0.32	0.27	0.17	0.27	0.27	0.27	0.27	0.27	0.01				
Heteroz.	0.02	0.50	0.48	0.50	0.29	0.50	0.50	0.50	0.50	0.50	0.02				
Hardy-Weinberg p-value	0.94	0.07	0.51	0.07	0.73	0.07	0.07	0.07	0.07	0.07	0.94				

TAS2R19 (Gene ID: 259294 , Transcript ID: NM_176888.1, Protein ID: NP_795369.1)

		C 84 T<br>A 28 A<br>rs12313469	A 376 C<br>K 126 Q<br>rs12424373	T 418 C<br>L 140 L<br>rs1868769	C 895 T<br>R 299 C<br>rs10772420	Allele freq.<br>n=96	Homoz.	Heteroz.	Carrier freq.<br>n=48
H1 [1,2,3]	RV2 [5]	GCT<br>A	AAG<br>K	TTG<br>L	TGC<br>C	0.56	0.29	0.54	0.83
H2 [1,2,4]	RV1 [5]	GCC<br>A	AAG<br>K	TTG<br>L	CGC<br>R	0.26	0.02	0.48	0.50
H3 [1,2]	RV1 [5]	GCC<br>A	AAG<br>K	CTG<br>L	CGC<br>R	0.17	0.04	0.25	0.29
H4 [3]		GCC<br>A	CAG<br>Q	TTG<br>L	CGC<br>R	0.01	-	0.02	0.02
Minor allele freq.		0.44	0.01	0.17	0.44				
Heteroz.		0.54	0.02	0.25	0.54				
Hardy-Weinberg p-value		0.49	0.94	0.49	0.49				

TAS2R31 (Gene ID: 259290, Transcript ID: NM_176885.2, Protein ID: NP_795366.2)

	C 103 T<br>R 35 W<br>rs10845295	T 423 C<br>A 141 A<br>rs12370363	T 484 G/A<br>L 162 V/M<br>rs10743938	C 649 G<br>Q 217 E<br>rs10845294	C 680 T<br>A 227 V<br>rs10845293	G 718 A<br>V 240 I<br>rs10772423	A 744 G<br>S 248 S<br>rs116737741	C 827 G<br>P 276 R<br>rs12318612	Allele freq.<br>n=96	Homoz.	Heteroz.	Carrier freq.<br>n=48
H1 [1,2,4,6]	CGG<br>R	GCT<br>A	ATG<br>M	CAA<br>Q	GCT<br>A	GTT<br>V	TCA<br>S	CCA<br>P	0.25	0.02	0.46	0.48
H2 [1,2,3,6]	TGG<br>W	GCC<br>A	ATG<br>M	CAA<br>Q	GTT<br>V	ATT<br>I	TCA<br>S	CCA<br>P	0.32	0.08	0.48	0.56
H3 [1,2,3,6]	TGG<br>W	GCT<br>A	ATG<br>M	GAA<br>E	GTT<br>V	ATT<br>I	TCA<br>S	CGA<br>R	0.24	0.04	0.40	0.44
H4 [1,2,6]	CGG<br>R	GCT<br>A	TTG<br>L	CAA<br>Q	GCT<br>A	GTT<br>V	TCG<br>S	CCA<br>P	0.16	0.02	0.27	0.29
H5 [2,6]	CGG<br>R	GCT<br>A	TTG<br>L	CAA<br>Q	GCT<br>A	GTT<br>V	TCA<br>S	CCA<br>P	0.02	-	0.04	0.04
H8 [3]	CGG<br>R	GCT<br>A	GTG<br>V	CAA<br>Q	GCT<br>A	GTT<br>V	TCA<br>S	CCA<br>P	0.01	-	0.02	0.02
Minor allele freq.	0.44	0.32	0.18	0.24	0.44	0.44	0.16	0.24				
Heteroz.	0.50	0.48	0.27	0.40	0.50	0.50	0.27	0.40				
Hardy-Weinberg p-value	0.91	0.51	0.64	0.55	0.91	0.91	0.85	0.55				

TAS2R46 (Gene ID: 259292, Transcript ID: NM_176887.2, Protein ID: NP_795368.2)

	G 534 A<br>T 178 T<br>rs73260771	T 682 A<br>L 228 M<br>rs2708380	G 749 A<br>W 250 X<br>rs2708381	Allele freq.<br>n=96	Homoz.	Heteroz.	Carrier freq.<br>n=48
H1 [1,2,4,6]	ACG<br>T	TTG<br>L	TGG<br>W	0.44	0.19	0.50	0.69
H2 [1,2,6]	ACG<br>T	ATG<br>M	TAG<br>X	0.32	0.08	0.48	0.56
H3 [1,2,6]	ACA<br>T	ATG<br>M	TGG<br>W	0.24	0.04	0.40	0.44

Minor allele freq.	0.24	0.44	0.32
Heteroz.	0.40	0.50	0.48
Hardy-Weinberg p-value	0.55	0.91	0.51

TAS2R43 (Gene ID: 259289, Transcript ID: NM_176884.2, Protein ID: NP_795365.2)

	G 104 C<br>W 35 S<br>rs68157013	G 391 A<br>V 131 M<br>rs186718859	A 635 G<br>H 212 R<br>rs71443637	C 663 G<br>T 221 T<br>rs35720106	Allele freq.<br>n=96	Homoz.	Heteroz.	Carrier freq.<br>n=48
Δ [1,2,6]	Δ<br>Δ	Δ<br>Δ	Δ<br>Δ	Δ<br>Δ	0.56	0.31	0.50	0.81
H1 [1,2,4,6]	TCG<br>S	GTG<br>V	CGT<br>R	ACG<br>T	0.26	0.02	0.48	0.50
H2 [1,2,6]	TGG<br>W	GTG<br>V	CAT<br>H	ACC<br>T	0.17	0.02	0.29	0.31
H4 [2,6]	TCG<br>S	ATG<br>M	CGT<br>R	ACG<br>T	0.01	-	0.02	0.02

Minor allele freq.	0.17	0.01	0.17	0.17
Heteroz.	0.15	-	0.15	0.15
Hardy-Weinberg p-value	0.06	0.51	0.06	0.06

TAS2R45 (Gene ID: 259291, Transcript ID: NM_176886.2, Protein ID: NP_795367.2)

		A 227 G<br>Y 76 C<br>rs11535673	G 394 A<br>V 132 M<br>rs11526470	G 630 C<br>Q 210 H<br>rs28581524	T 703 C<br>F 235 L<br>rs3759244	C 712 T<br>R 238 C<br>rs3759245	G 893 C<br>R 298 T<br>rs11537119	A 900 G<br>X 300 W<br>rs3759247	Allele freq.<br>n=96	Homoz.	Heteroz.	Carrier freq.<br>n=48
Δ [1,2,6]		Δ	Δ	Δ	Δ	Δ	Δ	Δ	0.17	0.02	0.29	0.31
		Δ	Δ	Δ	Δ	Δ	Δ	Δ				
H1 [1,2,6]	RV2 [5]	TAT	GTG	CAG	TTC	CGT	AGG	TGA	0.36	0.15	0.44	0.58
		Y	V	Q	F	R	R	X				
H2 [1,2,6]	RV1 [5]	TGT	ATG	CAC	CTC	TGT	ACG	TGG	0.25	0.02	0.46	0.48
		C	M	H	L	C	T	W				
H3 [1,2,6]	RV3 [5]	TAT	GTG	CAG	TTC	TGT	AGG	TGA	0.22	0.04	0.35	0.40
		Y	V	Q	F	C	R	X				
Minor allele freq.		0.25	0.25	0.25	0.25	0.36	0.25	0.25				
Heteroz.		0.31	0.31	0.31	0.31	0.31	0.31	0.31				
Hardy-Weinberg p-value		0.23	0.23	0.23	0.23	0.65	0.23	0.23				

TAS2R30 (Gene ID: 259293, Transcript ID: NM_001097643.1, Protein ID: NP_001091112.1)

	T 54 G<br>V 18 V<br>rs2600355	A 521 G<br>H 174 R<br>rs200095034	T 756 G<br>F 252 L<br>rs2599404	G 943 A<br>A 315 T<br>rs199946401	Allele freq.<br>n=96	Homoz.	Heteroz.	Carrier freq.<br>n=48
H1 [1,2,4,6]	GTG	CAT	TTG	GCA	0.54	0.29	0.50	0.79
	V	H	L	A				
H2 [1,2,6]	GTT	CAT	TTT	GCA	0.44	0.19	0.50	0.69
	V	H	F	A				
H4 [2,6]	GTG	CGT	TTG	GCA	0.01	-	0.02	0.02
	V	R	L	A				
H6 [3]	GTG	CAT	TTG	ACA	0.01	-	0.02	0.02
	V	H	L	T				
Minor allele freq.	0.44	0.01	0.44	0.01				
Heteroz.	0.50	0.02	0.50	0.02				
Hardy-Weinberg p-value	0.91	0.94	0.91	0.94				

TAS2R42 (Gene ID: 353164 , Transcript ID: NM_181429.1, Protein ID: NP_852094.1)

		A 524 T<br>Y 175 F<br>rs35969491	A 561 G<br>L 187 L<br>rs1650019	T 587 C<br>F 196 S<br>rs5020531	G 763 T<br>G 255 W<br>rs1669413	A 794 G<br>Y 265 C<br>rs1451772	G 875 A<br>R 292 Q<br>rs1669412	T 930 C<br>N 310 N<br>rs1669411	G 931 C<br>A 311 P<br>rs1650017	Allele freq.<br>n=96	Homoz.	Heteroz.	Carrier freq.<br>n=48
H1 [1,2,3]	RV1 [5]	TTT<br>F	TTG<br>L	TCC<br>S	TGG<br>W	TAC<br>Y	CGA<br>R	AAC<br>N	CCT<br>P	0.52	0.25	0.54	0.79
H2 [1,2]	RV4 [5]	TAT<br>Y	TTG<br>L	TTC<br>F	TGG<br>W	TGC<br>C	CAA<br>Q	AAC<br>N	CCT<br>P	0.30	0.08	0.44	0.52
H3 [1,2,3]	RV2 [5]	TAT<br>Y	TTA<br>L	TTC<br>F	GGG<br>G	TAC<br>Y	CGA<br>R	AAT<br>N	GCT<br>A	0.18	0.02	0.31	0.33
Minor allele freq.		0.48	0.18	0.48	0.18	0.30	0.30	0.18	0.18				
Heteroz.		0.54	0.31	0.54	0.31	0.44	0.44	0.31	0.31				
Hardy-Weinberg p-value		0.55	0.62	0.55	0.62	0.79	0.79	0.62	0.62				

1 direct ascertainment from homozygous individuals
2 haplotype reconstruction using Bayesian approach (Stephens et al., 2001; Stephens and Donnelly, 2003)
3 subcloning and sequencing analysis
4 previously published (Meyerhof et al., 2010)
5 previously published (Thalmann et al., 2013)
6 previously published (Roudnitzky et al., 2011)
