# Supplementary material for: Receptor Polymorphism and Genomic Structure Interact to Shape Bitter Taste Perception
Source: PLoS Genet. 2015 Sep 25;11(9):e1005530. doi: 10.1371/journal.pgen.1005530 (PMC4583475; doi:10.1371/journal.pgen.1005530)
Supplement: S2 Table — Probability values are detailed per receptor for each common SNPs. Probability values, which reached significance level of 0.05, are underlined in grey; probability values, which reached the experiment-wide significance threshold, appear in bold type. (PDF) [file pgen.1005530.s002.pdf]

Absinthin

p-Value			Detection threshold	Recognition threshold	Weak intensity	Moderate intensity	Strong intensity	Very strong intensity
Receptor	Nucleotide	Amino acid						
TAS2R1	C 616 T	R 206 W	0.5958	0.4912	0.5633	0.5899	0.5163	0.3758
TAS2R16	G 665 A	R 222 H	0.6735	0.5388	0.2327	0.2194	0.2482	0.3324
	G 846 A	T 282 T	0.7135	0.9997	0.7011	0.5812	0.4693	0.4039
TAS2R3	C 807 T	G 269 G	0.2638	0.7822	0.9348	0.9261	0.9195	0.9164
TAS2R4	T 20 C	F 7 S	0.5835	0.9381	0.9389	0.9668	0.9643	0.9186
	G 286 C	V 96 L	0.5835	0.9381	0.9389	0.9668	0.9643	0.9186
	G 512 A	S 171 N	0.5835	0.9381	0.9389	0.9668	0.9643	0.9186
TAS2R5	G 77 T	S 26 I	0.2638	0.7822	0.9348	0.9261	0.9195	0.9164
TAS2R38	G 145 C	A 49 P	0.2291	0.5492	0.2943	0.3678	0.5300	0.7484
	T 785 C	V 262 A	0.2681	0.6049	0.3278	0.4113	0.5853	0.8061
	A 886 G	I 296 V	0.2681	0.6049	0.3278	0.4113	0.5853	0.8061
TAS2R39			1.0000	1.0000	1.0000	1.0000	1.0000	1.0000
TAS2R40	C 560 A	S 187 Y	0.2942	0.5925	0.6944	0.8847	0.8571	0.6297
TAS2R60	T 930 C	R 310 R	0.9277	0.6319	0.3105	0.3690	0.4655	0.5589
TAS2R41	G 189 A	T 63 T	0.4758	0.2860	0.3678	0.3310	0.3250	0.3598
	C 380 T	P 127 L	0.4758	0.2860	0.3678	0.3310	0.3250	0.3598
TAS2R7	G 912 A	M 304 I	0.6920	0.6749	0.5944	0.6528	0.6481	0.5583
TAS2R8	G 549 A	L 183 L	0.0871	0.2800	0.2813	0.3323	0.4616	0.6491
TAS2R9	T 560 C	V 187 A	0.0353	0.2232	0.1621	0.1316	0.1369	0.1939
TAS2R10			1.0000	1.0000	1.0000	1.0000	1.0000	1.0000
TAS2R13	A 776 G	N 259 S	0.4280	0.7233	0.7715	0.5829	0.3834	0.2708
TAS2R14	A 114 T	G 38 G	0.5491	0.8880	0.6756	0.5479	0.4012	0.3126
	G 375 A	R 125 R	0.1653	0.4309	0.6479	0.6132	0.5943	0.6095
TAS2R50	A 525 G	S 175 S	0.1569	0.4344	0.6004	0.5252	0.4797	0.4945
	G 608 A	C 203 Y	0.5698	0.6830	0.6115	0.6312	0.5799	0.4810
	G 777 A	P 259 P	0.3650	0.9896	0.9614	0.9259	0.7552	0.5535
TAS2R20	A 156 G	A 52 A	0.5891	0.7333	0.6450	0.6184	0.4946	0.3556
	A 235 G	K 79 E	0.5747	0.8422	0.8997	0.9726	0.7507	0.4212
	C 261 T	A 87 A	0.5891	0.7333	0.6450	0.6184	0.4946	0.3556
	G 421 A	V 141 I	0.3759	0.5493	0.3309	0.2192	0.1496	0.1388
	C 429 A	H 143 Q	0.5891	0.7333	0.6450	0.6184	0.4946	0.3556
	C 442 A	H 148 N	0.5891	0.7333	0.6450	0.6184	0.4946	0.3556
	A 706 G	I 236 V	0.5891	0.7333	0.6450	0.6184	0.4946	0.3556
	T 755 C	F 252 S	0.5891	0.7333	0.6450	0.6184	0.4946	0.3556
	G 764 T	R 255 L	0.5891	0.7333	0.6450	0.6184	0.4946	0.3556
TAS2R19	C 84 T	A 28 A	0.4881	0.7965	0.8857	0.9941	0.9072	0.6553
	T 418 C	L 140 L	0.0204	0.1624	0.3353	0.3035	0.3213	0.4035
	C 895 T	R 299 C	0.4881	0.7965	0.8857	0.9941	0.9072	0.6553
TAS2R31	C 103 T	R 35 W	0.5343	0.5580	0.5642	0.6958	0.7433	0.6246
	T 423 C	A 141 A	0.5747	0.8422	0.8997	0.9726	0.7507	0.4212
	T 484 A	L 162 M	0.0656	0.2123	0.2783	0.2291	0.2352	0.3181
	C 649 G	Q 217 E	0.5098	0.3809	0.4864	0.5898	0.5709	0.4145
	C 680 T	A 227 V	0.5343	0.5580	0.5642	0.6958	0.7433	0.6246
	G 718 A	V 240 I	0.5343	0.5580	0.5642	0.6958	0.7433	0.6246
	A 744 G	S 248 S	0.1569	0.4344	0.6004	0.5252	0.4797	0.4945
	C 827 G	P 276 R	0.5098	0.3809	0.4864	0.5898	0.5709	0.4145
TAS2R46	G 534 A	T 178 T	0.5098	0.3809	0.4864	0.5898	0.5709	0.4145
	T 682 A	L 228 M	0.5343	0.5580	0.5642	0.6958	0.7433	0.6246
	G 749 A	W 250 X	0.5747	0.8422	0.8997	0.9726	0.7507	0.4212
TAS2R43	Deletion		0.5343	0.5580	0.5642	0.6958	0.7433	0.6246
	G 104 C	W 35 S	0.4034	0.7756	0.8375	0.7378	0.5063	0.2933
	A 635 G	H 212 R	0.4034	0.7756	0.8375	0.7378	0.5063	0.2933
	C 663 G	T 221 T	0.4034	0.7756	0.8375	0.7378	0.5063	0.2933
TAS2R45	Deletion		0.1653	0.4309	0.6479	0.6132	0.5943	0.6095
	A 227 G	Y 76 C	0.4071	0.7855	0.8509	0.7576	0.5199	0.2917
	G 394 A	V 132 M	0.4071	0.7855	0.8509	0.7576	0.5199	0.2917
	G 630 C	Q 210 H	0.4071	0.7855	0.8509	0.7576	0.5199	0.2917
	T 703 C	F 235 L	0.4071	0.7855	0.8509	0.7576	0.5199	0.2917
	C 712 T	R 238 C	0.4108	0.8593	0.9536	0.8718	0.6616	0.4485
	G 893 C	R 298 T	0.4071	0.7855	0.8509	0.7576	0.5199	0.2917
	A 900 G	X 300 W	0.4071	0.7855	0.8509	0.7576	0.5199	0.2917
TAS2R30	T 54 G	V 18 V	0.5343	0.5580	0.5642	0.6958	0.7433	0.6246
	T 756 G	F 252 L	0.5343	0.5580	0.5642	0.6958	0.7433	0.6246
TAS2R42	A 524 T	Y 175 F	0.2996	0.9105	0.9958	0.8736	0.5762	0.3288
	A 561 G	L 187 L	0.1485	0.4325	0.6646	0.6096	0.5751	0.5878
	T 587 C	F 196 S	0.2996	0.9105	0.9958	0.8736	0.5762	0.3288
	G 763 T	G 255 W	0.1485	0.4325	0.6646	0.6096	0.5751	0.5878
	A 794 G	Y 265 C	0.5426	0.6772	0.8324	0.9823	0.8397	0.5038
	G 875 A	R 292 Q	0.5426	0.6772	0.8324	0.9823	0.8397	0.5038
	T 930 C	N 310 N	0.1485	0.4325	0.6646	0.6096	0.5751	0.5878
	G 931 C	A 311 P	0.1485	0.4325	0.6646	0.6096	0.5751	0.5878

Amarogentin

Receptor	Nucleotide	Amino acid	Detection threshold	Recognition threshold	Weak intensity	Moderate intensity	Strong intensity	Very strong intensity
	**p-Value**							
TAS2R1	C 616 T	R 206 W	0.4066	0.8346	0.9405	0.8014	0.5511	0.3567
TAS2R16	G 665 A	R 222 H	0.6474	0.2675	0.1697	0.1765	0.2265	0.3336
	G 846 A	T 282 T	0.9646	0.9990	0.9069	0.9500	0.7857	0.6539
TAS2R3	C 807 T	G 269 G	0.4644	0.4948	0.8239	0.9091	0.9244	0.8466
TAS2R4	T 20 C	F 7 S	0.3812	0.3077	0.4698	0.6027	0.7619	0.8399
	G 286 C	V 96 L	0.3812	0.3077	0.4698	0.6027	0.7619	0.8399
	G 512 A	S 171 N	0.3812	0.3077	0.4698	0.6027	0.7619	0.8399
TAS2R5	G 77 T	S 26 I	0.4644	0.4948	0.8239	0.9091	0.9244	0.8466
TAS2R38	G 145 C	A 49 P	0.2443	0.2196	0.2925	0.2953	0.3380	0.4170
	T 785 C	V 262 A	0.2539	0.3336	0.4130	0.4669	0.5771	0.7081
	A 886 G	I 296 V	0.2539	0.3336	0.4130	0.4669	0.5771	0.7081
TAS2R39			1.0000	1.0000	1.0000	1.0000	1.0000	1.0000
TAS2R40	C 560 A	S 187 Y	0.2032	0.8722	0.8919	0.7882	0.6843	0.6112
TAS2R60	T 930 C	R 310 R	0.9632	0.6152	0.3065	0.2622	0.2659	0.3251
TAS2R41	G 189 A	T 63 T	0.6573	0.3902	0.4086	0.3527	0.3394	0.3758
	C 380 T	P 127 L	0.6573	0.3902	0.4086	0.3527	0.3394	0.3758
TAS2R7	G 912 A	M 304 I	0.2739	0.2515	0.1434	0.3205	0.6601	0.8872
TAS2R8	G 549 A	L 183 L	0.8574	0.5956	0.5540	0.5316	0.5084	0.4914
TAS2R9	T 560 C	V 187 A	0.9567	0.7088	0.7217	0.8622	0.9383	0.8790
TAS2R10			1.0000	1.0000	1.0000	1.0000	1.0000	1.0000
TAS2R13	A 776 G	N 259 S	0.8821	0.9223	0.8929	0.6701	0.3797	0.2070
TAS2R14	A 114 T	G 38 G	0.8246	0.5896	0.2691	0.4014	0.5657	0.6237
	G 375 A	R 125 R	0.0694	0.0350	0.0261	0.0337	0.0702	0.1653
TAS2R50	A 525 G	S 175 S	0.0647	0.0323	0.0266	0.0325	0.0640	0.1466
	G 608 A	C 203 Y	0.6146	0.0925	0.0695	0.1141	0.2490	0.4983
	G 777 A	P 259 P	0.8677	0.9053	0.9071	0.7319	0.4876	0.3198
TAS2R20	A 156 G	A 52 A	0.4742	0.1012	0.0679	0.1302	0.3061	0.5857
	A 235 G	K 79 E	0.3440	0.3162	0.1691	0.4457	0.8320	0.7982
	C 261 T	A 87 A	0.4742	0.1012	0.0679	0.1302	0.3061	0.5857
	G 421 A	V 141 I	0.7841	0.2400	0.2527	0.2831	0.3408	0.4056
	C 429 A	H 143 Q	0.4742	0.1012	0.0679	0.1302	0.3061	0.5857
	C 442 A	H 148 N	0.4742	0.1012	0.0679	0.1302	0.3061	0.5857
	A 706 G	I 236 V	0.4742	0.1012	0.0679	0.1302	0.3061	0.5857
	T 755 C	F 252 S	0.4742	0.1012	0.0679	0.1302	0.3061	0.5857
	G 764 T	R 255 L	0.4742	0.1012	0.0679	0.1302	0.3061	0.5857
TAS2R19	C 84 T	A 28 A	0.1786	0.0340	0.0082	0.0274	0.1301	0.4104
	T 418 C	L 140 L	0.0067	0.0086	0.0175	0.0265	0.0640	0.1611
	C 895 T	R 299 C	0.1786	0.0340	0.0082	0.0274	0.1301	0.4104
TAS2R31	C 103 T	R 35 W	0.0662	0.0091	**0.0009**	0.0032	0.0209	0.0968
	T 423 C	A 141 A	0.3440	0.3162	0.1691	0.4457	0.8320	0.7982
	T 484 A	L 162 M	0.0055	0.0122	0.0142	0.0197	0.0515	0.1514
	C 649 G	Q 217 E	0.5053	0.0947	0.0941	0.0443	0.0193	0.0122
	C 680 T	A 227 V	0.0662	0.0091	**0.0009**	0.0032	0.0209	0.0968
	G 718 A	V 240 I	0.0662	0.0091	**0.0009**	0.0032	0.0209	0.0968
	A 744 G	S 248 S	0.0647	0.0323	0.0266	0.0325	0.0640	0.1466
	C 827 G	P 276 R	0.5053	0.0947	0.0941	0.0443	0.0193	0.0122
TAS2R46	G 534 A	T 178 T	0.5053	0.0947	0.0941	0.0443	0.0193	0.0122
	T 682 A	L 228 M	0.0662	0.0091	**0.0009**	0.0032	0.0209	0.0968
	G 749 A	W 250 X	0.3440	0.3162	0.1691	0.4457	0.8320	0.7982
TAS2R43	Deletion		0.0662	0.0091	**0.0009**	0.0032	0.0209	0.0968
	G 104 C	W 35 S	0.1410	0.0140	0.0054	0.0148	0.0626	0.1882
	A 635 G	H 212 R	0.1410	0.0140	0.0054	0.0148	0.0626	0.1882
	C 663 G	T 221 T	0.1410	0.0140	0.0054	0.0148	0.0626	0.1882
TAS2R45	Deletion		0.0694	0.0350	0.0261	0.0337	0.0702	0.1653
	A 227 G	Y 76 C	0.1012	0.0113	0.0063	0.0211	0.0957	0.2670
	G 394 A	V 132 M	0.1012	0.0113	0.0063	0.0211	0.0957	0.2670
	G 630 C	Q 210 H	0.1012	0.0113	0.0063	0.0211	0.0957	0.2670
	T 703 C	F 235 L	0.1012	0.0113	0.0063	0.0211	0.0957	0.2670
	C 712 T	R 238 C	0.3247	0.2311	0.1819	0.2563	0.3328	0.3526
	G 893 C	R 298 T	0.1012	0.0113	0.0063	0.0211	0.0957	0.2670
	A 900 G	X 300 W	0.1012	0.0113	0.0063	0.0211	0.0957	0.2670
TAS2R30	T 54 G	V 18 V	0.0662	0.0091	**0.0009**	0.0032	0.0209	0.0968
	T 756 G	F 252 L	0.0662	0.0091	**0.0009**	0.0032	0.0209	0.0968
TAS2R42	A 524 T	Y 175 F	0.7951	0.7463	0.9882	0.8655	0.5150	0.2522
	A 561 G	L 187 L	0.0845	0.0397	0.0302	0.0329	0.0556	0.1157
	T 587 C	F 196 S	0.7951	0.7463	0.9882	0.8655	0.5150	0.2522
	G 763 T	G 255 W	0.0845	0.0397	0.0302	0.0329	0.0556	0.1157
	A 794 G	Y 265 C	0.3117	0.2011	0.1090	0.3151	0.7380	0.9346
	G 875 A	R 292 Q	0.3117	0.2011	0.1090	0.3151	0.7380	0.9346
	T 930 C	N 310 N	0.0845	0.0397	0.0302	0.0329	0.0556	0.1157
	G 931 C	A 311 P	0.0845	0.0397	0.0302	0.0329	0.0556	0.1157

Cascarillin

p-Value								
Receptor	Nucleotide	Amino acid	Detection threshold	Recognition threshold	Weak intensity	Moderate intensity	Strong intensity	Very strong intensity
TAS2R1	C 616 T	R 206 W	0.4245	0.7421	0.6959	0.6432	0.5825	0.5855
TAS2R16	G 665 A	R 222 H	0.6531	0.5324	0.5139	0.5027	0.5120	0.5799
	G 846 A	T 282 T	0.5495	0.7069	0.5901	0.5956	0.6336	0.7475
TAS2R3	C 807 T	G 269 G	0.1025	0.4806	0.4265	0.4424	0.5207	0.7248
TAS2R4	T 20 C	F 7 S	0.4081	0.7154	0.7892	0.7842	0.7924	0.8286
	G 286 C	V 96 L	0.4081	0.7154	0.7892	0.7842	0.7924	0.8286
	G 512 A	S 171 N	0.4081	0.7154	0.7892	0.7842	0.7924	0.8286
TAS2R5	G 77 T	S 26 I	0.1025	0.4806	0.4265	0.4424	0.5207	0.7248
TAS2R38	G 145 C	A 49 P	0.2653	0.5104	0.4560	0.3945	0.3347	0.3684
	T 785 C	V 262 A	0.2474	0.5065	0.4561	0.3961	0.3395	0.3801
	A 886 G	I 296 V	0.2474	0.5065	0.4561	0.3961	0.3395	0.3801
TAS2R39			1.0000	1.0000	1.0000	1.0000	1.0000	1.0000
TAS2R40	C 560 A	S 187 Y	0.0518	0.3424	0.4578	0.5744	0.8443	0.7158
TAS2R60	T 930 C	R 310 R	0.4957	0.6966	0.5856	0.5397	0.5128	0.5922
TAS2R41	G 189 A	T 63 T	0.9231	0.6227	0.4836	0.3229	0.1041	0.0157
	C 380 T	P 127 L	0.9231	0.6227	0.4836	0.3229	0.1041	0.0157
TAS2R7	G 912 A	M 304 I	0.3887	0.7558	0.4992	0.5400	0.6654	0.9002
TAS2R8	G 549 A	L 183 L	0.5138	0.5496	0.3230	0.3379	0.3707	0.4144
TAS2R9	T 560 C	V 187 A	0.0690	0.5005	0.3885	0.3810	0.3657	0.3721
TAS2R10			1.0000	1.0000	1.0000	1.0000	1.0000	1.0000
TAS2R13	A 776 G	N 259 S	0.8736	0.7454	0.5104	0.6829	0.8148	0.4580
TAS2R14	A 114 T	G 38 G	0.7720	0.3755	0.1770	0.2125	0.3407	0.6736
	G 375 A	R 125 R	0.2068	0.1569	0.4151	0.4163	0.3857	0.3060
TAS2R50	A 525 G	S 175 S	0.2185	0.1529	0.4196	0.4312	0.4154	0.3375
	G 608 A	C 203 Y	0.9062	0.6463	0.6902	0.7835	0.9309	0.9380
	G 777 A	P 259 P	0.9298	0.8657	0.8935	0.9940	0.8496	0.3464
TAS2R20	A 156 G	A 52 A	0.9778	0.5697	0.5700	0.7143	0.9380	0.8730
	A 235 G	K 79 E	0.6224	0.5455	0.3632	0.5211	0.8557	0.9361
	C 261 T	A 87 A	0.9778	0.5697	0.5700	0.7143	0.9380	0.8730
	G 421 A	V 141 I	0.1887	0.5920	0.6554	0.5617	0.4363	0.3907
	C 429 A	H 143 Q	0.9778	0.5697	0.5700	0.7143	0.9380	0.8730
	C 442 A	H 148 N	0.9778	0.5697	0.5700	0.7143	0.9380	0.8730
	A 706 G	I 236 V	0.9778	0.5697	0.5700	0.7143	0.9380	0.8730
	T 755 C	F 252 S	0.9778	0.5697	0.5700	0.7143	0.9380	0.8730
	G 764 T	R 255 L	0.9778	0.5697	0.5700	0.7143	0.9380	0.8730
TAS2R19	C 84 T	A 28 A	0.7151	0.8223	0.7922	0.8670	0.9759	0.9679
	T 418 C	L 140 L	0.0243	0.0097	0.0752	0.1253	0.2610	0.3399
	C 895 T	R 299 C	0.7151	0.8223	0.7922	0.8670	0.9759	0.9679
TAS2R31	C 103 T	R 35 W	0.5098	0.5957	0.4077	0.4076	0.4208	0.4454
	T 423 C	A 141 A	0.6224	0.5455	0.3632	0.5211	0.8557	0.9361
	T 484 A	L 162 M	0.0661	0.0282	0.1082	0.1312	0.1864	0.2593
	C 649 G	Q 217 E	0.2230	0.1676	0.2392	0.2000	0.1625	0.1792
	C 680 T	A 227 V	0.5098	0.5957	0.4077	0.4076	0.4208	0.4454
	G 718 A	V 240 I	0.5098	0.5957	0.4077	0.4076	0.4208	0.4454
	A 744 G	S 248 S	0.2185	0.1529	0.4196	0.4312	0.4154	0.3375
	C 827 G	P 276 R	0.2230	0.1676	0.2392	0.2000	0.1625	0.1792
TAS2R46	G 534 A	T 178 T	0.2230	0.1676	0.2392	0.2000	0.1625	0.1792
	T 682 A	L 228 M	0.5098	0.5957	0.4077	0.4076	0.4208	0.4454
	G 749 A	W 250 X	0.6224	0.5455	0.3632	0.5211	0.8557	0.9361
TAS2R43	Deletion		0.5098	0.5957	0.4077	0.4076	0.4208	0.4454
	G 104 C	W 35 S	0.6490	0.3804	0.6488	0.7215	0.7200	0.4450
	A 635 G	H 212 R	0.6490	0.3804	0.6488	0.7215	0.7200	0.4450
	C 663 G	T 221 T	0.6490	0.3804	0.6488	0.7215	0.7200	0.4450
TAS2R45	Deletion		0.2068	0.1569	0.4151	0.4163	0.3857	0.3060
	A 227 G	Y 76 C	0.6275	0.4007	0.6687	0.7704	0.7978	0.4901
	G 394 A	V 132 M	0.6275	0.4007	0.6687	0.7704	0.7978	0.4901
	G 630 C	Q 210 H	0.6275	0.4007	0.6687	0.7704	0.7978	0.4901
	T 703 C	F 235 L	0.6275	0.4007	0.6687	0.7704	0.7978	0.4901
	C 712 T	R 238 C	0.6463	0.5398	0.8116	0.8648	0.8267	0.4787
	G 893 C	R 298 T	0.6275	0.4007	0.6687	0.7704	0.7978	0.4901
	A 900 G	X 300 W	0.6275	0.4007	0.6687	0.7704	0.7978	0.4901
TAS2R30	T 54 G	V 18 V	0.5098	0.5957	0.4077	0.4076	0.4208	0.4454
	T 756 G	F 252 L	0.5098	0.5957	0.4077	0.4076	0.4208	0.4454
TAS2R42	A 524 T	Y 175 F	0.8910	0.5763	0.7211	0.9218	0.8401	0.2390
	A 561 G	L 187 L	0.2079	0.1469	0.4208	0.4284	0.3749	0.2387
	T 587 C	F 196 S	0.8910	0.5763	0.7211	0.9218	0.8401	0.2390
	G 763 T	G 255 W	0.2079	0.1469	0.4208	0.4284	0.3749	0.2387
	A 794 G	Y 265 C	0.4699	0.4930	0.3366	0.4788	0.7979	0.9366
	G 875 A	R 292 Q	0.4699	0.4930	0.3366	0.4788	0.7979	0.9366
	T 930 C	N 310 N	0.2079	0.1469	0.4208	0.4284	0.3749	0.2387
	G 931 C	A 311 P	0.2079	0.1469	0.4208	0.4284	0.3749	0.2387

Grosheimin

Receptor	Nucleotide	Amino acid	Detection threshold	Recognition threshold	Weak intensity	Moderate intensity	Strong intensity	Very strong intensity
	**p-Value**							
TAS2R1	C 616 T	R 206 W	0.1567	0.4057	0.4619	0.4239	0.4181	0.4753
TAS2R16	G 665 A	R 222 H	0.2492	0.8157	0.9184	0.8627	0.6097	0.3452
	G 846 A	T 282 T	0.3795	0.5775	0.3473	0.4232	0.5792	0.8086
TAS2R3	C 807 T	G 269 G	0.9931	0.9550	0.9839	0.8917	0.5886	0.3086
TAS2R4	T 20 C	F 7 S	0.5058	0.8134	0.5846	0.4238	0.2825	0.2226
	G 286 C	V 96 L	0.5058	0.8134	0.5846	0.4238	0.2825	0.2226
	G 512 A	S 171 N	0.5058	0.8134	0.5846	0.4238	0.2825	0.2226
TAS2R5	G 77 T	S 26 I	0.9931	0.9550	0.9839	0.8917	0.5886	0.3086
TAS2R38	G 145 C	A 49 P	0.4210	0.3280	0.3482	0.2002	0.1009	0.0684
	T 785 C	V 262 A	0.3780	0.3683	0.4744	0.3311	0.2134	0.1665
	A 886 G	I 296 V	0.3780	0.3683	0.4744	0.3311	0.2134	0.1665
TAS2R39			1.0000	1.0000	1.0000	1.0000	1.0000	1.0000
TAS2R40	C 560 A	S 187 Y	0.0718	0.2762	0.4455	0.7456	0.7753	0.3680
TAS2R60	T 930 C	R 310 R	0.7430	0.7279	0.7168	0.4683	0.2250	0.1114
TAS2R41	G 189 A	T 63 T	0.8126	0.2832	0.3462	0.3197	0.3417	0.4386
	C 380 T	P 127 L	0.8126	0.2832	0.3462	0.3197	0.3417	0.4386
TAS2R7	G 912 A	M 304 I	0.5938	0.3363	0.1472	0.0727	0.0292	0.0168
TAS2R8	G 549 A	L 183 L	0.0641	0.0470	0.1098	0.1047	0.1319	0.2250
TAS2R9	T 560 C	V 187 A	0.0164	0.0208	0.0132	0.0107	0.0162	0.0489
TAS2R10			1.0000	1.0000	1.0000	1.0000	1.0000	1.0000
TAS2R13	A 776 G	N 259 S	0.5608	0.8474	0.8097	0.6476	0.3072	0.1032
TAS2R14	A 114 T	G 38 G	0.0078	0.0816	0.0475	0.0140	0.0042	0.0035
	G 375 A	R 125 R	**0.0027**	0.0066	0.0106	0.0131	0.0348	0.1402
TAS2R50	A 525 G	S 175 S	0.0303	0.0124	0.0248	0.0418	0.1235	0.3869
	G 608 A	C 203 Y	0.0212	0.4203	0.2049	0.0388	**0.0024**	**0.0002**
	G 777 A	P 259 P	0.3944	0.9311	0.9578	0.9481	0.5699	0.2104
TAS2R20	A 156 G	A 52 A	0.0093	0.3226	0.1349	0.0208	**0.0010**	**0.0001**
	A 235 G	K 79 E	0.0244	0.1417	0.0964	0.0216	**0.0027**	**0.0007**
	C 261 T	A 87 A	0.0093	0.3226	0.1349	0.0208	**0.0010**	**0.0001**
	G 421 A	V 141 I	0.0547	0.1842	0.0373	0.0082	**0.0014**	**0.0007**
	C 429 A	H 143 Q	0.0093	0.3226	0.1349	0.0208	**0.0010**	**0.0001**
	C 442 A	H 148 N	0.0093	0.3226	0.1349	0.0208	**0.0010**	**0.0001**
	A 706 G	I 236 V	0.0093	0.3226	0.1349	0.0208	**0.0010**	**0.0001**
	T 755 C	F 252 S	0.0093	0.3226	0.1349	0.0208	**0.0010**	**0.0001**
	G 764 T	R 255 L	0.0093	0.3226	0.1349	0.0208	**0.0010**	**0.0001**
TAS2R19	C 84 T	A 28 A	**0.0001**	0.0056	**0.0002**	**0.0001**	**0.0001**	**0.0001**
	T 418 C	L 140 L	**0.0008**	**0.0016**	**0.0020**	0.0054	0.0370	0.2426
	C 895 T	R 299 C	**0.0001**	0.0056	**0.0002**	**0.0001**	**0.0001**	**0.0001**
TAS2R31	C 103 T	R 35 W	**0.0001**	0.0125	**0.0012**	**0.0001**	**0.0001**	**0.0001**
	T 423 C	A 141 A	0.0244	0.1417	0.0964	0.0216	**0.0027**	**0.0007**
	T 484 A	L 162 M	**0.0011**	**0.0010**	**0.0017**	**0.0025**	0.0126	0.1045
	C 649 G	Q 217 E	0.0480	0.0847	0.0874	0.1116	0.1875	0.3318
	C 680 T	A 227 V	**0.0001**	0.0125	**0.0012**	**0.0001**	**0.0001**	**0.0001**
	G 718 A	V 240 I	**0.0001**	0.0125	**0.0012**	**0.0001**	**0.0001**	**0.0001**
	A 744 G	S 248 S	0.0303	0.0124	0.0248	0.0418	0.1235	0.3869
	C 827 G	P 276 R	0.0480	0.0847	0.0874	0.1116	0.1875	0.3318
TAS2R46	G 534 A	T 178 T	0.0480	0.0847	0.0874	0.1116	0.1875	0.3318
	T 682 A	L 228 M	**0.0001**	0.0125	**0.0012**	**0.0001**	**0.0001**	**0.0001**
	G 749 A	W 250 X	0.0244	0.1417	0.0964	0.0216	**0.0027**	**0.0007**
TAS2R43	Deletion		**0.0001**	0.0125	**0.0012**	**0.0001**	**0.0001**	**0.0001**
	G 104 C	W 35 S	**0.0001**	0.0207	0.0086	**0.0009**	**0.0001**	**0.0001**
	A 635 G	H 212 R	**0.0001**	0.0207	0.0086	**0.0009**	**0.0001**	**0.0001**
	C 663 G	T 221 T	**0.0001**	0.0207	0.0086	**0.0009**	**0.0001**	**0.0001**
TAS2R45	Deletion		**0.0027**	0.0066	0.0106	0.0131	0.0348	0.1402
	A 227 G	Y 76 C	**0.0005**	0.0177	0.0071	**0.0006**	**0.0001**	**0.0001**
	G 394 A	V 132 M	**0.0005**	0.0177	0.0071	**0.0006**	**0.0001**	**0.0001**
	G 630 C	Q 210 H	**0.0005**	0.0177	0.0071	**0.0006**	**0.0001**	**0.0001**
	T 703 C	F 235 L	**0.0005**	0.0177	0.0071	**0.0006**	**0.0001**	**0.0001**
	C 712 T	R 238 C	0.0100	0.0254	0.0355	0.0083	**0.0009**	**0.0002**
	G 893 C	R 298 T	**0.0005**	0.0177	0.0071	**0.0006**	**0.0001**	**0.0001**
	A 900 G	X 300 W	**0.0005**	0.0177	0.0071	**0.0006**	**0.0001**	**0.0001**
TAS2R30	T 54 G	V 18 V	**0.0001**	0.0125	**0.0012**	**0.0001**	**0.0001**	**0.0001**
	T 756 G	F 252 L	**0.0001**	0.0125	**0.0012**	**0.0001**	**0.0001**	**0.0001**
TAS2R42	A 524 T	Y 175 F	0.3654	0.9244	0.9829	0.7967	0.3889	0.1342
	A 561 G	L 187 L	**0.0019**	0.0065	0.0109	0.0167	0.0536	0.2182
	T 587 C	F 196 S	0.3654	0.9244	0.9829	0.7967	0.3889	0.1342
	G 763 T	G 255 W	**0.0019**	0.0065	0.0109	0.0167	0.0536	0.2182
	A 794 G	Y 265 C	0.0201	0.1306	0.0708	0.0163	**0.0026**	**0.0010**
	G 875 A	R 292 Q	0.0201	0.1306	0.0708	0.0163	**0.0026**	**0.0010**
	T 930 C	N 310 N	**0.0019**	0.0065	0.0109	0.0167	0.0536	0.2182
	G 931 C	A 311 P	**0.0019**	0.0065	0.0109	0.0167	0.0536	0.2182

Quassin

p-Value			Detection threshold	Recognition threshold	Weak intensity	Moderate intensity	Strong intensity	Very strong intensity
Receptor	Nucleotide	Amino acid						
TAS2R1	C 616 T	R 206 W	0.7271	0.6368	0.9102	0.8432	0.6237	0.4041
TAS2R16	G 665 A	R 222 H	0.6932	0.2884	0.1052	0.0708	0.0632	0.0950
	G 846 A	T 282 T	0.5977	0.7872	0.5100	0.4606	0.4309	0.4452
TAS2R3	C 807 T	G 269 G	0.1789	0.4239	0.5830	0.5674	0.5898	0.6559
TAS2R4	T 20 C	F 7 S	0.2649	0.3936	0.5533	0.5710	0.6165	0.6728
	G 286 C	V 96 L	0.2649	0.3936	0.5533	0.5710	0.6165	0.6728
	G 512 A	S 171 N	0.2649	0.3936	0.5533	0.5710	0.6165	0.6728
TAS2R5	G 77 T	S 26 I	0.1789	0.4239	0.5830	0.5674	0.5898	0.6559
TAS2R38	G 145 C	A 49 P	0.0331	0.1492	0.1186	0.0958	0.1030	0.1594
	T 785 C	V 262 A	0.0711	0.2303	0.1514	0.1349	0.1548	0.2329
	A 886 G	I 296 V	0.0711	0.2303	0.1514	0.1349	0.1548	0.2329
TAS2R39			1.0000	1.0000	1.0000	1.0000	1.0000	1.0000
TAS2R40	C 560 A	S 187 Y	0.3521	0.5463	0.9245	0.8159	0.5159	0.3128
TAS2R60	T 930 C	R 310 R	0.5704	0.4602	0.5805	0.5914	0.6196	0.6538
TAS2R41	G 189 A	T 63 T	0.4661	0.2811	0.2989	0.2344	0.1883	0.1815
	C 380 T	P 127 L	0.4661	0.2811	0.2989	0.2344	0.1883	0.1815
TAS2R7	G 912 A	M 304 I	0.0633	0.0969	0.0982	0.1667	0.3703	0.7103
TAS2R8	G 549 A	L 183 L	0.1020	0.2785	0.6016	0.6498	0.7106	0.7423
TAS2R9	T 560 C	V 187 A	0.1158	0.2873	0.7161	0.5712	0.3702	0.2362
TAS2R10			1.0000	1.0000	1.0000	1.0000	1.0000	1.0000
TAS2R13	A 776 G	N 259 S	0.1668	0.3889	0.9192	0.7464	0.4984	0.3221
TAS2R14	A 114 T	G 38 G	0.8342	0.9056	0.3414	0.4050	0.5234	0.6496
	G 375 A	R 125 R	0.0112	0.0145	0.0122	0.0134	0.0235	0.0536
TAS2R50	A 525 G	S 175 S	0.0066	0.0128	0.0115	0.0109	0.0159	0.0322
	G 608 A	C 203 Y	0.3638	0.2984	0.3088	0.3768	0.5329	0.7452
	G 777 A	P 259 P	0.2105	0.5579	0.9608	0.8468	0.6090	0.3964
TAS2R20	A 156 G	A 52 A	0.3532	0.3434	0.3110	0.3908	0.5559	0.7524
	A 235 G	K 79 E	0.2578	0.2987	0.1259	0.2794	0.6526	0.9710
	C 261 T	A 87 A	0.3532	0.3434	0.3110	0.3908	0.5559	0.7524
	G 421 A	V 141 I	0.1354	0.3136	0.4314	0.4643	0.5621	0.7121
	C 429 A	H 143 Q	0.3532	0.3434	0.3110	0.3908	0.5559	0.7524
	C 442 A	H 148 N	0.3532	0.3434	0.3110	0.3908	0.5559	0.7524
	A 706 G	I 236 V	0.3532	0.3434	0.3110	0.3908	0.5559	0.7524
	T 755 C	F 252 S	0.3532	0.3434	0.3110	0.3908	0.5559	0.7524
	G 764 T	R 255 L	0.3532	0.3434	0.3110	0.3908	0.5559	0.7524
TAS2R19	C 84 T	A 28 A	0.1317	0.0872	0.0336	0.0625	0.1816	0.4863
	T 418 C	L 140 L	0.0095	0.0109	0.0099	0.0148	0.0330	0.0738
	C 895 T	R 299 C	0.1317	0.0872	0.0336	0.0625	0.1816	0.4863
TAS2R31	C 103 T	R 35 W	0.0585	0.0270	0.0058	0.0106	0.0371	0.1383
	T 423 C	A 141 A	0.2578	0.2987	0.1259	0.2794	0.6526	0.9710
	T 484 A	L 162 M	0.0101	0.0116	0.0077	0.0107	0.0278	0.0865
	C 649 G	Q 217 E	0.1337	0.0421	0.2477	0.1926	0.1311	0.0946
	C 680 T	A 227 V	0.0585	0.0270	0.0058	0.0106	0.0371	0.1383
	G 718 A	V 240 I	0.0585	0.0270	0.0058	0.0106	0.0371	0.1383
	A 744 G	S 248 S	0.0066	0.0128	0.0115	0.0109	0.0159	0.0322
	C 827 G	P 276 R	0.1337	0.0421	0.2477	0.1926	0.1311	0.0946
TAS2R46	G 534 A	T 178 T	0.1337	0.0421	0.2477	0.1926	0.1311	0.0946
	T 682 A	L 228 M	0.0585	0.0270	0.0058	0.0106	0.0371	0.1383
	G 749 A	W 250 X	0.2578	0.2987	0.1259	0.2794	0.6526	0.9710
TAS2R43	Deletion		0.0585	0.0270	0.0058	0.0106	0.0371	0.1383
	G 104 C	W 35 S	0.0371	0.0310	0.0197	0.0315	0.0739	0.1616
	A 635 G	H 212 R	0.0371	0.0310	0.0197	0.0315	0.0739	0.1616
	C 663 G	T 221 T	0.0371	0.0310	0.0197	0.0315	0.0739	0.1616
TAS2R45	Deletion		0.0112	0.0145	0.0122	0.0134	0.0235	0.0536
	A 227 G	Y 76 C	0.0345	0.0318	0.0191	0.0362	0.0933	0.1962
	G 394 A	V 132 M	0.0345	0.0318	0.0191	0.0362	0.0933	0.1962
	G 630 C	Q 210 H	0.0345	0.0318	0.0191	0.0362	0.0933	0.1962
	T 703 C	F 235 L	0.0345	0.0318	0.0191	0.0362	0.0933	0.1962
	C 712 T	R 238 C	0.0466	0.0912	0.0964	0.1290	0.1858	0.2323
	G 893 C	R 298 T	0.0345	0.0318	0.0191	0.0362	0.0933	0.1962
	A 900 G	X 300 W	0.0345	0.0318	0.0191	0.0362	0.0933	0.1962
TAS2R30	T 54 G	V 18 V	0.0585	0.0270	0.0058	0.0106	0.0371	0.1383
	T 756 G	F 252 L	0.0585	0.0270	0.0058	0.0106	0.0371	0.1383
TAS2R42	A 524 T	Y 175 F	0.1668	0.3737	0.9303	0.8529	0.5701	0.3047
	A 561 G	L 187 L	0.0142	0.0149	0.0131	0.0133	0.0206	0.0424
	T 587 C	F 196 S	0.1668	0.3737	0.9303	0.8529	0.5701	0.3047
	G 763 T	G 255 W	0.0142	0.0149	0.0131	0.0133	0.0206	0.0424
	A 794 G	Y 265 C	0.1606	0.1835	0.0863	0.2100	0.5628	0.9487
	G 875 A	R 292 Q	0.1606	0.1835	0.0863	0.2100	0.5628	0.9487
	T 930 C	N 310 N	0.0142	0.0149	0.0131	0.0133	0.0206	0.0424
	G 931 C	A 311 P	0.0142	0.0149	0.0131	0.0133	0.0206	0.0424

Quinine

Receptor	Nucleotide	Amino acid	Detection threshold	Recognition threshold	Weak intensity	Moderate intensity	Strong intensity	Very strong intensity
	**p-Value**							
TAS2R1	C 616 T	R 206 W	0.6587	0.4857	0.4812	0.5914	0.6731	0.5422
TAS2R16	G 665 A	R 222 H	0.9215	0.3397	0.1660	0.1201	0.0947	0.1242
	G 846 A	T 282 T	0.9300	0.4469	0.7616	0.7588	0.7724	0.8229
TAS2R3	C 807 T	G 269 G	0.4284	0.4878	0.6780	0.5720	0.4598	0.4188
TAS2R4	T 20 C	F 7 S	0.2821	0.1837	0.4587	0.4163	0.3991	0.4566
	G 286 C	V 96 L	0.2821	0.1837	0.4587	0.4163	0.3991	0.4566
	G 512 A	S 171 N	0.2821	0.1837	0.4587	0.4163	0.3991	0.4566
TAS2R5	G 77 T	S 26 I	0.4284	0.4878	0.6780	0.5720	0.4598	0.4188
TAS2R38	G 145 C	A 49 P	0.2198	0.3874	0.3910	0.2177	0.0870	0.0453
	T 785 C	V 262 A	0.6240	0.5494	0.5699	0.3661	0.1666	0.0787
	A 886 G	I 296 V	0.6240	0.5494	0.5699	0.3661	0.1666	0.0787
TAS2R39			1.0000	1.0000	1.0000	1.0000	1.0000	1.0000
TAS2R40	C 560 A	S 187 Y	0.2945	0.4156	0.7706	0.9841	0.6729	0.3617
TAS2R60	T 930 C	R 310 R	0.5580	0.5755	0.6272	0.5437	0.4230	0.3358
TAS2R41	G 189 A	T 63 T	0.7311	0.1675	0.2782	0.2272	0.1365	0.0677
	C 380 T	P 127 L	0.7311	0.1675	0.2782	0.2272	0.1365	0.0677
TAS2R7	G 912 A	M 304 I	0.1000	0.2227	0.0978	0.1040	0.1461	0.2769
TAS2R8	G 549 A	L 183 L	0.3493	0.8581	0.5742	0.5546	0.5359	0.5409
TAS2R9	T 560 C	V 187 A	0.2438	0.7160	0.7261	0.6792	0.6053	0.5479
TAS2R10			1.0000	1.0000	1.0000	1.0000	1.0000	1.0000
TAS2R13	A 776 G	N 259 S	0.3569	0.8405	0.9898	0.9863	0.9814	0.9773
TAS2R14	A 114 T	G 38 G	0.2160	0.2461	0.2637	0.2233	0.2113	0.2937
	G 375 A	R 125 R	0.0107	0.0225	0.0522	0.0478	0.0643	0.1572
TAS2R50	A 525 G	S 175 S	0.0082	0.0262	0.0642	0.0554	0.0650	0.1368
	G 608 A	C 203 Y	0.2242	0.4229	0.5339	0.4848	0.4532	0.5054
	G 777 A	P 259 P	0.4524	0.8365	0.9926	0.9953	0.9955	0.9899
TAS2R20	A 156 G	A 52 A	0.2360	0.4174	0.4447	0.4333	0.4682	0.6148
	A 235 G	K 79 E	**0.0016**	0.0129	0.0323	0.0486	0.1303	0.4571
	C 261 T	A 87 A	0.2360	0.4174	0.4447	0.4333	0.4682	0.6148
	G 421 A	V 141 I	0.1606	0.6137	0.6670	0.6744	0.7268	0.8435
	C 429 A	H 143 Q	0.2360	0.4174	0.4447	0.4333	0.4682	0.6148
	C 442 A	H 148 N	0.2360	0.4174	0.4447	0.4333	0.4682	0.6148
	A 706 G	I 236 V	0.2360	0.4174	0.4447	0.4333	0.4682	0.6148
	T 755 C	F 252 S	0.2360	0.4174	0.4447	0.4333	0.4682	0.6148
	G 764 T	R 255 L	0.2360	0.4174	0.4447	0.4333	0.4682	0.6148
TAS2R19	C 84 T	A 28 A	0.0590	0.0595	0.1052	0.1097	0.1626	0.3687
	T 418 C	L 140 L	0.0142	0.0123	0.0552	0.0629	0.0983	0.1962
	C 895 T	R 299 C	0.0590	0.0595	0.1052	0.1097	0.1626	0.3687
TAS2R31	C 103 T	R 35 W	0.0217	0.0169	0.0178	0.0158	0.0244	0.0903
	T 423 C	A 141 A	**0.0016**	0.0129	0.0323	0.0486	0.1303	0.4571
	T 484 A	L 162 M	0.0196	0.0164	0.0342	0.0443	0.0982	0.2959
	C 649 G	Q 217 E	0.4290	0.2199	0.2308	0.2173	0.2368	0.3282
	C 680 T	A 227 V	0.0217	0.0169	0.0178	0.0158	0.0244	0.0903
	G 718 A	V 240 I	0.0217	0.0169	0.0178	0.0158	0.0244	0.0903
	A 744 G	S 248 S	0.0082	0.0262	0.0642	0.0554	0.0650	0.1368
	C 827 G	P 276 R	0.4290	0.2199	0.2308	0.2173	0.2368	0.3282
TAS2R46	G 534 A	T 178 T	0.4290	0.2199	0.2308	0.2173	0.2368	0.3282
	T 682 A	L 228 M	0.0217	0.0169	0.0178	0.0158	0.0244	0.0903
	G 749 A	W 250 X	**0.0016**	0.0129	0.0323	0.0486	0.1303	0.4571
TAS2R43	Deletion		0.0217	0.0169	0.0178	0.0158	0.0244	0.0903
	G 104 C	W 35 S	0.0183	0.0517	0.1188	0.1089	0.1497	0.3593
	A 635 G	H 212 R	0.0183	0.0517	0.1188	0.1089	0.1497	0.3593
	C 663 G	T 221 T	0.0183	0.0517	0.1188	0.1089	0.1497	0.3593
TAS2R45	Deletion		0.0107	0.0225	0.0522	0.0478	0.0643	0.1572
	A 227 G	Y 76 C	0.0180	0.0511	0.1243	0.1227	0.1853	0.4440
	G 394 A	V 132 M	0.0180	0.0511	0.1243	0.1227	0.1853	0.4440
	G 630 C	Q 210 H	0.0180	0.0511	0.1243	0.1227	0.1853	0.4440
	T 703 C	F 235 L	0.0180	0.0511	0.1243	0.1227	0.1853	0.4440
	C 712 T	R 238 C	0.0077	0.0486	0.1577	0.1650	0.2563	0.5528
	G 893 C	R 298 T	0.0180	0.0511	0.1243	0.1227	0.1853	0.4440
	A 900 G	X 300 W	0.0180	0.0511	0.1243	0.1227	0.1853	0.4440
TAS2R30	T 54 G	V 18 V	0.0217	0.0169	0.0178	0.0158	0.0244	0.0903
	T 756 G	F 252 L	0.0217	0.0169	0.0178	0.0158	0.0244	0.0903
TAS2R42	A 524 T	Y 175 F	0.4223	0.7075	0.8628	0.9086	0.9554	0.9456
	A 561 G	L 187 L	0.0213	0.0267	0.0693	0.0638	0.0821	0.1792
	T 587 C	F 196 S	0.4223	0.7075	0.8628	0.9086	0.9554	0.9456
	G 763 T	G 255 W	0.0213	0.0267	0.0693	0.0638	0.0821	0.1792
	A 794 G	Y 265 C	0.0035	0.0087	0.0263	0.0382	0.1036	0.3971
	G 875 A	R 292 Q	0.0035	0.0087	0.0263	0.0382	0.1036	0.3971
	T 930 C	N 310 N	0.0213	0.0267	0.0693	0.0638	0.0821	0.1792
	G 931 C	A 311 P	0.0213	0.0267	0.0693	0.0638	0.0821	0.1792
