# Supplementary figures and images for: Receptor Polymorphism and Genomic Structure Interact to Shape Bitter Taste Perception
Source: PLoS Genet. 2015 Sep 25;11(9):e1005530. doi: 10.1371/journal.pgen.1005530 (PMC4583475; doi:10.1371/journal.pgen.1005530)

Absinthin

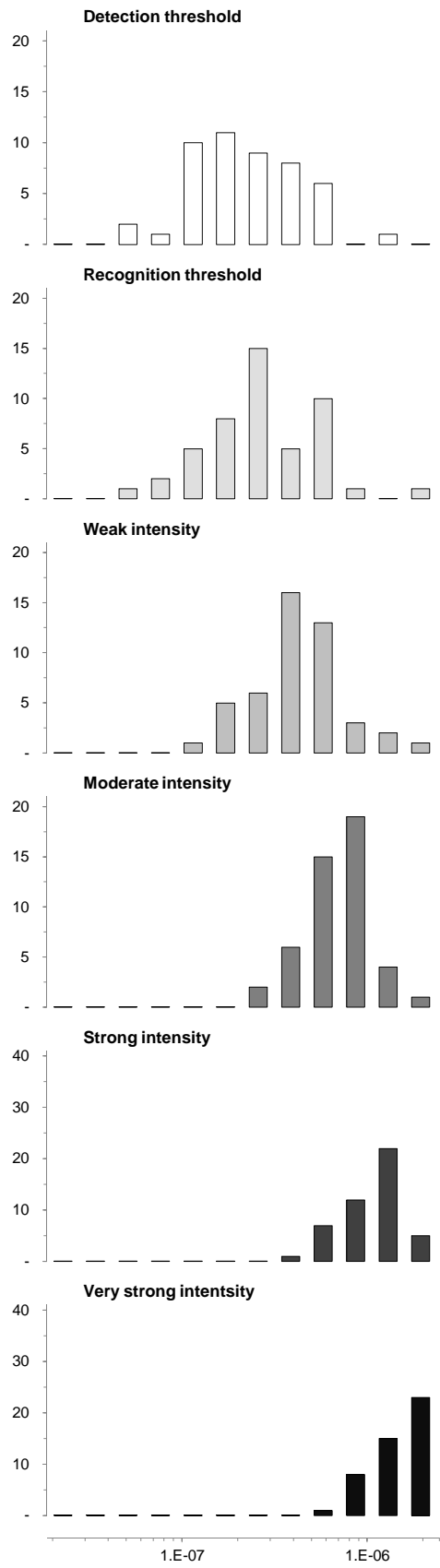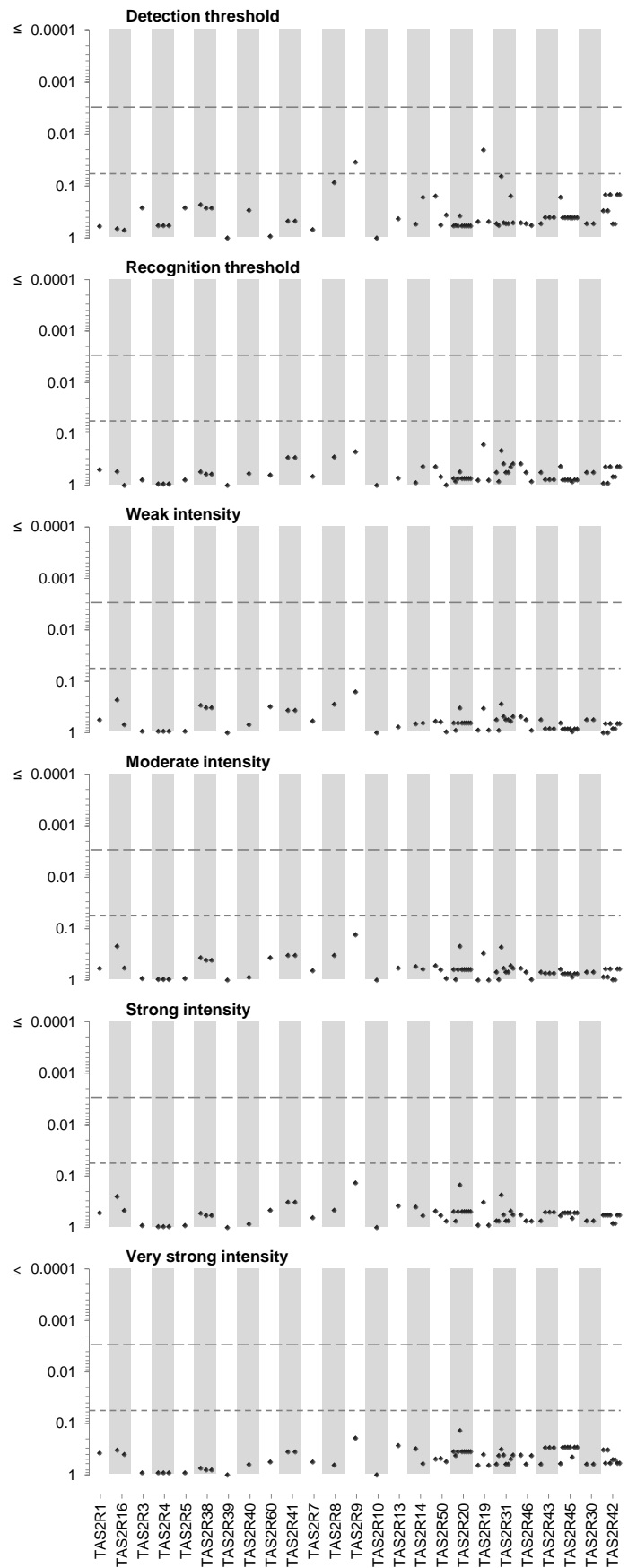

Amarogentin

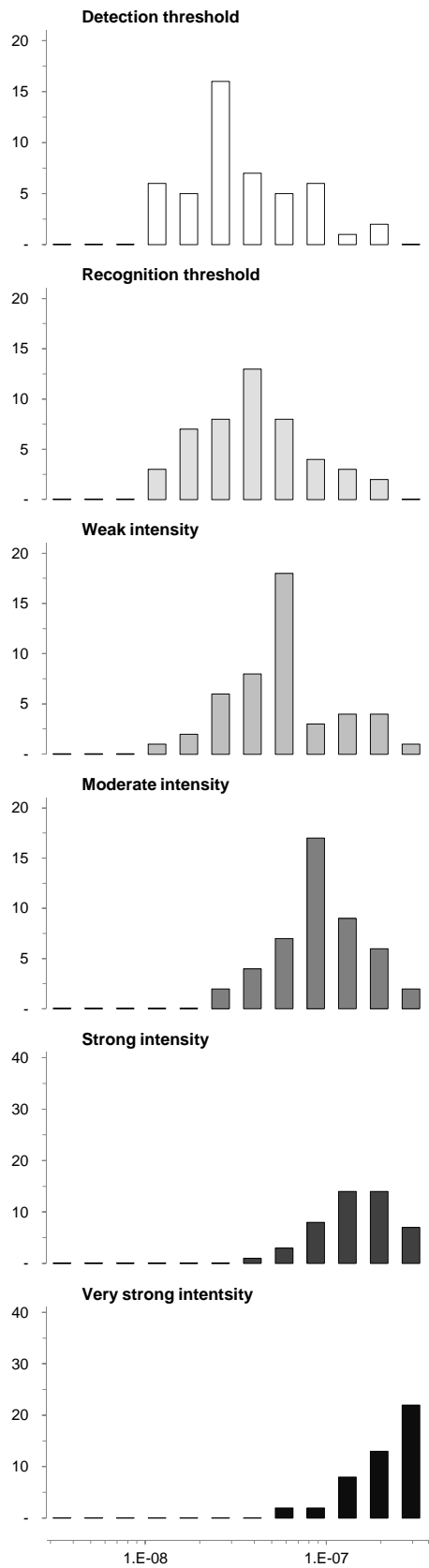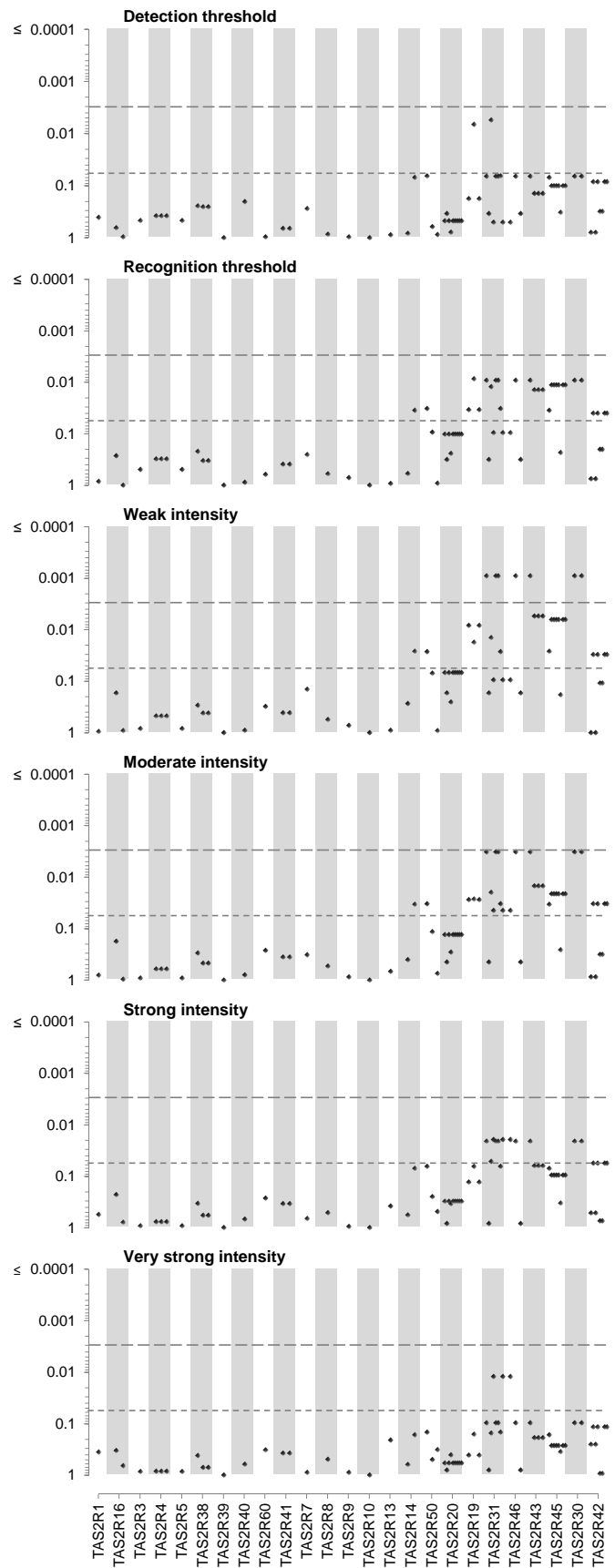

Cascarillin

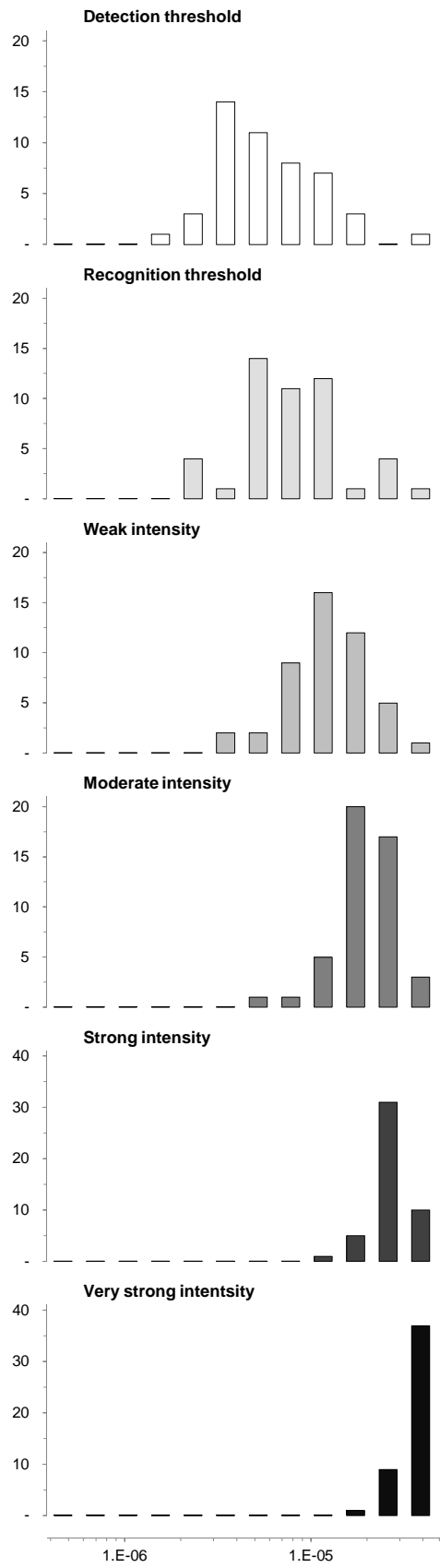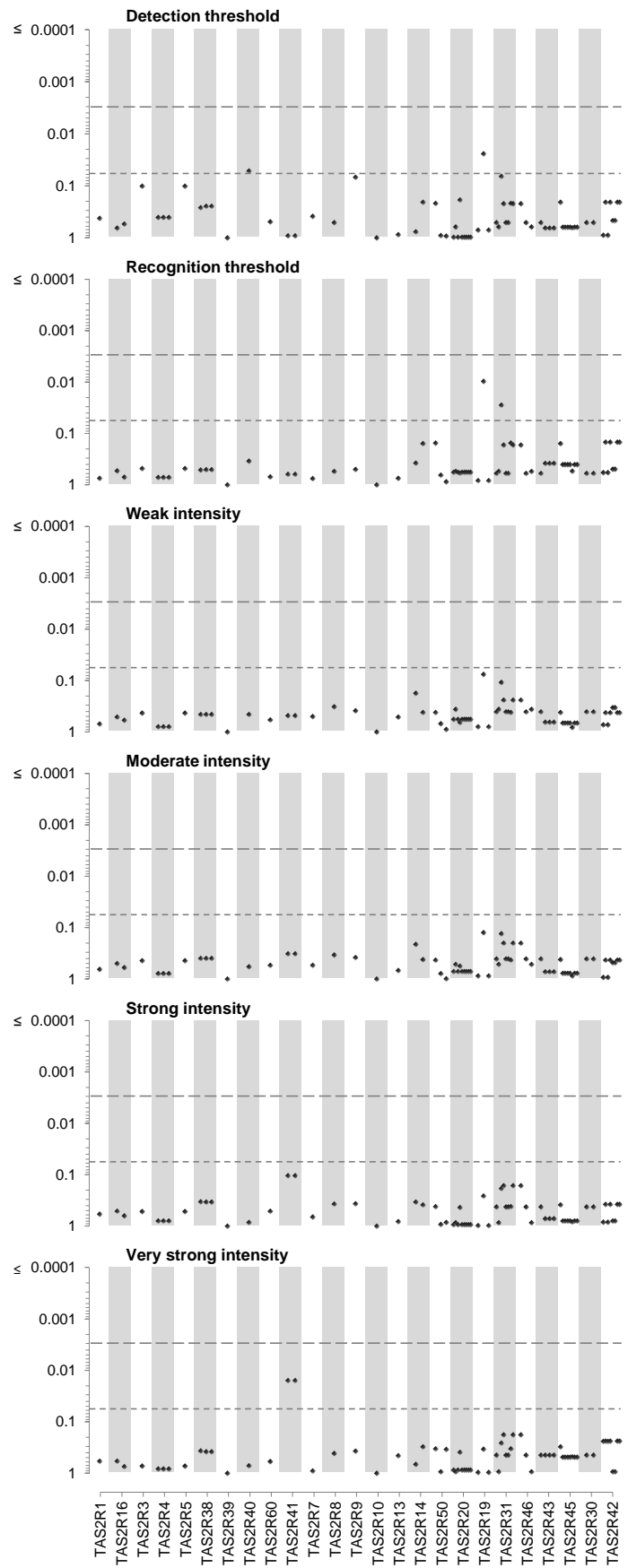

Grosheimin

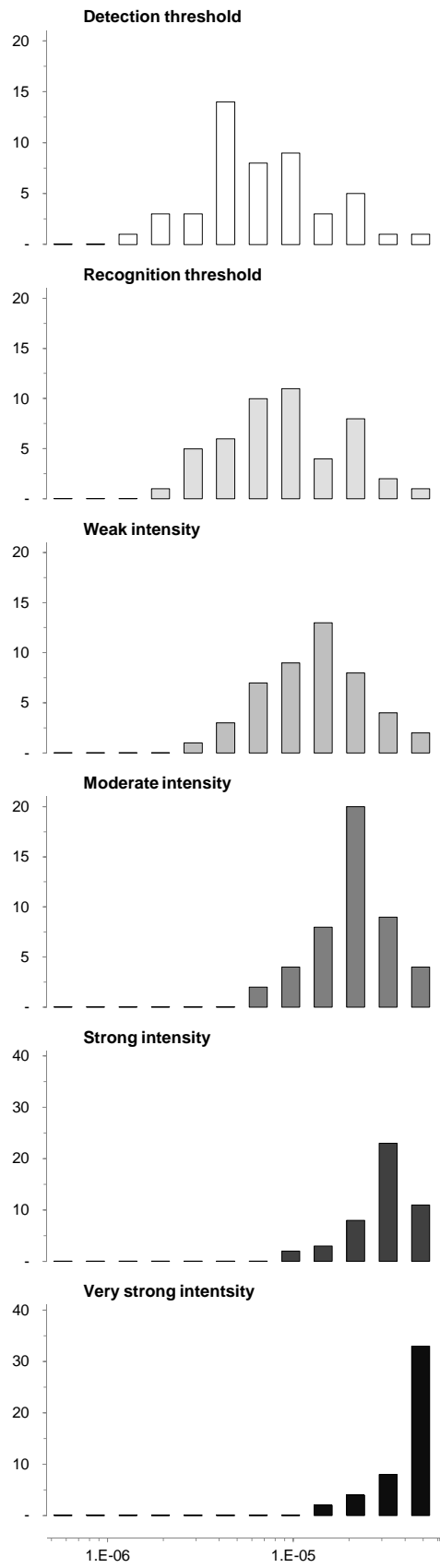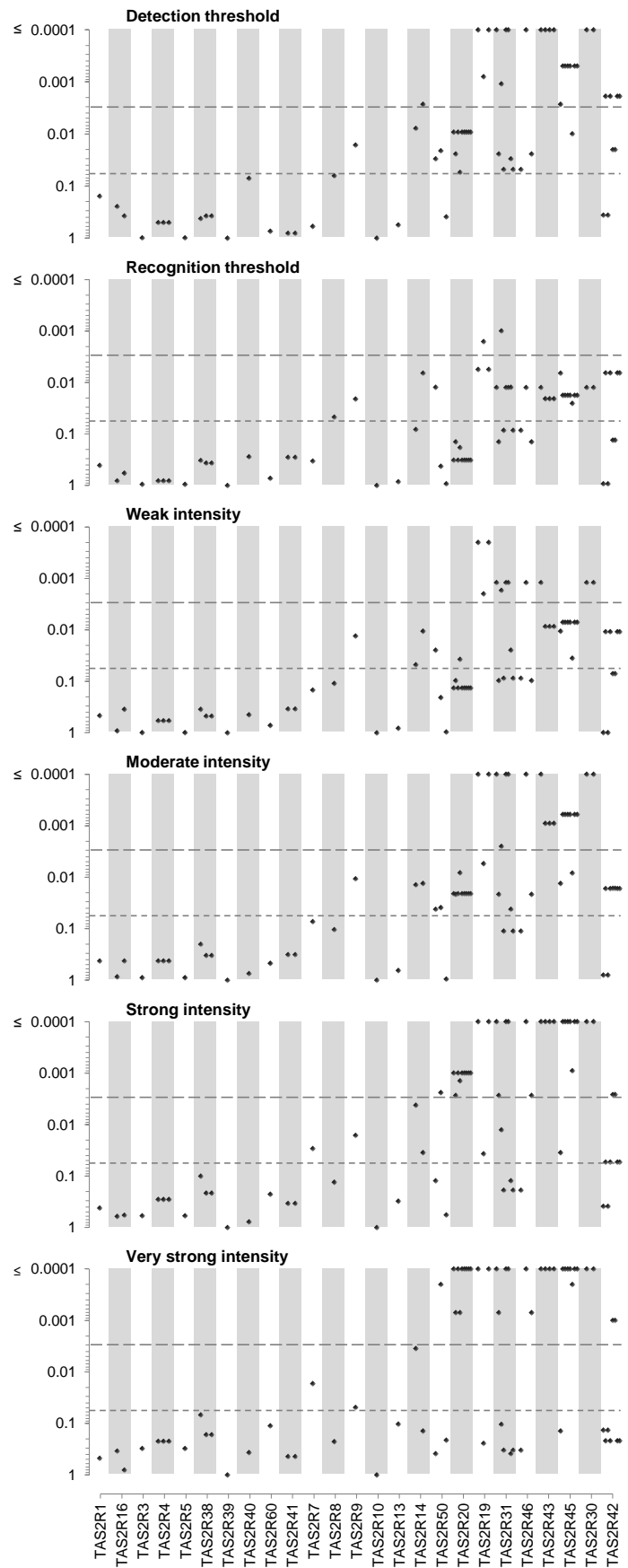

Quassin

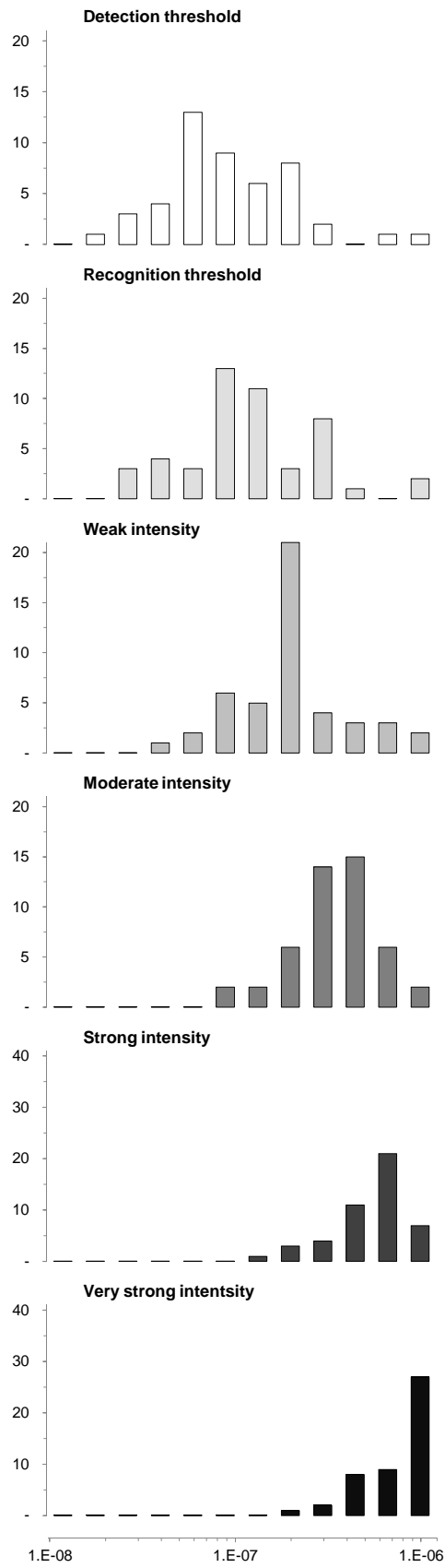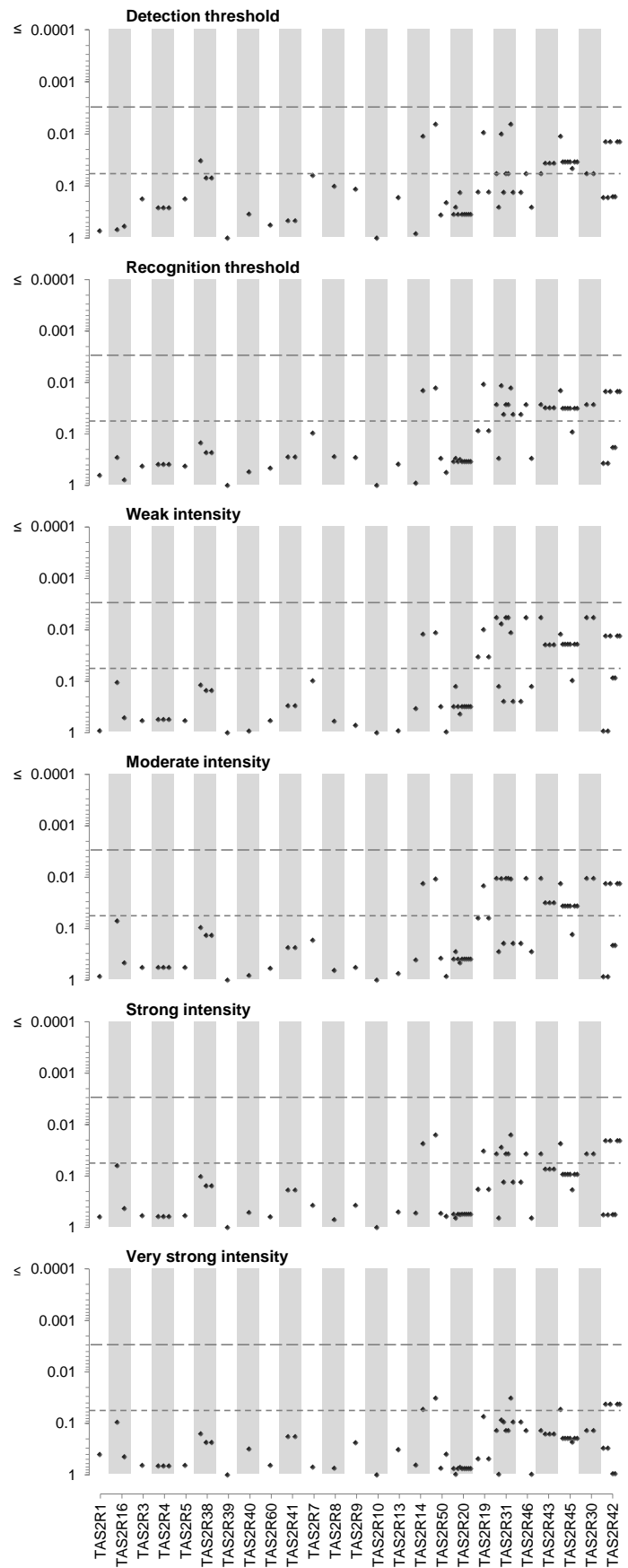

Quinine

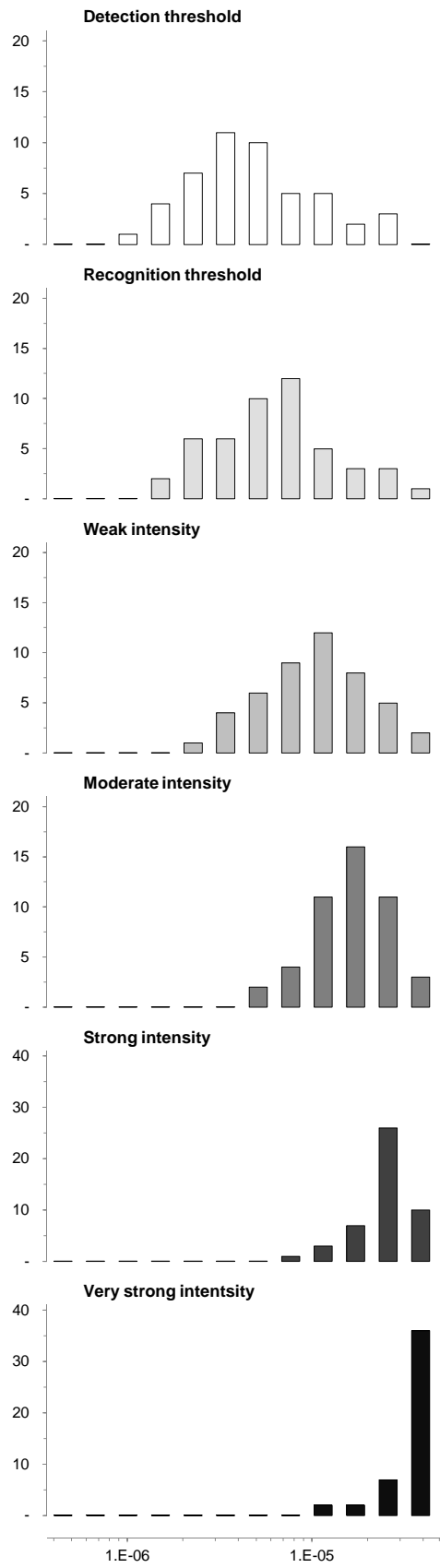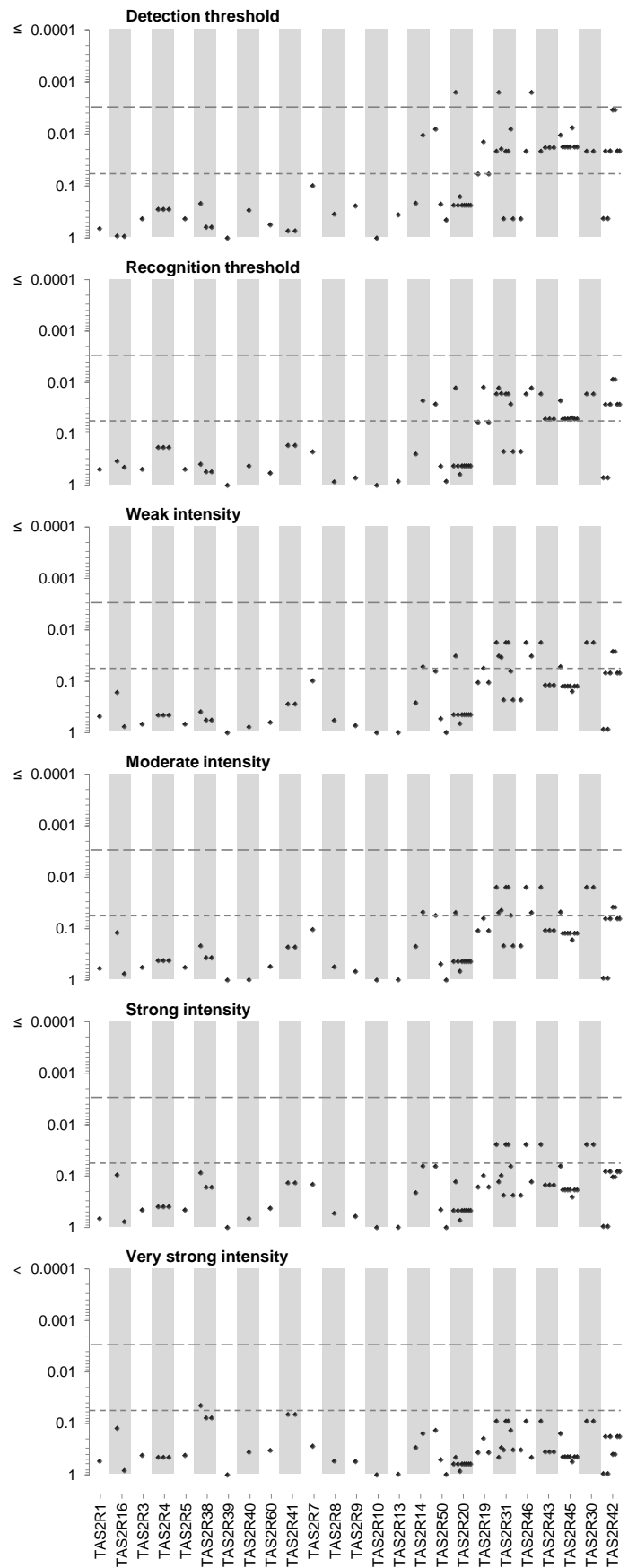

Supplement: S2 Fig — Distribution of the subjects is plotted for detection and recognition thresholds, as well as for concentrations corresponding to weak, moderate, strong, and very strong bitter taste intensities (left panel). Identically, genotype-phenotype associations for each common SNP are specified for detection and recognition thresholds, as well as for concentrations corresponding to weak, moderate, strong, and very strong bitter taste intensities (right panel). Significance level of 0.05 (dotted line), or at the experiment-wide significance threshold required to keep a significance level of 0.05 (dashed line) are specified. (PDF) [file pgen.1005530.s004.pdf]

Cascarillin

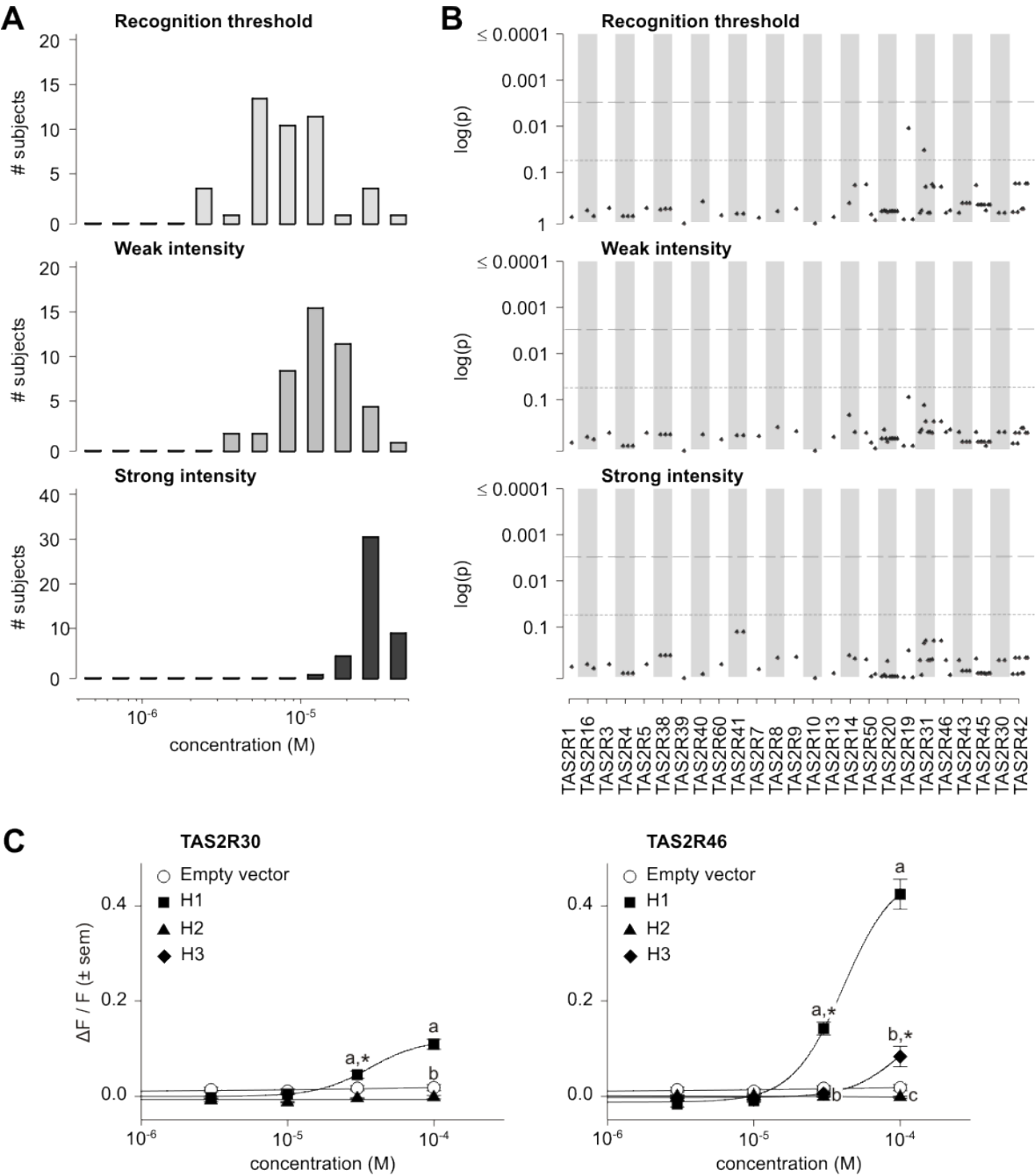

Quassin

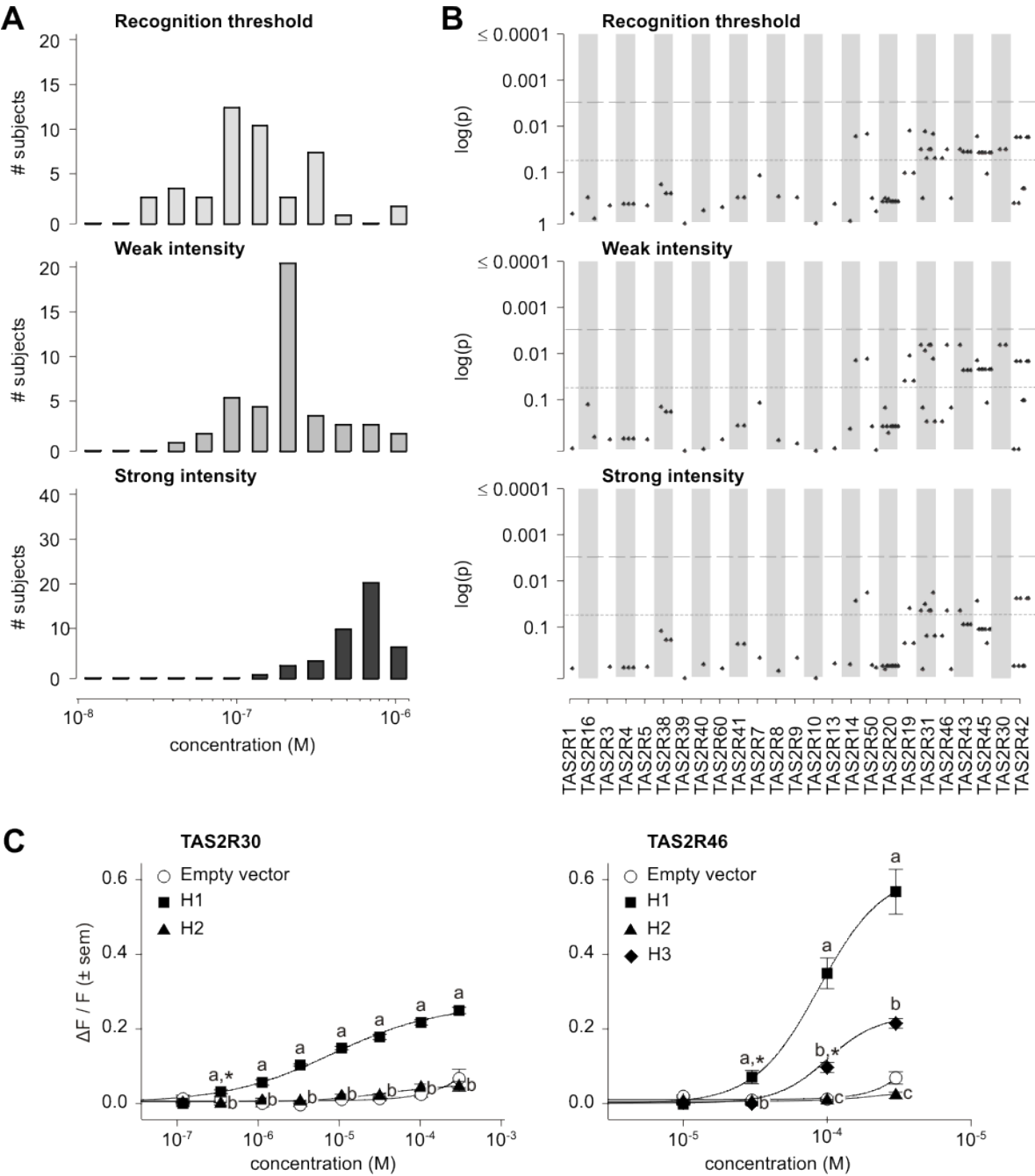

Quinine

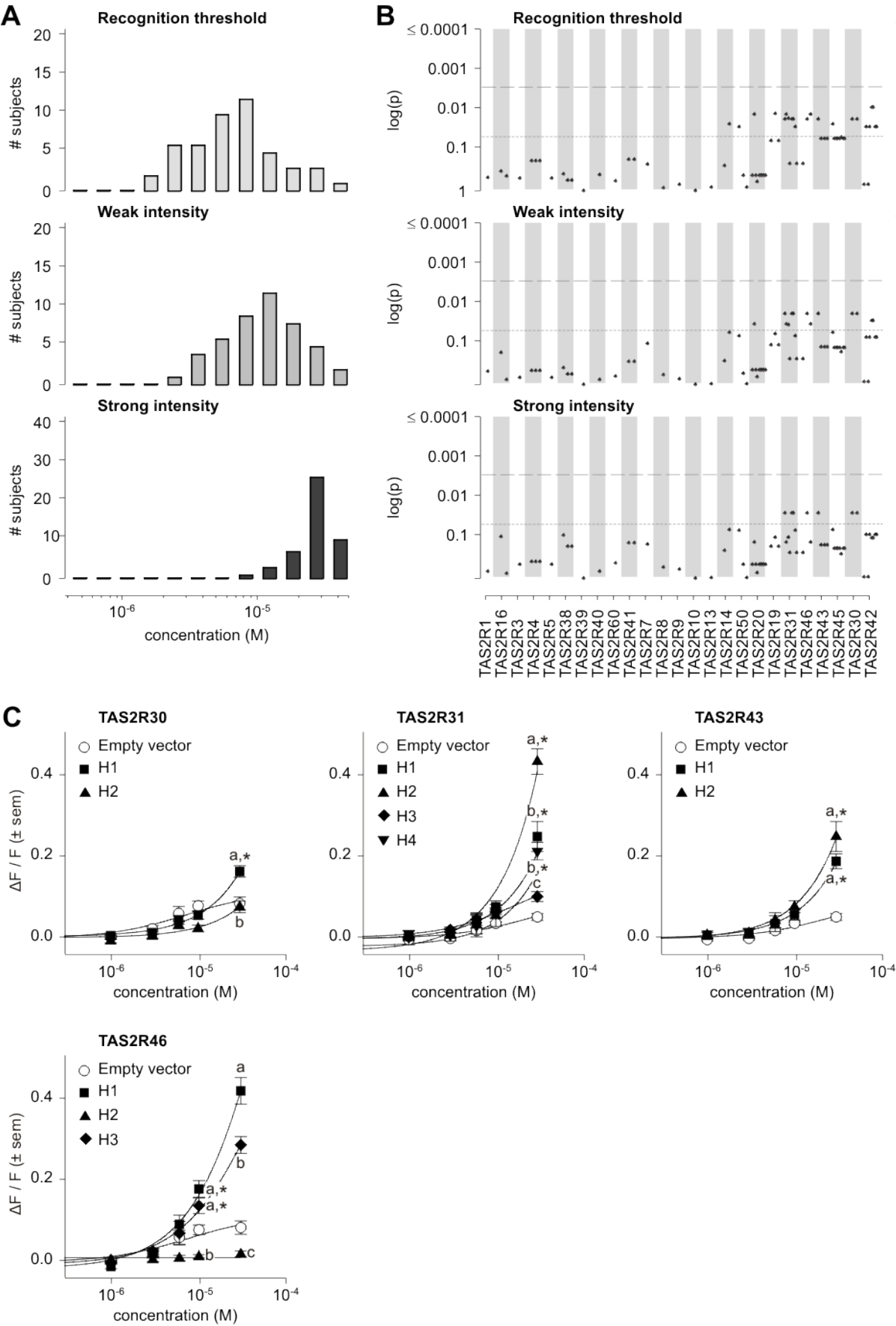

Supplement: S3 Fig — (A) Distribution of the subjects is plotted for recognition thresholds, as well as for concentrations corresponding to weak and strong bitter taste intensities. (B) Identically, genotype-phenotype associations for each common SNP are specified for recognition thresholds, as well as for concentrations corresponding to weak and strong bitter taste intensities. Significance level of 0.05 (dotted line), or at the experiment-wide significance threshold required to keep a significance level of 0.05 (dashed line) are specified. (C) Variants of TAS2R candidates were functionally challenged in heterologous cell-based assays. (PDF) [file pgen.1005530.s005.pdf]

12p13

11.0 Mb

*TAS2R46*

*TAS2R43*

*TAS2R45*

PCR 1

PCR 2

PCR 3

PCR 4

PCR 5

PCR 6

PCR 7

PCR 8

*TAS2R43* Deletion

*TAS2R45* Deletion

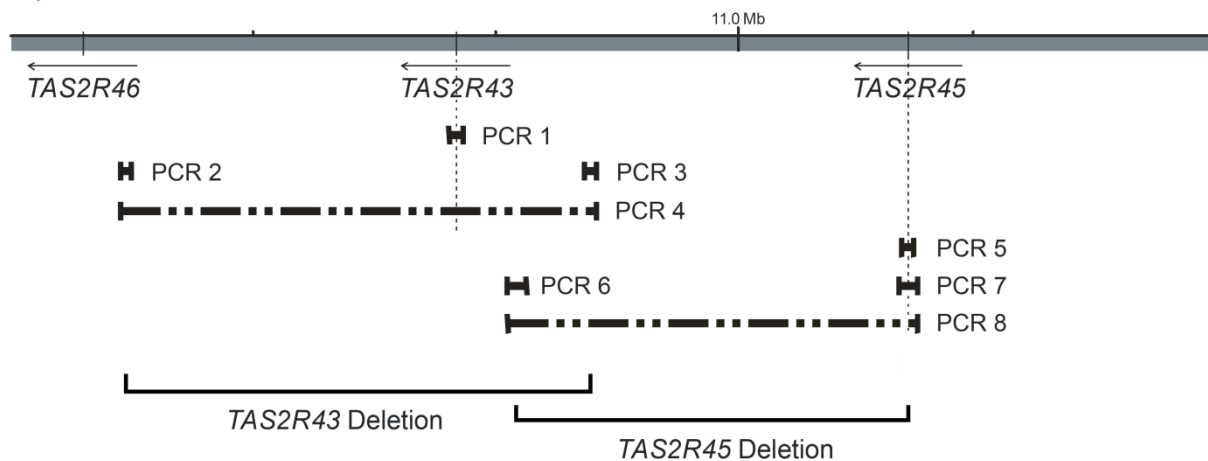

Supplement: S4 Fig — Major overlapping deletions of ~39kb and ~32kb in length were identified at the TAS2R43 locus and at the TAS2R45 locus, respectively. Consistent with these findings, PCR with primers in the flanking regions of TAS2R43 (PCR1) or TAS2R45 (PCR5) failed. Multiplex PCR distinguished subjects with zero, one or two copies of each gene. The sole presence of DNA fragments spanning a deletion indicates a copy number zero (PCR4 at TAS2R43 locus; PCR8 at TAS2R45 locus), whereas the sole presence of DNA fragments obtained with primers located within a potentially deleted DNA region indicates a copy number of two (PCR2 and PCR3 at TAS2R43 locus; PCR6 and PCR7 at TAS2R45 locus). The presence of both kinds of DNA fragments indicates a copy number one. (PDF) [file pgen.1005530.s006.pdf]
