# Supplementary material for: Receptor Polymorphism and Genomic Structure Interact to Shape Bitter Taste Perception
Source: PLoS Genet. 2015 Sep 25;11(9):e1005530. doi: 10.1371/journal.pgen.1005530 (PMC4583475; doi:10.1371/journal.pgen.1005530)

absinthin

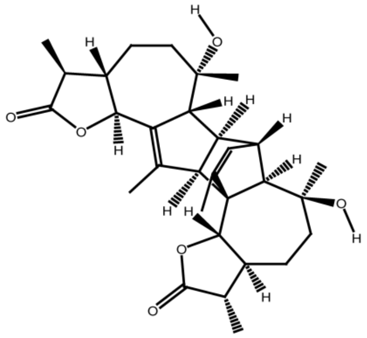

cascarillin

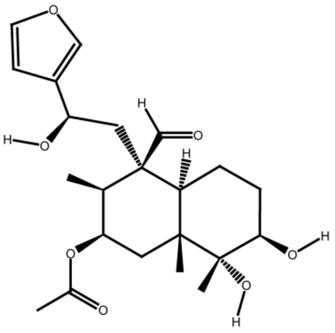

quassin

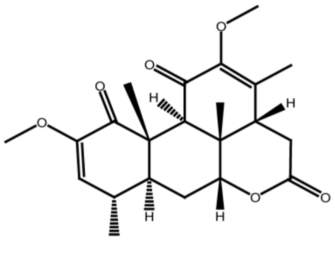

amarogentin

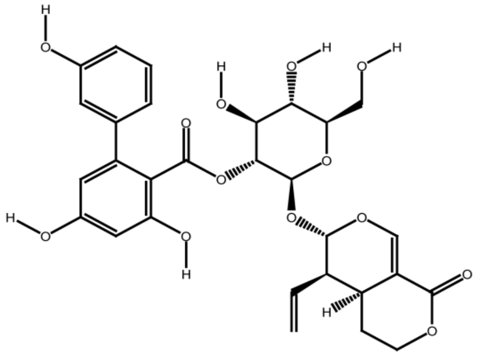

grosheimin

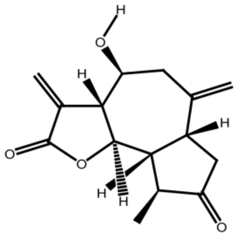

quinine

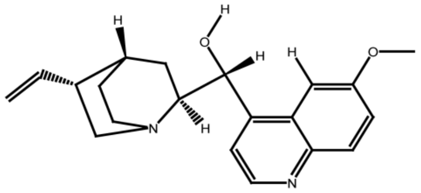

Supplement: S1 Fig — Formulas are detailed for absinthin, amarogentin, cascarillin, grosheimin, quassin, and quinine. (PDF) [file pgen.1005530.s003.pdf]
